# Chinese medicine interventions for cerebral small vessel disease-related cognitive impairment: a systematic review and meta-analysis

**DOI:** 10.3389/fphar.2026.1894066

**Published:** 2026-08-28

**Authors:** Miaomiao Zhao, Xiangyi Zheng, Yulin Mu, Genming Zhang, Xinxing Lai

**Affiliations:** 1 Dongzhimen Hospital, Beijing University of Chinese Medicine, Beijing, China; 2 Department of Neurology, Dongzhimen Hospital, Beijing University of Chinese Medicine, Beijing, China; 3 Institute for Brain Disorders, Beijing University of Chinese Medicine, Beijing, China

**Keywords:** cerebral small vessel disease, Chinese medicine interventions, cognitive impairment, meta-analysis, systematic review

## Abstract

**Background:**

Cerebral small vessel disease–related cognitive impairment (CSVD-CI) is a common and clinically important challenge of cerebral small vessel disease, but effective therapeutic options remain limited. Chinese medicine interventions (CMIs) may be beneficial, although the evidence is uncertain.

**Purpose:**

To evaluate the efficacy and safety of CMIs for adults with CSVD-CI and assess evidence certainty.

**Study design:**

Systematic review and meta-analysis of randomized controlled trials.

**Methods:**

Eight databases were systematically searched from inception to 31 December 2025. Primary outcomes were cognitive function, mainly assessed by the Montreal Cognitive Assessment (MoCA) and Mini-Mental State Examination (MMSE). Secondary outcomes included total effective rate, Traditional Chinese Medicine syndrome score, selected biochemical and hemorheological indicators, and adverse events (AEs). Risk of bias was assessed using the Cochrane Risk of Bias tool, and certainty of evidence was evaluated using GRADE.

**Results:**

Fifty RCTs involving 4,454 participants were included. Compared with conventional therapy alone, CMIs plus conventional therapy were associated with higher end-of-treatment MoCA and MMSE scores, and the pooled incidence of AEs did not differ significantly between the intervention and control groups. The overall risk of bias was predominantly unclear, whereas the certainty of evidence was low or very low.

**Conclusion:**

CMIs were associated with higher end-of-treatment cognitive scores; however, extreme heterogeneity, unclear risk of bias, and low evidence certainty substantially limit confidence in these findings. Available data are insufficient to establish long-term efficacy or safety. Multicenter, prospectively registered RCTs with standardized diagnostic criteria, intervention reporting, outcome selection, and safety monitoring are required.

**Systematic review registration:**

https://www.crd.york.ac.uk/PROSPERO/view/CRD420251176394, identifier, CRD420251176394.

## 1 Introduction

Cerebral small vessel disease (CSVD) is a heterogeneous group of pathological processes affecting the small arteries, arterioles, capillaries, and venules of the brain. The most common etiologies are arteriolosclerosis associated with aging, hypertension, and other conventional vascular risk factors, and cerebral amyloid angiopathy caused by vascular β-amyloid deposition; less common causes include monogenic, inflammatory, venous, and post-radiation arteriopathies (Dupré et al., 2024; Litak et al., 2020; Markus and de Leeuw, 2023). The most characteristic neuroimaging markers of CSVD include white matter hyperintensities (WMH), lacunes, cerebral microbleeds (CMB), and enlarged perivascular spaces (EPVS) (Duering et al., 2023). CSVD is frequently clinically covert, and its reported prevalence varies with age, population characteristics, MRI protocols, and lesion thresholds. In the population-based PRECISE study, 30.5% of 3,063 community-dwelling Chinese adults aged 50–75 years had at least one major CSVD imaging marker, and older age and hypertension were independently associated with greater CSVD burden (Blumen et al., 2023; Yang et al., 2023). The population at risk is expected to expand substantially over the next 10–20 years: the global population aged 60 years or older is projected to reach 1.4 billion by 2030 (World Health Organization, 2025), China is expected to have approximately 400 million people aged 60 years or older by around 2035 (State Council of the People’s Republic of China. 2023), and the worldwide number of people living with dementia is projected to reach 83.2 million in 2030 and 116.0 million in 2040 (GBD, 2019 Dementia Forecasting Collaborators, 2022; Ma et al., 2024). Although these demographic and all-cause dementia projections are not direct estimates of future CSVD cases, the strong age-dependence of CSVD indicates that both the population at risk and the associated cognitive-care burden are likely to increase. Among these, CSVD-related cognitive impairment (CSVD-CI) commonly affects multiple cognitive domains, resulting in declines in executive function, attention, and information processing speed (Barucci et al., 2024a; Barucci et al., 2024b). These impairments can compromise activities of daily living and are associated with unfavorable long-term outcomes, resulting in a substantial burden on patients, families, and healthcare systems (Jin et al., 2025; Sun et al., 2025). Against this background, the treatment and prognostic management of CSVD-CI have attracted increasing attention.

In current clinical practice, evidence-based strategies for CSVD-CI primarily focus on secondary stroke prevention and comprehensive vascular risk factor control. These strategies include intensive blood pressure management, lipid-lowering therapy, and antiplatelet therapy, together with lifestyle interventions such as smoking cessation, regular physical activity, and dietary modification (Sun et al., 2025; Wardlaw et al., 2021). On this basis, some patients additionally receive pharmacological agents targeting cognitive symptoms, such as cholinesterase inhibitors, N-methyl-D-aspartate (NMDA) receptor antagonists and so on (Ip et al., 2024; Lennon and Sachdev, 2026). However, available studies have not yet provided established evidence to demonstrate that these interventions confer clear and specific cognitive benefits in CSVD-CI, and several of these drugs are associated with an increased incidence of adverse effects (Masserini et al., 2025; Zanon Zotin et al., 2021). Consequently, identifying therapeutic strategies that can further optimize cognitive outcomes in patients with CSVD-CI while ensuring long-term safety remains a major challenge in current clinical practice and research.

In China and other regions, Chinese medicine interventions (CMIs) are increasingly regarded as potential adjunctive or alternative therapies for CSVD-CI. From the perspective of traditional Chinese medicine (TCM), CSVD-CI is commonly associated with patterns such as “qi deficiency and blood stasis” and dysfunction of the zang-fu organs, which broadly reflect impaired systemic regulation, disturbed circulation, and internal homeostatic imbalance. On this basis, CMIs often include individualized, multi-medicinal-material prescriptions tailored to syndrome differentiation, through regulating qi and blood and supporting organ-system function and ultimately improving patients’ clinical symptoms and functional status. From a modern pharmacological standpoint, Chinese medicine preparations and multi-medicinal-material formulations contain numerous bioactive substances and may exert multi-target, multi-pathway effects, including coordinated modulation of neurovascular unit function, inflammatory and oxidative stress responses, energy metabolism, and synaptic function and neuroplasticity, thereby potentially influencing cognitive outcomes in patients with CSVD-CI (Lv et al., 2023; Ma et al., 2025).

In recent years, a number of randomized controlled trials (RCTs) have investigated the efficacy and safety of CMIs, either alone or in combination with conventional therapy (CT), in patients with CSVD or CSVD-related cognitive impairment. Moreover, considerable heterogeneity persists across existing studies in terms of Chinese medicine formulations, intervention duration, control conditions, outcome measures, and follow-up periods. Consequently, despite a growing body of clinical evidence, the overall strength, consistency, and clinical applicability of the available findings remain uncertain, highlighting the need for a comprehensive and systematic synthesis of current RCT evidence.

Objective: The present study aimed to conduct a systematic review and meta-analysis of RCTs evaluating CMIs for CSVD-CI and to provide evidence to support clinical decision-making and future research through a comprehensive evaluation of cognitive outcomes, safety, risk of bias, and certainty of evidence.

## 2 Methods

### 2.1 Study design and reporting standard

This study was designed, conducted, and reported in accordance with the Preferred Reporting Items for Systematic Reviews and Meta-Analyses (PRISMA) 2020 statement (Page et al., 2021). The review protocol was prospectively registered in PROSPERO (registration number: CRD420251176394).

### 2.2 Eligibility criteria

#### 2.2.1 Inclusion criteria

The eligibility criteria were formulated according to the PICOS framework.

1. Participants: Eligible participants were adult patients clinically diagnosed with cerebral small vessel disease–related cognitive impairment (CSVD-CI). No restrictions were imposed with regard to sex, age, race, or route of admission.
2. Intervention: The intervention group received CMIs, with no restrictions on the type of medicinal materials, Chinese medicine formulations, or dosage forms, including decoctions, granules, capsules, oral liquids, or injections. Dosage and routes of administration were not restricted. If the intervention group included treatments other than CMIs, the control group was required to receive the same treatments.
3. Comparators: Control interventions varied according to study design and included CT alone, biomedicine plus CT, or placebo plus CT.
4. Outcomes: The primary outcomes were cognitive function assessed using the Montreal Cognitive Assessment (MoCA) and Mini-Mental State Examination (MMSE). Endpoint values at the prespecified assessment time points were used for the meta-analysis. Secondary outcomes included total effective rate; Traditional Chinese Medicine (TCM) syndrome score; biochemical markers of inflammation and oxidative stress, namely homocysteine (HCY), high-sensitivity C-reactive protein (hs-CRP), and malondialdehyde (MDA); hemorheological indicators, namely whole blood viscosity at high shear rate (WBV-H), whole blood viscosity at low shear rate (WBV-L), and plasma viscosity (PV); and adverse events (AEs).
5. Study design: Only randomized controlled trials (RCTs) were eligible for inclusion.

#### 2.2.2 Exclusion criteria

The following studies were excluded: (1) studies in which the intervention administered to the intervention group did not involve CMIs; (2) studies with inappropriate designs in which the CTs received by the intervention and control groups were different; (3) studies that did not meet the methodological criteria for RCT design; (4) non-original clinical studies, including conference abstracts, academic theses, review articles, animal or cell experiments and meta-analysis; (5) duplicate publications or studies based on the same dataset, in which case only one published study was included; (6) studies that did not report the outcomes of interest; (7) and studies with a treatment duration shorter than 4 weeks.

### 2.3 Information sources and search strategy

A systematic search was conducted in PubMed, the Cochrane Library, Embase, Web of Science, China National Knowledge Infrastructure (CNKI), Wanfang Database, VIP Database, and SinoMed. The search period covered inception of each database to 31 December 2025, with no language restrictions. Core search terms included “cerebral small vessel disease,” “leukoencephalopathies,” “white matter,” “cognitive decline,” “cognitive impairment,” “Chinese herbal medicine,” “randomized controlled trial,” and related terms.

Literature retrieval, reference management, and study selection were independently performed by two reviewers (MZ, YM). Titles and abstracts were screened initially, followed by full-text assessment of potentially eligible studies. Any disagreements were resolved through discussion, and a third reviewer (XZ) was consulted when necessary. The detailed search strategy is provided in Supplementary Appendix A.

### 2.4 Data extraction

EndNote 20 was used to manage the retrieved records, remove duplicates, and support the screening process. After finalizing the list of included studies, two reviewers (MZ, YM) independently extracted data from all eligible trials. Extracted information included basic study characteristics (authors and year of publication), participant characteristics (sample size, mean age, sex distribution, and country or region), details of the intervention and comparator (name, dosage, formulation, and treatment duration), and outcome data (outcome measures, assessment time points, and numerical results). All biochemical markers reported in the eligible RCTs were extracted, and only those with sufficient and comparable data across studies were included in the quantitative synthesis. Discrepancies in data extraction were resolved through discussion with a third reviewer (XZ).

### 2.5 Risk of bias assessment

The risk of bias of the included studies was independently assessed by two reviewers (MZ, XZ) using the Cochrane Risk of Bias tool for RCTs (Higgins et al., 2011). The assessment covered seven domains: random sequence generation, allocation concealment, blinding of participants and personnel, blinding of outcome assessment, incomplete outcome data, selective reporting, and other bias. Each domain was rated as low risk, high risk, or unclear risk. Disagreements were resolved by discussion with a third reviewer (XL).

### 2.6 Statistical analyses

Statistical analyses were performed using Review Manager (RevMan) version 5.4 and Stata version 19. Continuous outcomes were expressed as mean differences (MDs) with 95% confidence intervals (CIs) when measured using the same scale and scoring system, or as standardized mean differences (SMDs) with 95% CIs when different or non-equivalent scales were used; dichotomous outcomes were expressed as risk ratios (RRs) with 95% CIs. Statistical heterogeneity was assessed using Cochran’s chi-square test and quantified using the I^2^ statistic. When I^2^ was ≤50%, a fixed-effect model was applied; otherwise, a random-effects model was used.

Studies were considered eligible for each synthesis only when they were sufficiently comparable in terms of participant characteristics, intervention and comparator type, outcome measure, and assessment time point. Before quantitative synthesis, outcome data were checked and standardized where necessary to ensure consistency in outcome definition, unit of measurement, and direction of effect. Outcomes assessed at different time points were not pooled together unless considered clinically comparable. When required data were incomplete or unavailable, the study was excluded from the corresponding meta-analysis and summarized descriptively instead. No imputation of missing summary statistics was performed.

When I^2^ exceeded 50% for primary outcomes and sufficient data were available, subgroup analyses were conducted to explore potential sources of heterogeneity. Prespecified subgroup factors included treatment duration, with or without concomitant biomedicine therapy, dosage form, and the use of commercial Chinese polyherbal preparations (CCPPs). Treatment-duration categories were based on the treatment-course intervals most commonly represented among the included trials, while avoiding sparsely populated subgroups. Concomitant biomedicine was considered because background treatment, even when administered equally to both groups, may modify the incremental effect of Chinese medicine interventions through additive, interactive, or ceiling effects. Dosage form was evaluated because decoctions, granules, capsules, and tablets may differ in preparation, bioactive-substance concentration, bioavailability, dosing consistency, and adherence. CCPP status was examined separately because it reflects the degree of standardization in composition, manufacturing, and dosing rather than merely the physical form of administration. Other potentially relevant variables could not be assessed reliably because of inconsistent reporting or insufficient study-level data. All subgroup analyses were considered exploratory. The eight subgroup interaction tests conducted across the two outcomes were considered a single family, yielding a Bonferroni-corrected significance threshold of 0.00625 (0.05/8).

To further investigate potential contributors to between-study heterogeneity, exploratory univariable and multivariable random-effects meta-regression analyses were performed using Stata with restricted maximum likelihood (REML) estimation. Univariable meta-regression models were first conducted to examine the associations between prespecified study-level characteristics and effect estimates. Variables selected based on clinical relevance and/or potential associations in univariable analyses were subsequently entered into multivariable meta-regression models. The investigated moderators included sample size, treatment duration, CCPP status, and concomitant biomedicine therapy.

Sensitivity analyses were conducted using a leave-one-out approach, in which each study was sequentially excluded and the meta-analysis was repeated to assess the robustness of the pooled estimates. In addition, funnel plots were generated to qualitatively assess potential publication bias or small-study effects when at least 10 studies were available for a given outcome.

### 2.7 Certainty of evidence (GRADE)

Major outcomes were assessed using the Grading of Recommendations Assessment, Development and Evaluation (GRADE) approach by two reviewers (ZMM, ZXY) independently (Guyatt et al., 2008). Furthermore, evidence certainty was evaluated based on risk of bias, inconsistency, indirectness, imprecision, and publication bias, and classified as high, moderate, low, or very low. For outcomes including 10 or more studies, publication bias was further assessed using Egger’s test. Any disagreements were resolved through discussion, and a third reviewer (XL) was consulted if consensus could not be reached.

## 3 Results

### 3.1 Study selection

**FIGURE 1:**
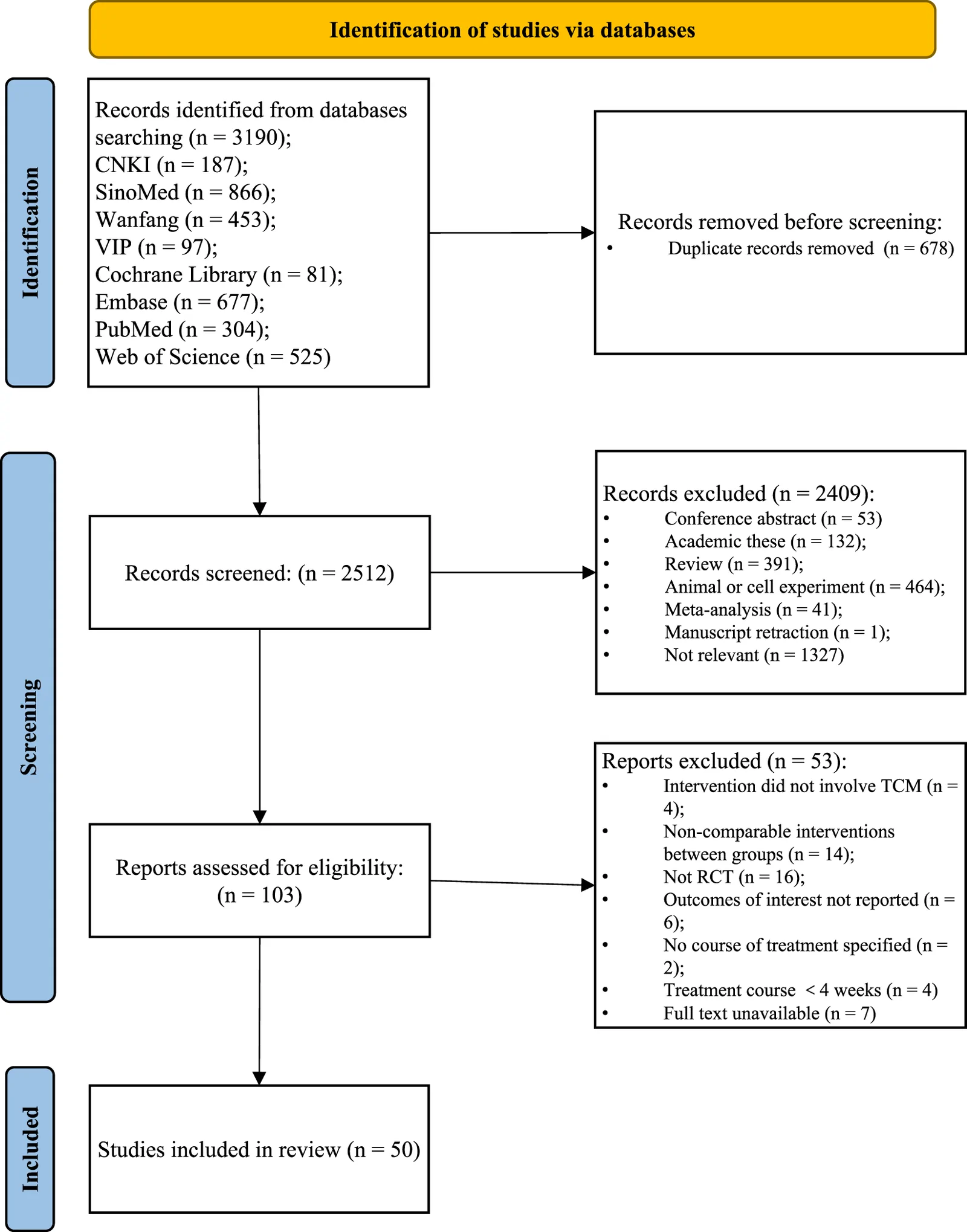
PRISMA flow diagram of the study selection process.

A total of 3,190 records were retrieved from eight databases. After removing 678 duplicates, 2,512 records were screened by title and abstract, of which 2,409 were excluded. The full texts of 103 articles were further assessed for eligibility, and 53 studies were excluded for predefined reasons. Finally, 50 RCTs were included in this systematic review and meta-analysis. The study selection process is illustrated in Figure 1.

### 3.2 Study characteristics

A total of 50 RCTs conducted in China were included, involving 4,454 participants, with 2,224 in the treatment group and 2,230 in the control group. All studies used a two-arm parallel design, and baseline age and sex distribution were generally comparable between groups. The interventions involved CMIs administered in the context of CT, whereas control interventions varied according to study design and included CT alone, biomedicine plus CT, or placebo plus CT. Treatment duration ranged from 30 days to 6 months, with 8 weeks, 12 weeks, and 3 months being the most common intervention periods. The most frequently reported outcomes were cognitive function measures, particularly the MoCA and MMSE, followed by total effective rate, TCM syndrome score, biochemical indicators, hemorheological parameters, and AEs. CMIs were administered in various oral dosage forms, including capsules, granules, tablets, and decoctions. Further details of the included studies are presented in Supplementary Table S1.

**FIGURE 2:**
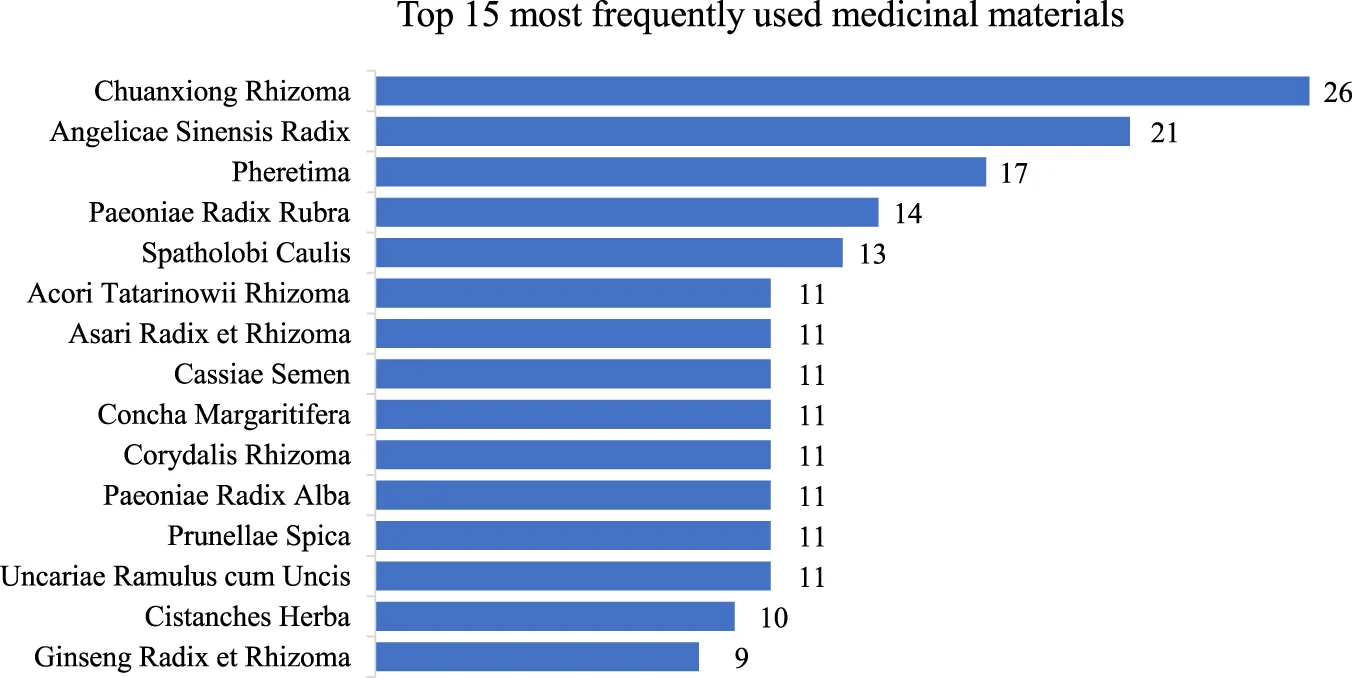
Frequency distribution of the 15 most frequently used medicinal materials across the included Chinese medicine prescriptions.

In addition, 112 distinct medicinal materials were identified across the included prescriptions, with 431 cumulative occurrences, among which Chuanxiong Rhizoma, Angelicae Sinensis Radix, Pheretima, Paeoniae Radix Rubra, and Spatholobi Caulis were the most frequently used. The distribution of the 15 most frequently used medicinal materials is shown in Figure 2. Intervention characteristics, including intervention name, dosage form, composition, dose, preparation method, treatment duration, co-interventions, sources of product information and adverse-event data, and completeness of intervention reporting, were extracted and are presented in Supplementary Table S2. The complete naming and frequency data for all 112 medicinal materials are presented in Supplementary Table S3, and the ConPhyMP-informed assessment of intervention-reporting completeness is provided in Supplementary Table S4. Nine CCPPs had clearly identifiable and traceable product information, whereas comparable information was not reported for the remaining investigator-designed prescriptions.

### 3.3 Risk of bias of included studies

**FIGURE 3:**
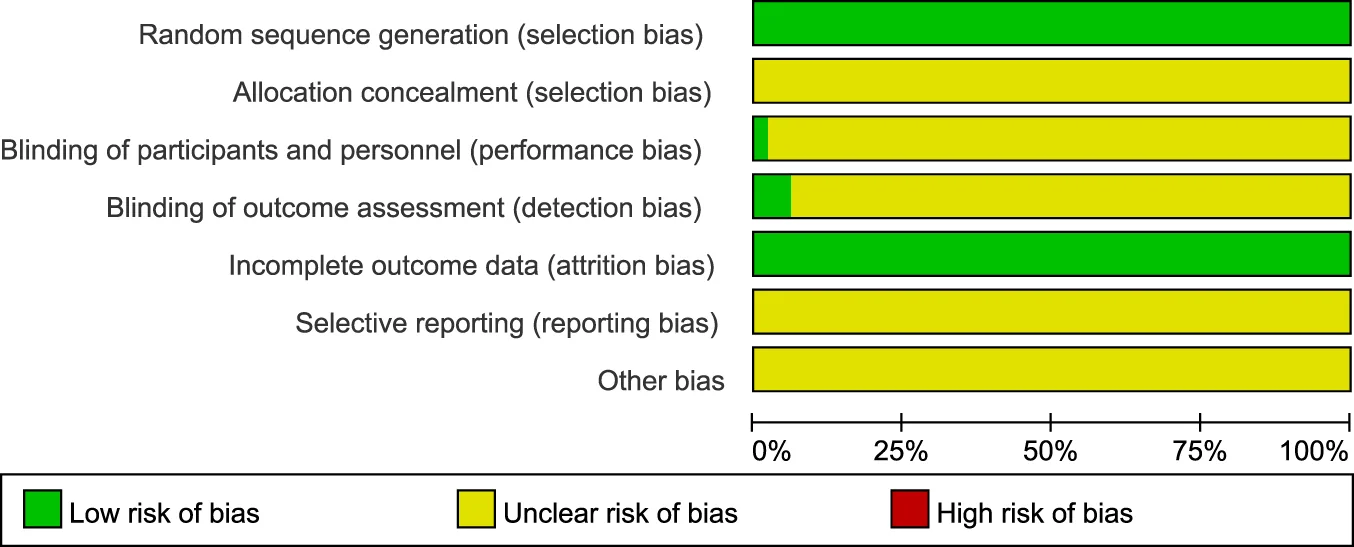
Risk-of-bias graph of the included studies.

**FIGURE 4:**
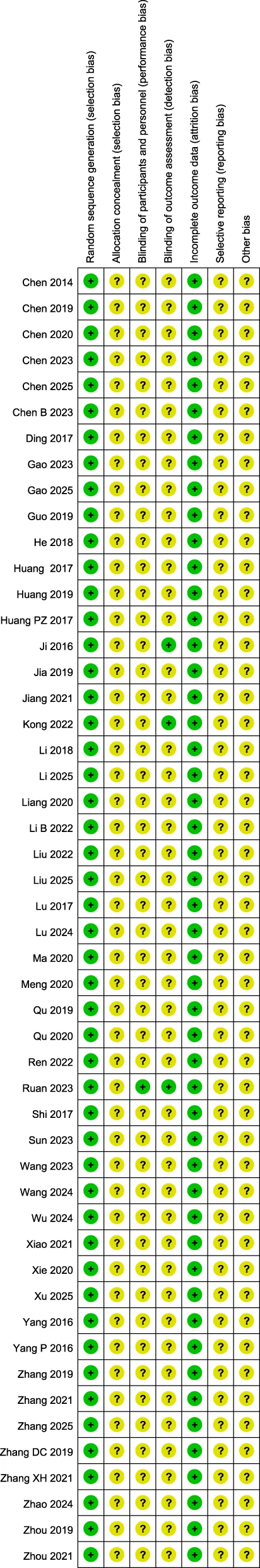
Risk-of-bias summary of the included studies.

The overall risk of bias of the included studies was judged to be unclear. Random sequence generation and incomplete outcome data were generally assessed as low risk. In contrast, allocation concealment, selective reporting, and other bias were mostly judged as unclear risk because of insufficient reporting. Blinding of participants, personnel, and outcome assessment was also largely unclear, with only a few studies providing adequate methodological details. Overall, the main limitation in methodological quality was inadequate reporting across several risk of bias domains. The risk of bias graph and summary are shown in Figures 3, 4, respectively.

### 3.4 Results of meta-analysis

Meta-analyses were stratified by comparator: (1) CMIs plus conventional therapy (CT) versus CT alone; (2) CMIs plus CT versus biomedicine plus CT; and (3) CMIs plus CT versus placebo plus CT. Estimates from these fundamentally different comparisons were not pooled together.

#### 3.4.1 Chinese medicine interventions (CMIs) plus CT versus CT alone

**FIGURE 5:**
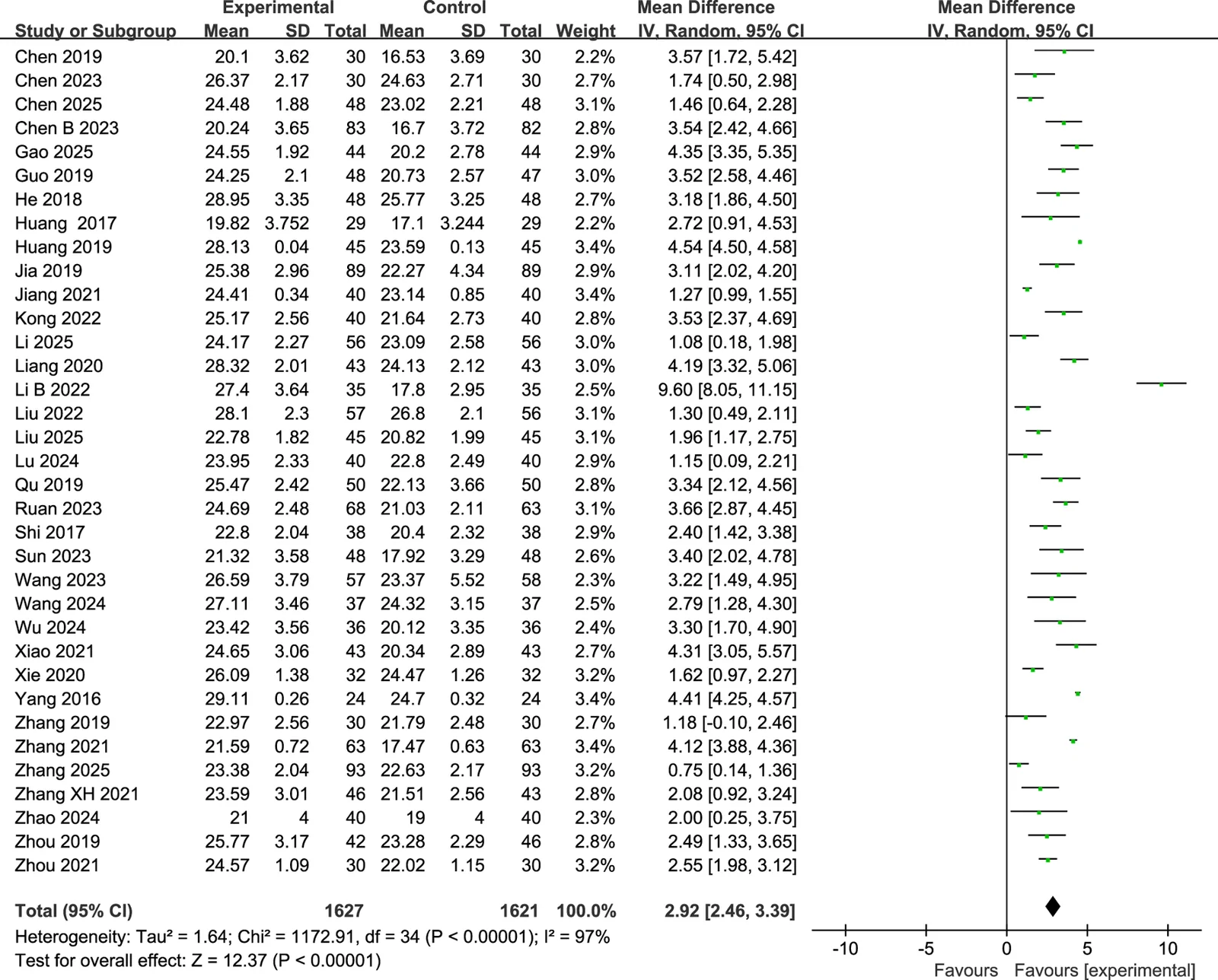
Forest plot of MoCA at the end of treatment for CMIs plus CT versus CT alone in patients with CSVD-CI.

MoCA score at the end of treatment. A total of 35 studies involving 3,248 participants assessed short-term MoCA outcomes. The pooled analysis showed that CMIs significantly improved MoCA scores in patients with CSVD-CI compared with the control group (MD = 2.92, 95% CI [2.46, 3.39], P < 0.00001; Figure 5). Substantial heterogeneity was observed across studies, with an I^2^ value of 97%, and a random-effects model was therefore used. To further explore the potential sources of heterogeneity, subgroup analyses were performed according to treatment duration, whether biomedicine was co-administered, dosage form, and CCPP status. The corresponding forest plots are provided in Supplementary Figures S1–S4. The beneficial effect of CMIs remained significant across all treatment-duration subgroups, in studies with and without biomedicine, across different dosage forms, and irrespective of CCPP status. A nominal subgroup difference was observed for dosage form (P = 0.02), however, it did not meet the Bonferroni-corrected significance threshold of 0.00625, and substantial residual heterogeneity persisted within the subgroups.

**FIGURE 6:**
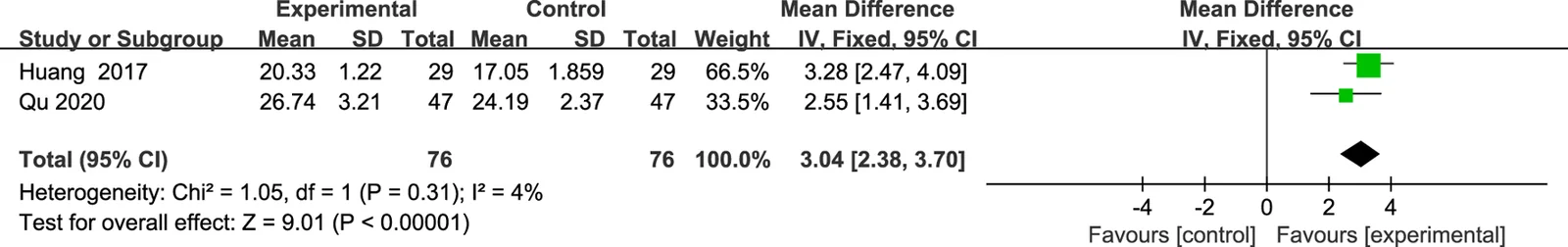
Forest plot of MoCA at 6-month follow-up for CMIs plus CT versus CT alone in patients with CSVD-CI.

MoCA score at 6-month follow-up. Two studies (Huang R.C. et al., 2017; Qu et al., 2020) involving 152 participants assessed MoCA at 6-month follow-up. The pooled analysis showed that CMIs significantly improved MoCA scores compared with the control group (MD = 3.04, 95% CI [2.38, 3.70], P < 0.00001; Figure 6). Heterogeneity across studies was low (I^2^ = 4%).

**FIGURE 7:**
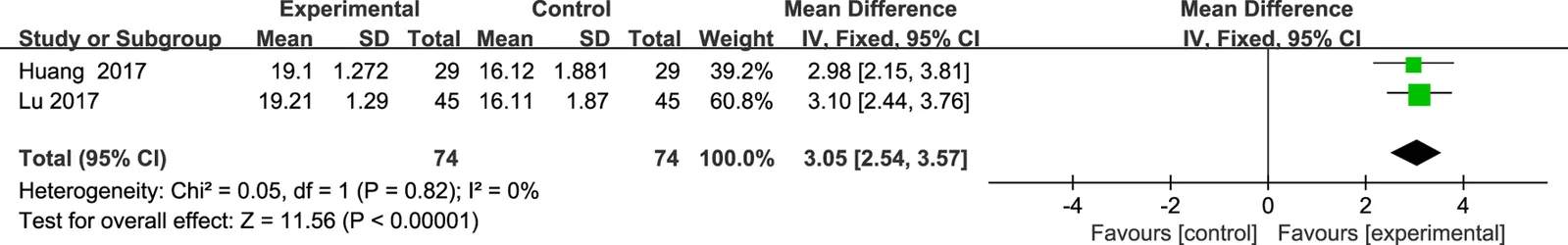
Forest plot of MoCA at 12-month follow-up for CMIs plus CT versus CT alone in patients with CSVD-CI.

MoCA score at 12-month follow-up. Two studies (Huang R.C. et al., 2017; Lu, 2017) involving 148 participants assessed MoCA at 12-month follow-up. Meta-analysis showed that CMIs significantly improved MoCA scores compared with the control group (MD = 3.05, 95% CI [2.54, 3.57], P < 0.00001; Figure 7), with no evidence of heterogeneity (I^2^ = 0%).

**FIGURE 8:**
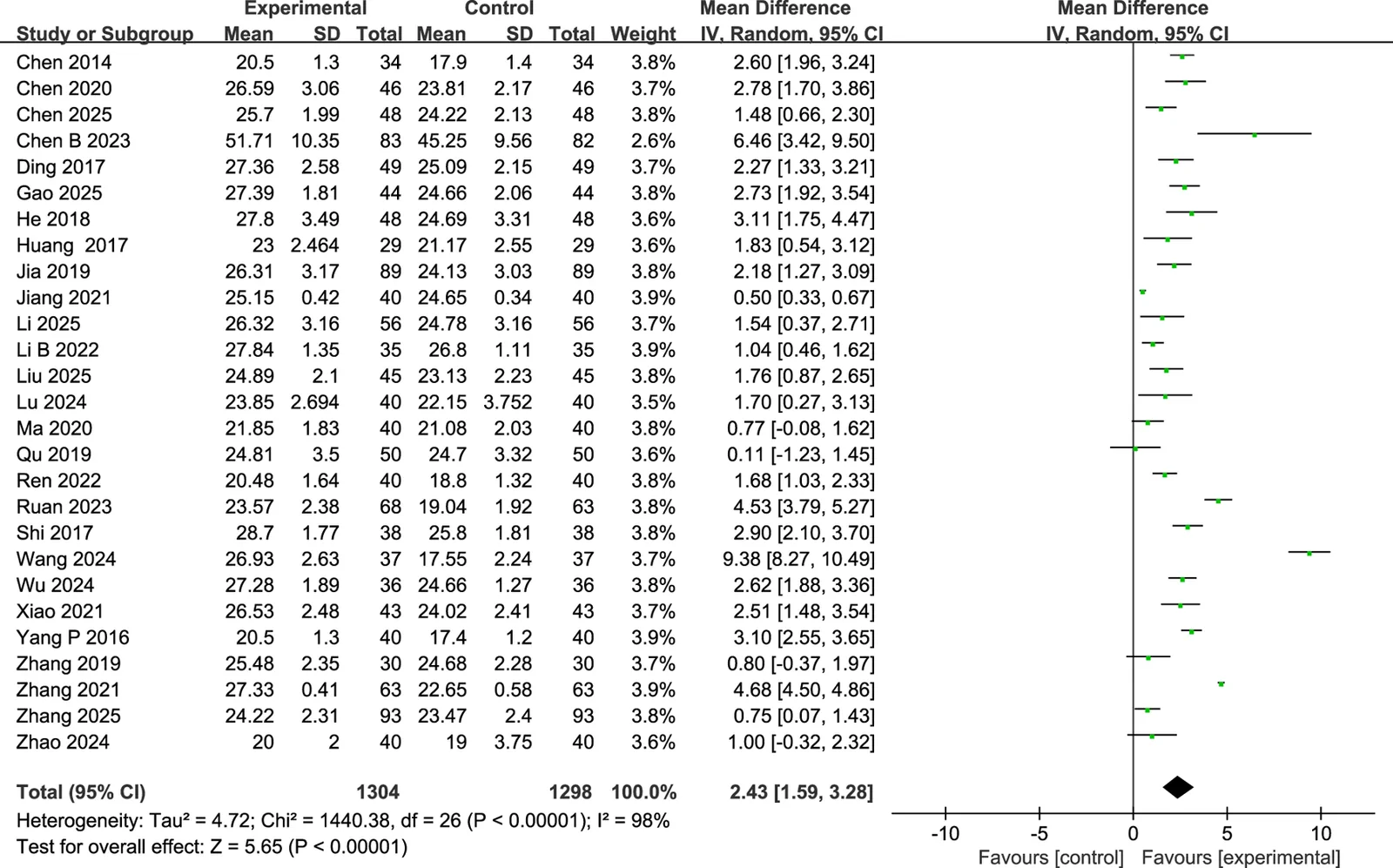
Forest plot of MMSE at the end of treatment for CMIs plus CT versus CT alone in patients with CSVD-CI.

MMSE score at the end of treatment. A total of 27 studies involving 2,602 participants reported short-term MMSE outcomes. The pooled analysis demonstrated that CMIs significantly improved MMSE scores in patients with CSVD-CI compared with the control group (MD = 2.43, 95% CI [1.59, 3.28], P < 0.00001; Figure 8). Considerable heterogeneity was present across studies, with an I^2^ value of 98%, and a random-effects model was therefore applied. Given the considerable heterogeneity, subgroup analyses were also conducted according to treatment duration, whether biomedicine was co-administered, dosage form, and CCPP status. Notably, significant benefits of CMIs were consistently observed across all predefined subgroups. Regarding treatment duration, although the between-subgroup difference was not statistically significant (P = 0.08), heterogeneity was lower in the ≥24 weeks subgroup (I^2^ = 71%), suggesting that treatment duration may have partly contributed to the observed heterogeneity. The corresponding forest plots are provided in Supplementary Figures S5–S8. Notably, none of the interaction P values met the Bonferroni-corrected significance threshold of 0.00625, providing no statistically robust evidence of subgroup differences.

**FIGURE 9:**
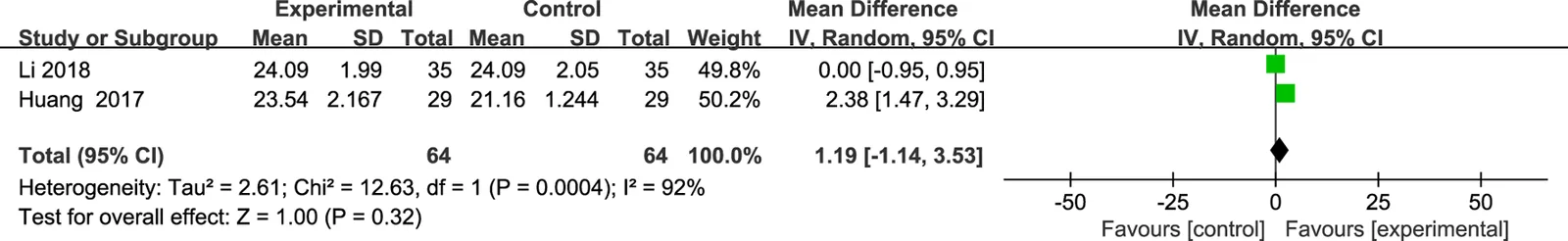
Forest plot of MMSE at 6-month follow-up for CMIs plus CT versus CT alone in patients with CSVD-CI.

MMSE score at 6-month follow-up. Two studies (Huang et al., 2017; Li, 2018) involving 128 participants reported MMSE at 6-month follow-up. Although the pooled estimate favored CMIs, the difference between groups did not reach statistical significance (MD = 1.19, 95% CI [–1.14, 3.53], P = 0.32; Figure 9). Marked heterogeneity was observed across studies (I^2^ = 92%).

**FIGURE 10:**
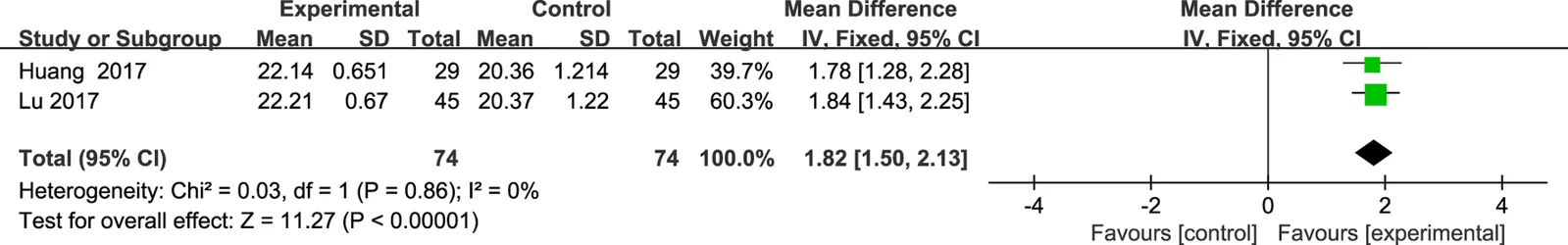
Forest plot of MMSE at 12-month follow-up for CMIs plus CT versus CT alone in patients with CSVD-CI.

MMSE score at 12-month follow-up. Two studies (Huang R.C. et al., 2017; Lu, 2017) involving 148 participants reported MMSE at 12-month follow-up, and the pooled result also favored CMIs (MD = 1.82, 95% CI [1.50, 2.13], P < 0.00001; Figure 10). No heterogeneity was detected across studies (I^2^ = 0%).

**FIGURE 11:**
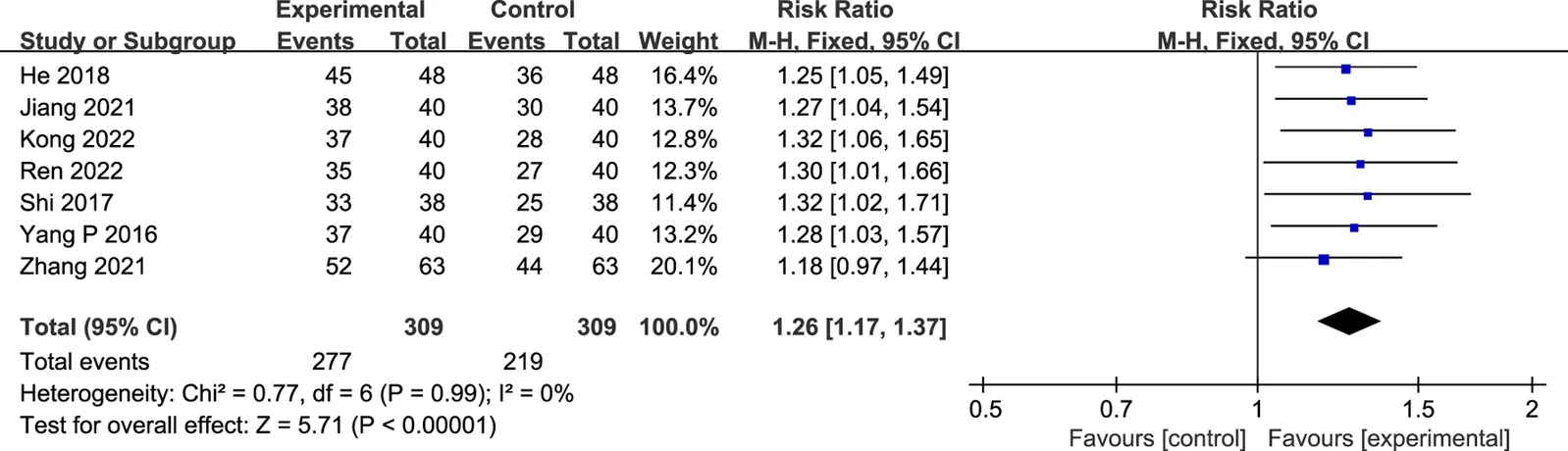
Forest plot of total effective rate for CMIs plus CT versus CT alone in patients with CSVD-CI.

Total effective rate. Total effective rate was reported in 7 studies (He, 2018; Jiang et al., 2021; Kong et al., 2022; Ren et al., 2022; Shi, 2017; Yang et al., 2016; Zhang and Jia, 2021) involving 618 participants. Meta-analysis demonstrated that CMIs significantly increased the total effective rate compared with the control group (RR = 1.26, 95% CI [1.17, 1.37], P < 0.00001; Figure 11). No evidence of heterogeneity was observed across studies, with an I^2^ value of 0%.

**FIGURE 12:**
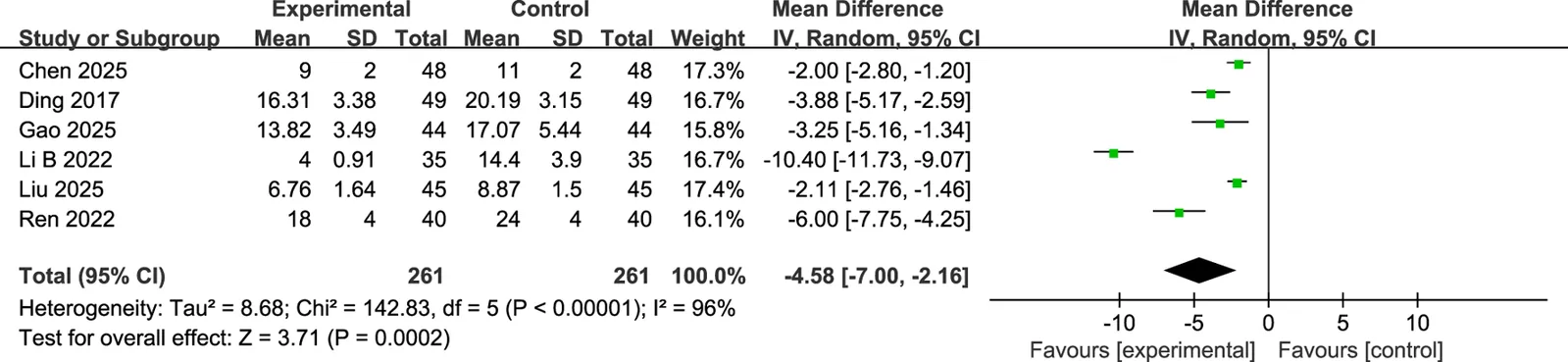
Forest plot of Traditional Chinese Medicine (TCM) syndrome score for CMIs plus CT versus CT alone in patients with CSVD-CI.

Traditional Chinese Medicine (TCM) syndrome score. Traditional Chinese Medicine (TCM) syndrome score was reported in 6 studies (Chen et al., 2025; Ding, 2017; Gao et al., 2025; Li et al., 2022; Liu et al., 2025; Ren et al., 2022) involving 522 participants. Meta-analysis showed that CMIs significantly reduced TCM syndrome scores compared with the control group (MD = −4.58, 95% CI [−7.00, −2.16], P = 0.0002; Figure 12), although heterogeneity remained considerable (I^2^ = 96%).

Biochemical indicators. Pooled analyses of biochemical outcomes generally favored CMIs. Homocysteine (HCY) was reported in 10 studies (Chen K. L. et al., 2023; Chen Y.R. et al., 2020; Guo and Bai, 2019; He, 2018; Jiang et al., 2021; Li L. et al., 2022; Qu, 2019; Ruan et al., 2023; Sun et al., 2023; Zhang et al., 2021) involving 941 participants. Using a random-effects model, the pooled analysis showed that CMIs significantly reduced HCY levels compared with the control group (MD = −4.47, 95% CI [−5.50, −3.45], P < 0.00001; Supplementary Figure S9). Similarly, hs-CRP was reported in 8 (Chen K. L. et al., 2023; Chen Y.R. et al., 2020; Guo and Bai, 2019; Liang et al., 2020; Lu, 2017; Qu, 2019; Ren et al., 2022; Xiao and Huang, 2021) studies involving 679 participants and was significantly lower in the CMI group (MD = −2.05, 95% CI [−2.95, −1.15], P < 0.00001; Supplementary Figure S10). In addition, MDA was assessed in two studies (Xie et al., 2020; Xu et al., 2025) involving 180 participants, and the pooled result also favored CMIs (MD = −0.81, 95% CI [−1.26, −0.36], P = 0.0004; Supplementary Figure S11). However, heterogeneity was substantial for these outcomes.

Hemorheological parameters. Chinese medicine interventions (CMIs) also showed significant benefits for hemorheological parameters. Whole blood viscosity at high shear rate (WBV−H) was reported in 4 studies (Chen et al., 2014; Qu, 2019; Wang, 2024; Wang J. et al., 2023) involving 357 participants and was significantly reduced in the CMI group compared with the control group (MD = −0.80, 95% CI [−1.26, −0.35], P = 0.0005; Supplementary Figure S12). Whole blood viscosity at low shear rate (WBV−L) was likewise reported in 4 studies (Chen et al., 2014; Qu, 2019; Wang, 2024; Wang et al., 2023) involving 357 participants, with a significant reduction in favor of CMIs (MD = −2.13, 95% CI [−3.09, −1.17], P < 0.0001; Supplementary Figure S13). Plasma viscosity (PV) was assessed in three studies (Qu, 2019; Wang, 2024; Wang et al., 2023) involving 289 participants and was also lower in the CMI group than in the control group (MD = −2.95, 95% CI [−5.54, −0.36], P = 0.03; Supplementary Figure S14). Substantial heterogeneity was observed across these analyses.

**FIGURE 13:**
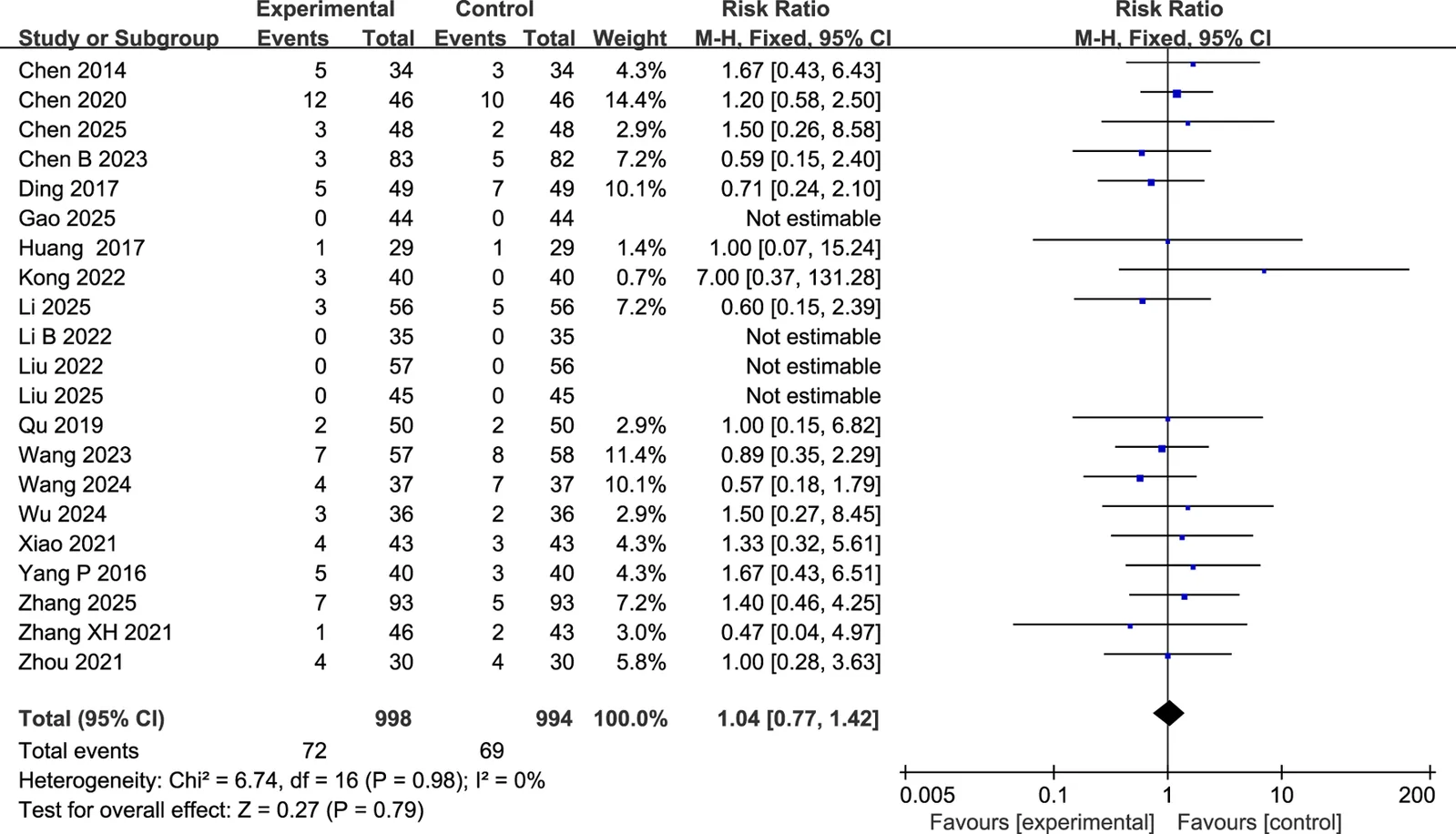
Forest plot of adverse events (AEs) for CMIs plus CT versus CT alone in patients with CSVD-CI.

Adverse events (AEs). Adverse events (AEs) were reported in 21 studies involving 1,992 participants. Meta-analysis indicated that the incidence of AEs was comparable between the CMI and control groups (RR = 1.04, 95% CI [0.77, 1.42], P = 0.79; Figure 13), with no apparent heterogeneity across studies (I^2^ = 0%).

#### 3.4.2 Chinese medicine interventions (CMIs) plus CT versus biomedicine plus CT

MoCA score. Four studies (Gao and Liu, 2023; Huang P.Z. et al., 2017; Li L. et al., 2022; Zhang D.C. et al., 2019) involving 263 participants assessed MoCA outcomes. Although the pooled estimate favored CMIs plus CT, the difference did not reach statistical significance when compared with biomedicine plus CT (MD = 1.47, 95% CI [−0.12, 3.07], P = 0.07; Supplementary Figure S15). Heterogeneity across studies was considerable, with an I^2^ value of 88%.

MMSE score. Four studies (Gao and Liu, 2023; Huang P.Z. et al., 2017; Ji et al., 2016; Li L. et al., 2022) involving 266 participants reported MMSE outcomes, and the pooled analysis showed a significant benefit of CMIs plus CT over biomedicine plus CT (MD = 1.46, 95% CI [0.63, 2.29], P = 0.0006; Supplementary Figure S16). Heterogeneity was moderate (I^2^ = 51%).

#### 3.4.3 Chinese medicine interventions (CMIs) plus CT versus placebo plus CT

MoCA score. Only 1 study (Meng et al., 2020) involving 78 participants was available for this comparison. The reported mean MoCA score was higher in the CMI plus CT group than in the placebo plus CT group, with values of 22.58 and 17.26, respectively.

MMSE score. The same study also reported a higher mean MMSE score in the CMI plus CT group than in the placebo plus CT group, with corresponding values of 29.65 and 25.32.

### 3.5 Sensitivity analysis

Sensitivity analyses for the primary outcomes were performed using a leave-one-out approach. Although exclusion of individual studies reduced heterogeneity to some extent, the direction and statistical significance of the pooled results for MoCA and MMSE did not materially change, indicating that the primary findings were relatively robust.

### 3.6 Meta-regression analyses

Given the substantial heterogeneity observed in the primary outcomes, exploratory meta-regression analyses were performed to further investigate potential study-level moderators of between-study variability. Meta-regression included 35 studies for MoCA and 27 studies for MMSE (Table 1).

**TABLE 1:** Univariable and multivariable meta-regression analyses.

Outcome	Analysis	Moderator	k	Beta	95% CI	P	Residual τ^2^	Residual I^2^ (%)	R^2^ (%)	Overall model P
MMSE	Univariable	Sample size (per 10 participants)	27	0.058	−0.164 to 0.280	0.610	3.36	96.92	0.00	0.610
MMSE	Univariable	Treatment duration (per 4 weeks)	27	−0.207	−0.609 to 0.194	0.312	3.24	97.15	0.00	0.312
MMSE	Univariable	CCPP (yes vs. no)	27	0.526	−0.928 to 1.979	0.478	3.28	97.21	0.00	0.478
MMSE	Multivariable	Sample size (per 10 participants)	27	0.026	−0.201 to 0.252	0.825	3.32	96.32	0.00	0.507
MMSE	Multivariable	Treatment duration (per 4 weeks)	27	−0.289	−0.728 to 0.149	0.196	3.32	96.32	0.00	0.507
MMSE	Multivariable	CCPP (yes vs. no)	27	0.864	−0.731 to 2.458	0.288	3.32	96.32	0.00	0.507
MoCA	Univariable	Sample size (per 10 participants)	35	−0.053	−0.214 to 0.107	0.514	2.15	97.52	0.00	0.514
MoCA	Univariable	Treatment duration (per 4 weeks)	35	0.031	−0.295 to 0.358	0.851	2.18	96.39	0.00	0.851
MoCA	Univariable	CCPP (yes vs. no)	35	0.803	−0.237 to 1.842	0.130	2.03	97.01	3.77	0.130
MoCA	Univariable	Biomedicine background (yes vs. no)	35	−0.393	−1.607 to 0.821	0.526	2.14	96.67	0.00	0.526
MoCA	Multivariable	Sample size (per 10 participants)	35	−0.057	−0.218 to 0.105	0.493	2.14	93.87	0.00	0.481
MoCA	Multivariable	Treatment duration (per 4 weeks)	35	−0.032	−0.366 to 0.301	0.849	2.14	93.87	0.00	0.481
MoCA	Multivariable	CCPP (yes vs. no)	35	0.927	−0.173 to 2.026	0.099	2.14	93.87	0.00	0.481
MoCA	Multivariable	Biomedicine background (yes vs. no)	35	−0.534	−1.795 to 0.728	0.407	2.14	93.87	0.00	0.481

β represents the change in the estimated between-group mean difference associated with a 10-participant increase in sample size or a 4-week increase in treatment duration. For binary moderators, β represents the difference in treatment effect for “yes” versus “no.” k indicates the number of studies included in the model. Residual τ^2^ and residual I^2^ quantify the unexplained between-study heterogeneity after inclusion of the moderator(s), whereas R^2^ represents the proportion of between-study variance explained by the model. The overall model P value represents the omnibus test of all moderators included in the model and is therefore repeated across rows belonging to the same multivariable model. All P values are two-sided, with P < 0.05 considered statistically significant. CCPP, commercial Chinese polyherbal preparation; CI, confidence interval; MMSE, Mini-Mental State Examination; MoCA, montreal cognitive assessment.

For MoCA outcomes, none of the prespecified moderators, including sample size, treatment duration, commercial Chinese polyherbal preparation (CCPP) status, and concomitant biomedicine therapy, showed statistically significant associations with treatment effects in univariable analyses (all P > 0.05). In the multivariable model, none of the examined moderators remained statistically significant, and the overall model was not significant (P = 0.481). The model explained only a small proportion of between-study variance (R^2^ = 0.00%), with substantial residual heterogeneity remaining (residual I^2^ = 93.87%).

For MMSE outcomes, univariable meta-regression analyses similarly showed no significant associations between the prespecified moderators (sample size, treatment duration, and CCPP status) and effect estimates (all P > 0.05). The multivariable model was also not statistically significant (P = 0.507), explaining no appreciable proportion of between-study variance (R^2^ = 0.00%). Substantial residual heterogeneity remained after adjustment (residual I^2^ = 96.32%).

Overall, the exploratory meta-regression analyses did not identify statistically significant study-level moderators that could explain the observed heterogeneity.

### 3.7 Publication bias

**FIGURE 14:**
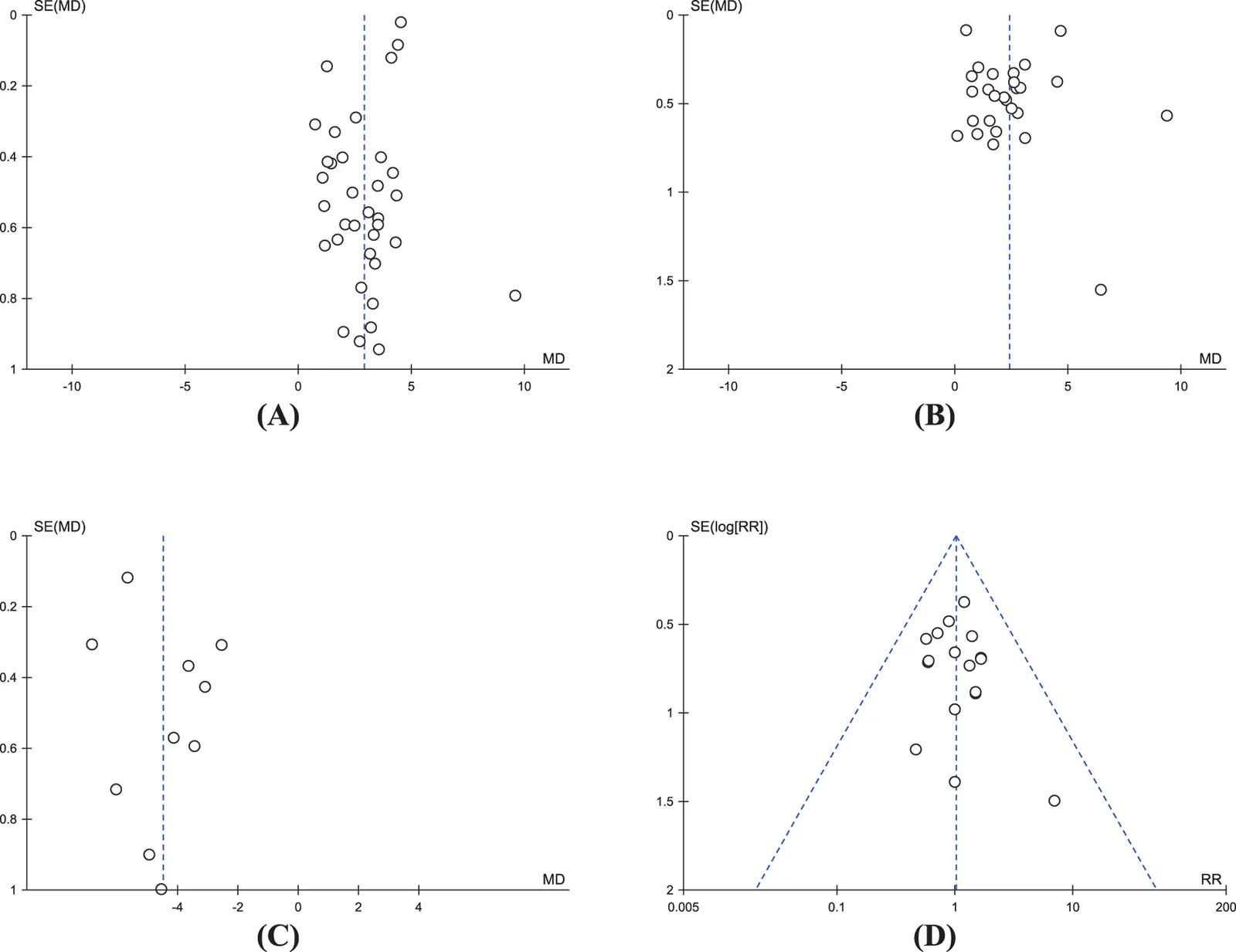
Funnel plots for publication bias assessment in the comparison of CMIs plus CT versus CT alone. **(A)** MoCA at the end of treatment; **(B)** MMSE at the end of treatment; **(C)** homocysteine (HCY); and **(D)** adverse events (AEs).

Publication bias was assessed only for outcomes reported in at least 10 studies, including MoCA at the end of treatment, MMSE at the end of treatment, HCY, and AEs. Visual inspection of the funnel plots for MoCA, MMSE, and AEs did not reveal obvious asymmetry. For MMSE, the funnel plot suggested some asymmetry; however, Egger’s test did not detect significant small-study effects (β1 = 1.61, SE = 1.552, z = 1.04, P = 0.2988). Likewise, Egger’s test did not detect significant small-study effects for HCY (β1 = 0.02, SE = 1.922, z = 0.01, P = 0.9909), AEs (β1 = 0.06, SE = 0.656, z = 0.09, P = 0.9244), or MoCA (β1 = 0.68, SE = 1.148, z = 0.60, P = 0.5514). Overall, there was no statistically significant evidence of small-study effects, although the HCY result should still be interpreted cautiously. The funnel plots are shown in Figure 14, and the corresponding Egger’s regression plots are provided in the Supplementary Material (Supplementary Figure S17).

### 3.8 Certainty of evidence for the comparison of CMIs plus CT versus CT alone

GRADE assessment was performed for the comparison of CMIs plus CT versus CT alone, which provided the most complete evidence base for the evaluated outcomes. For cognitive outcomes assessed at the end of treatment, the certainty of evidence was low for both MoCA and MMSE. The certainty of evidence was moderate for AEs and low for total effective rate. In contrast, the certainty of evidence was very low for HCY, hs-CRP, and TCM syndrome score. Risk of bias and inconsistency were the main reasons for downgrading, whereas imprecision, indirectness, and suspected publication bias also contributed to downgrading in some outcomes. Detailed GRADE assessments are presented in Table 2.

**TABLE 2:** GRADE assessment of the certainty of evidence for included outcomes.

Outcomes	Number of RCTs	GRADE domains					Sample size		Effect estimate	Certainty of the evidence
		Risk of bias	Inconsistency	Indirectness	Imprecision	Publication bias	T	C		
MoCA	35	serious	serious	not serious	not serious	suspected	1,627	1,621	MD 2.92 (95% CI [2.46, 3.39]); I^2^ = 97%	low
MMSE	27	serious	serious	not serious	not serious	suspected	1,304	1,298	MD 2.43 (95% CI [1.59, 3.28]); I^2^ = 98%	low
TCM syndrome score	6	serious	serious	serious	serious	not assessable	261	261	MD -4.58 (95% CI [−7.00, −2.16]); I^2^ = 96%	very low
total effective rate	7	serious	not serious	serious	not serious	not assessable	309	309	RR 1.26 (95% CI [1.17, 1.37]); I^2^ = 0%	low
HCY	10	serious	very serious	not serious	serious	suspected	471	470	MD −4.47 (95% CI [−5.50, −3.45]); I^2^ = 95%	very low
hs-CRP	8	serious	serious	not serious	serious	not assessable	340	339	MD −2.05 (95% CI [−2.95, −1.15]); I^2^ = 94%	very low
AEs	21	serious	not serious	not serious	not serious	not detected	998	994	RR 1.04 (95% CI [0.77, 1.42]); I^2^ = 0%	moderate

Abbreviations: MoCA, montreal cognitive assessment; MMSE, Mini-Mental State Examination; HCY, homocysteine; hs-CRP, high-sensitivity C-reactive protein; CI, confidence interval; MD, mean difference; RR, risk ratio; RCT, randomized controlled trial.

## 4 Discussion

### 4.1 Summary of the findings

This study systematically synthesized evidence from 50 RCTs involving 4,454 patients with CSVD-CI. In the primary comparisons, adding CMIs to an otherwise comparable background treatment was associated with higher end-of-treatment MoCA and MMSE scores. The pooled mean differences were 2.92 points for MoCA and 2.43 points for MMSE, representing absolute between-group differences in endpoint scores. However, the certainty of evidence for both outcomes was low, and the very high between-study heterogeneity means that these values should be interpreted as average estimates across diverse studies rather than as treatment effects expected uniformly across clinical settings. Notably, none of the 50 included trials reported or applied a validated minimum clinically important difference (MCID) threshold for MoCA or MMSE. Moreover, to our knowledge, no MCID has been specifically validated for cognitive impairment related to cerebral small vessel disease; existing MCID estimates were derived primarily from stroke or Alzheimer disease populations (Lindvall et al., 2024; Muir et al., 2024; Wu et al., 2019). Thus, although the pooled differences were statistically significant and numerically comparable to some thresholds reported in other populations, they cannot establish that individual patients achieved clinically meaningful improvement. Findings at 6 and 12 months were based on only two studies at each time point and were not consistent across cognitive measures; they should therefore be considered exploratory and do not establish sustained benefit after treatment. Favorable between-group differences were also observed for total effective rate, TCM syndrome score, homocysteine, hs-CRP, MDA, and several hemorheological parameters, but the low or very low certainty of much of this evidence and the limited standardization of some outcomes restrict clinical and mechanistic interpretation. Adverse events (AEs) were reported in 21 of the 50 RCTs and no statistically significant between-group difference was detected.

Substantial heterogeneity remained in the major outcomes. This meta-analysis evaluated the overall effects of diverse Chinese medicine interventions (CMIs) as a broad intervention class for CSVD-CI, rather than the efficacy of any single formulation. Thus considerable variation existed across studies in prescription composition, compatibility principles, dosage forms, dosages, and quality control, making it difficult to regard these interventions as fully homogeneous. While such variability may partly reflect the individualized nature of treatment with CMIs in real-world practice, it also increases the complexity of evidence synthesis and mechanistic interpretation (Wei et al., 2026). In addition, although all participants were diagnosed with CSVD-CI, patients across studies may have differed in disease duration, severity, imaging burden, comorbid conditions, and TCM syndrome patterns, and these characteristics were often insufficiently reported in the original studies (Rao et al., 2026; Sun et al., 2025). Background treatment was another potential source of heterogeneity. Even among studies that included concomitant biomedicine, the types and dosages of biomedical medications, as well as the components of conventional care, were not fully consistent. Interestingly, although the overall heterogeneity was similarly substantial for MoCA and MMSE, the potential sources of heterogeneity appeared to differ across the subgroup analyses. This may partly reflect the different sets and numbers of studies contributing to the two outcomes, as well as differences in their measurement properties (de Boer et al., 2025; Jia et al., 2021). MoCA assesses executive function, attention, visuospatial ability, and abstraction more extensively and may be more sensitive to mild and domain-specific cognitive deficits associated with cerebral small vessel disease. Consequently, variations in baseline disease severity, cognitive profiles, educational level, and intervention characteristics may produce greater variation in MoCA estimates. In contrast, MMSE places greater emphasis on orientation, language, and memory and may exhibit ceiling effects in participants with relatively mild cognitive impairment, potentially compressing between-study variation. Differences in scale versions, scoring adjustments, test administration, treatment duration, and concomitant biomedical treatments may have further contributed to the differing subgroup patterns. However, because the two analyses included partially different study populations and several subgroups contained relatively few studies, these findings should be interpreted as exploratory and do not establish that MoCA and MMSE reflect genuinely different treatment effects. Exploratory meta-regression also did not identify significant associations between the examined study-level moderators and cognitive effects. Overall, the pooled estimates should be understood as average observed effects under heterogeneous study conditions and not as universally applicable clinical-effect estimates. Overall, the meta-analysis showed statistically significantly higher end-of-treatment scores in the intervention groups than in the control groups, suggesting a potential cognitive benefit of CMIs. However, substantial heterogeneity, low certainty of evidence, and the absence of a validated CSVD-CI-specific MCID limit the clinical interpretation of these findings. Therefore, although the results are encouraging, further high-quality evidence is needed to determine whether the observed differences represent clinically meaningful improvements for individual patients.

### 4.2 Modern medical mechanisms of CSVD-CI

CSVD is increasingly understood not simply as a collection of neuroimaging abnormalities, but as an age-related disorder of the cerebral small vessels that leads to cumulative structural and functional brain injury (Sachdev et al., 2026). It is highly prevalent in older adults and represents a major cause of stroke, cognitive impairment, and dementia worldwide (Hainsworth et al., 2023). In particular, CSVD is responsible for approximately 25% of ischemic strokes in the form of lacunar stroke and is increasingly recognized as an important contributor to vascular cognitive impairment in ageing populations (Elahi et al., 2023). Rather than resulting from a single pathogenic pathway, CSVD-CI appears to arise from the long-term interaction of multiple vascular and parenchymal processes. Age-related vascular stiffening, degenerative remodeling, endothelial injury, and impaired cerebral autoregulation may progressively compromise microvascular perfusion, while blood-brain barrier dysfunction further facilitates the entry of circulating inflammatory mediators and amplifies local oxidative and inflammatory injury (Hannawi, 2023; Jia et al., 2023; Nyúl-Tóth et al., 2024). These alterations are not isolated events; rather, they reinforce one another and collectively disrupt neurovascular unit homeostasis, impair white matter integrity, and contribute to dysfunction of cortico-subcortical cognitive networks. As a consequence, perfusion-sensitive regions such as the white matter and deep gray matter become particularly susceptible to persistent ischemic injury, thereby leading to the characteristic cognitive deficits of CSVD, especially in executive function, attention, and information-processing speed (Jokinen et al., 2024; Tap et al., 2023).

Importantly, this evolving understanding suggests that CSVD-CI should not be viewed as a purely vascular cognitive disorder in the narrow sense. Increasing evidences indicate that chronic microvascular injury may coexist and interact with neurodegenerative changes, thereby further exacerbating disruption of cognitive networks in later life (Arndt et al., 2025; Elahi et al., 2023). This mechanistic complexity may also help explain why current conventional management, although essential for vascular risk reduction and secondary stroke prevention, remains limited in its ability to reverse established cognitive impairment (Bath et al., 2025). Interventions such as blood pressure control, lipid lowering, antiplatelet therapy, and lifestyle modification are clearly important for slowing disease progression and reducing recurrent vascular events, but they do not directly address the full cascade of microcirculatory dysfunction, neurovascular unit injury, inflammatory activation, oxidative stress, and secondary white matter damage that underlies cognitive decline once CSVD-CI has developed (Hainsworth et al., 2023; He and Sun, 2024; Markus and Joutel, 2025). From this perspective, the limited availability of therapies with clearly established cognitive benefits may reflect, at least in part, a mismatch between the multifactorial pathophysiology of CSVD-CI and the relatively narrow therapeutic targets of current conventional strategies. This also provides a plausible rationale for exploring treatment approaches capable of acting on multiple interconnected mechanisms simultaneously.

### 4.3 Traditional Chinese medicine (TCM) perspectives on CSVD-CI

Although there is no exact disease name corresponding to CSVD-CI in TCM, its clinical manifestations, including memory decline, slowed responsiveness, emotional indifference, reduced speech fluency, and gait instability, can generally be categorized under concepts such as “dementia,” “amnesia,” “dullness,” “vertigo,” and “post-stroke dementia.” From the perspective of TCM theory, the development of CSVD-CI in older adults is not attributed to a single excess pathogenic factor; rather, it is more often characterized by a pattern of deficiency in origin and excess in manifestation, with deficiency and excess coexisting. With ageing, kidney essence gradually declines, spleen qi tends to weaken, the generation of qi and blood becomes insufficient, and the brain loses nourishment. As the disease progresses, qi deficiency leads to impaired propulsion, blood flow becomes sluggish, and phlegm turbidity, blood stasis, and toxic injury become intertwined within the brain collaterals, depriving the clear orifices of proper nourishment and ultimately giving rise to multidimensional dysfunction involving cognition, emotion, and gait. Accordingly, the pathogenesis of CSVD-CI in TCM is generally considered to be rooted in deficiency, while stasis, phlegm, and toxin constitute the major excess components. Among these, “qi deficiency and blood stasis” is regarded as a key pathogenic thread throughout disease progression.

When viewed alongside modern pathological concepts, the TCM notions of “deficiency,” “stasis,” “phlegm,” and “toxin” may, at the level of interpretation, correspond to different stages and aspects of CSVD-CI pathogenesis (Bai and Zhang, 2021; Lv et al., 2023). “Deficiency” primarily refers to the underlying state of zang-fu insufficiency, deficiency of qi and blood, and malnourishment of the brain in older individuals, which may be related to reduced cerebral perfusion and increased vulnerability of brain tissue. “Stasis” reflects impaired circulation within the brain collaterals and may correspond, to some extent, to microcirculatory dysfunction and chronic hypoperfusion. “Phlegm” denotes the retention of pathological products and inadequate nourishment of the clear orifices, and may partly parallel brain dysfunction associated with chronic inflammation, metabolic disturbance, and disruption of the internal milieu. “Toxin,” in turn, emphasizes collateral damage and the protracted, refractory nature of disease in its later stages, and may be broadly comparable to processes such as oxidative stress, blood–brain barrier disruption, and secondary brain tissue injury. It should be noted that these correspondences do not imply a mechanical equivalence between the two medical systems; rather, they may help to frame the complex pathogenesis of CSVD-CI from the perspective of whole-body regulation and intervention across multiple pathological processes.

Based on these considerations, the TCM approach to CSVD-CI does not focus solely on the local lesion itself, but instead emphasizes syndrome differentiation and treatment from the perspective of systemic imbalance. Accordingly, Chinese medicine therapy is commonly directed toward strategies such as replenishing qi, tonifying the kidney, promoting blood circulation, resolving phlegm, unblocking the collaterals, and regulating zang-fu function, with the aim of restoring internal balance and thereby alleviating clinical symptoms and functional impairment. For older patients with CSVD-CI, who often have a prolonged disease course, intertwined pathogenic mechanisms, and multisystem degeneration, this holistic therapeutic framework also provides a traditional medical rationale for understanding the potential role of CMIs in disease management.

### 4.4 Therapeutic rationale for CMIs

From the perspective of therapeutic strategy, the potential value of CMIs in the management of CSVD-CI lies not only in their capacity to ameliorate clinical symptoms, but also in the relatively comprehensive therapeutic system it has developed on the basis of syndrome differentiation and treatment. Given the prolonged course, complex pathogenesis, and coexistence of deficiency and excess in this disorder, TCM treatment is not confined to a single lesion site or an isolated pathological process; rather, it emphasizes holistic intervention through approaches such as supplementing deficiency and strengthening vital qi, promoting blood circulation and unblocking the collaterals, resolving phlegm and eliminating turbidity, and regulating zang-fu function, with prescriptions adjusted dynamically according to disease stage and syndrome evolution. The medicinal-material frequency analysis in the present study showed that Chuanxiong Rhizoma, Angelicae Sinensis Radix, Paeoniae Radix Rubra, Spatholobi Caulis, and Pheretima were among the most frequently used medicinal materials. To some extent, this prescription pattern reflects the fundamental therapeutic characteristics of CMIs in CSVD-CI, namely, the combined emphasis on reinforcing the healthy qi while eliminating pathogenic factors, and on balancing tonification with unblocking actions.

Existing experimental studies suggest that the pharmacological activities of these frequently used medicinal materials correspond well to the key pathological changes involved in CSVD-CI. Specifically, Chuanxiong Rhizoma has been associated mainly with improvements in hemorheology, promotion of microcirculation, and attenuation of neuroinflammation (Huang et al., 2024; Zuo et al., 2023). Angelicae Sinensis Radix may exert cerebroprotective effects through anti-apoptotic and neuroprotective actions, as well as through modulation of inflammation and oxidative stress (Lee et al., 2021). Spatholobi Caulis may confer protective effects by suppressing inflammatory cascade reactions, alleviating oxidative injury, and modulating the neuroimmune microenvironment (Ban et al., 2023). Bioactive constituents derived from Pheretima have also demonstrated certain advantages in promoting fibrinolysis, improving perfusion, and regulating inflammatory responses (Wiyarta et al., 2025; Zhu et al., 2024). In addition, several included studies used Ginkgo-related preparations, and existing evidence also suggests their potential roles in improving cerebral blood flow, reducing oxidative injury, and protecting the neurovascular unit (Birks and Grimley Evans, 2009; Chen Y. et al., 2020).

Taken together, current pharmacological evidence provides a degree of biological plausibility for the therapeutic rationale of CMIs in CSVD-CI and offers a hypothesis-generating basis for future studies evaluating their potential clinical efficacy.

### 4.5 Strengths, limitations, and future directions

This study has several notable strengths. First, to our knowledge, it represents one of the most systematic and comprehensive evidence syntheses to date on CMIs for CSVD-CI. Beyond cognitive outcomes, it also incorporated safety outcomes, inflammation- and oxidative stress-related biomarkers, and hemorheological parameters, thereby allowing the effects of the intervention to be evaluated not merely in terms of changes in a single rating scale, but across a broader range of clinically and biologically relevant domains. Second, the present study extended beyond the question of whether CMIs are effective by incorporating prescription frequency analysis, through which several representative frequently used medicinal materials were identified. This approach helps to distill common therapeutic principles and prescription patterns currently used in clinical practice for CSVD-CI, while also providing potentially valuable leads for the identification of frequently co-used medicinal-material pairs, optimization of multi-medicinal-material formulations, and development of candidate therapeutics. Third, short-term outcomes were distinguished from long-term follow-up outcomes, providing a preliminary view of the potential benefits of Chinese medicine interventions across different time horizons on the basis of the currently available evidence. This distinction is of particular relevance for understanding the possible role of CMIs in the long-term management of CSVD-CI.

Nevertheless, several important limitations should be acknowledged. First, nearly all included studies were conducted in China, and most were single-center trials with relatively small sample sizes, and the applicability of the results to populations with different ethnic backgrounds, healthcare systems, dietary patterns, genetic characteristics, and standards of care is uncertain. Although the search imposed no language restrictions and covered four international and four Chinese databases, the predominance of Chinese-language publications means that language-related selection factors cannot be excluded. Second, although random sequence generation was mentioned in many studies, reporting of allocation concealment, implementation of blinding, blinding of outcome assessment, and protocol registration or public availability of study protocols was generally insufficient, which substantially reduced the overall certainty of the evidence. Third, substantial heterogeneity was observed across multiple analyses. This heterogeneity may be partly attributable to differences in participant eligibility criteria, diagnostic approaches, CSVD imaging burden, severity of cognitive impairment, concomitant medications, treatment duration, dosage forms, and timing of outcome assessment. In particular, the diagnostic criteria were not uniform, and the trials did not provide sufficiently detailed group-level MRI data to confirm standardized STRIVE-based phenotyping or distinguish relatively pure CSVD-CI from mixed cognitive impairment. Intervention-related variability may have further contributed to the heterogeneity, as the formulations differed in composition, preparation, dosage form, dose, quality control, and treatment duration, precluding reliable dose-response analyses, comparisons of individual formulations, or network meta-analysis. Moreover, although background treatment was administered equally to both groups within each trial, between-study differences in the specific drugs, doses, treatment intensity, and adherence may have modified the incremental effects of CMIs. Fourth, long-term follow-up data remain relatively limited, and further evidence is still needed to determine whether CMIs can delay disease progression and sustain long-term functional benefits. It should also be noted that, although outcomes such as total effective rate and TCM syndrome score may have certain practical value in local clinical settings, their degree of standardization and international comparability remains limited. Finally, because all included RCTs reported positive findings, unpublished studies with negative or null results may have been missed, and publication bias cannot be excluded.

Future research should place greater emphasis on the intrinsic heterogeneity of CSVD and its implications for trial design. As highlighted by the FINESSE framework, clinical trials in CSVD require more rigorous planning with respect to population phenotyping, clinical endpoint selection, application of neuroimaging surrogate markers, biomarker integration, and overall study design. For randomized controlled trials of CMIs in particular, future studies should prioritize clearly defined MRI-based diagnostic criteria and stratification according to CSVD burden, while making greater efforts to distinguish pure CSVD-CI from mixed cognitive impairment. Establishing and validating a CSVD-CI-specific MCID should also be an important research priority. Core outcomes oriented toward clinically meaningful patient benefit should be prespecified, and integrated assessments combining cognitive scales, MRI markers, and circulating biomarkers should be undertaken. At the same time, greater attention should be paid to standardizing treatment duration and follow-up windows, improving the reporting of concomitant therapies, and adopting multicenter, adequately powered, prospectively registered designs with standardized safety reporting, so as to further enhance the reliability, comparability, and clinical interpretability of the evidence.

## 5 Conclusion

Current low-certainty evidence suggests that, when added to otherwise comparable background treatment, CMIs may be associated with higher end-of-treatment MoCA and MMSE scores and favorable differences in several secondary outcomes. These pooled estimates are average effects across highly heterogeneous studies and do not demonstrate that individual patients experienced clinically meaningful improvement. Because of unclear risk of bias, substantial unexplained heterogeneity, variable formulations and background treatments, possible publication and language bias, limited follow-up, restricted generalizability, and incomplete safety reporting, the evidence does not establish sustained cognitive benefit, specific biological mechanisms, long-term safety, or the superiority of any formulation. The absence of a statistically significant difference in reported adverse events should not be interpreted as proof of equivalence or safety. Rigorous, adequately powered, multicenter RCTs with standardized diagnosis and intervention reporting, clinically meaningful outcomes, longer follow-up, and prospective safety monitoring—including systematic evaluation of potential interactions with commonly prescribed cardiovascular medications—are needed before CMIs can be recommended with confidence for CSVD-CI. Complementary pharmacokinetic and pharmacodynamic studies should clarify interactions between specific CMI preparations and concomitant medications.

## Data Availability

The original contributions presented in the study are included in the article/Supplementary Material, further inquiries can be directed to the corresponding authors.
