# Supplementary material for: Chinese medicine interventions for cerebral small vessel disease-related cognitive impairment: a systematic review and meta-analysis
Source: Front Pharmacol. 2026 Aug 28;17:1894066. doi: 10.3389/fphar.2026.1894066 (PMC13564464; doi:10.3389/fphar.2026.1894066)
Supplement: Supplementary file 1 [file Table1.docx]

Supplementary Appendix

Appendix A. Search strategy

Appendix B. Supplementary figure legends

Appendix C. Supplementary tables

1. Appendix A. Search strategy

For retrieval of databases, the keywords of search are the same, with taking pubmed as an example:

#1 "Cerebral Small Vessel Diseases"[MeSH Terms] OR "Leukoencephalopathies"[MeSH Terms] OR "Leukoaraiosis"[MeSH Terms] OR "White Matter"[MeSH Terms] OR ("white matter hyperintensity*"[Title/Abstract] OR "WMHs"[Title/Abstract] OR "cerebral microbleed*"[Title/Abstract] OR "brain microbleed*"[Title/Abstract] OR "CMB"[Title/Abstract] OR "cerebral microinfarct*"[Title/Abstract] OR "perivascular space*"[Title/Abstract] OR "PVS"[Title/Abstract] OR "lacunar infarction"[Title/Abstract] OR "lacunar infarct*"[Title/Abstract] OR "brain atrophy"[Title/Abstract] OR "CSVD"[Title/Abstract]

#2 "Cognitive Dysfunction"[MeSH Terms] OR "Memory Disorders"[MeSH Terms] OR ("cognitive impairment"[Title/Abstract] OR "cognitive decline"[Title/Abstract] OR "cognitive disorder*"[Title/Abstract] OR "memory disorder*"[Title/Abstract] OR "memory impairment"[Title/Abstract] OR "mental deterioration"[Title/Abstract]

#3 "drugs, chinese herbal"[MeSH Terms] OR "medicine, chinese traditional"[MeSH Terms] OR "Chinese herbal medicine"[Title/Abstract] OR "traditional Chinese medicine"[Title/Abstract] OR "herbal medicine"[Title/Abstract] OR "Chinese patent medicine"[Title/Abstract] OR "compound prescription"[Title/Abstract] OR "Chinese medicine preparation"[Title/Abstract] OR "herbal injection"[Title/Abstract] OR "injection"[Title/Abstract] OR "oral liquid"[Title/Abstract] OR "capsule"[Title/Abstract] OR "granule"[Title/Abstract] OR "decoction"[Title/Abstract] OR "herbal pieces"[Title/Abstract] OR "TCM"[Title/Abstract] OR "Phytotherapy"[MeSH Terms] OR "medicine, traditional"[MeSH Terms] OR "Plant Extracts"[MeSH Terms] OR "plants, medicinal"[MeSH Terms] OR "herbal drug"[Title/Abstract] OR "phytomedicine"[Title/Abstract] OR "phytotherapeutic*"[Title/Abstract] OR "plant medicine"[Title/Abstract] OR "natural medicine*"[Title/Abstract] OR "natural drug*"[Title/Abstract] OR "medicinal plant*"[Title/Abstract] OR "plant extract*"[Title/Abstract] OR "herbal extract*"[Title/Abstract] OR "herbal therap*"[Title/Abstract] OR "plant therap*"[Title/Abstract] OR "traditional medicine"[Title/Abstract]

#4 #1 AND #2 AND #3

## 2. Appendix B. Supplementary figure legends

Figure S1. Forest plot of subgroup analysis for MoCA at the end of treatment according to treatment duration in the comparison of Chinese medicine interventions (CMIs) plus CT versus CT alone.

Figure S2. Forest plot of subgroup analysis for MoCA at the end of treatment according to whether biomedicine was used in the comparison of CMIs plus CT versus CT alone.

Figure S3. Forest plot of subgroup analysis for MoCA at the end of treatment according to dosage form in the comparison of CMIs plus CT versus CT alone.

Figure S4. Forest plot of subgroup analysis for MoCA at the end of treatment according to commercial Chinese polyherbal preparation (CCPP) status in the comparison of CMIs plus CT versus CT alone.

Figure S5. Forest plot of subgroup analysis for MMSE at the end of treatment according to treatment duration in the comparison of CMIs plus CT versus CT alone.

Figure S6. Forest plot of subgroup analysis for MMSE at the end of treatment according to whether biomedicine was used in the comparison of CMIs plus CT versus CT alone.

Figure S7. Forest plot of subgroup analysis for MMSE at the end of treatment according to dosage form in the comparison of CMIs plus CT versus CT alone.

Figure S8. Forest plot of subgroup analysis for MMSE at the end of treatment according to CCPP status in the comparison of CMIs plus CT versus CT alone.

Figure S9. Forest plot of HCY for CMIs plus CT versus CT alone in patients with CSVD-CI.

Figure S10. Forest plot of hs-CRP for CMIs plus CT versus CT alone in patients with CSVD-CI.

Figure S11. Forest plot of MDA for CMIs plus CT versus CT alone in patients with CSVD-CI.

Figure S12. Forest plot of WBV-H for CMIs plus CT versus CT alone in patients with CSVD-CI.

Figure S13. Forest plot of WBV-L for CMIs plus CT versus CT alone in patients with CSVD-CI.

Figure S14. Forest plot of PV for CMIs plus CT versus CT alone in patients with CSVD-CI.

Figure S15. Forest plot of MoCA for CMIs plus CT versus biomedicine plus CT in patients with CSVD-CI.

Figure S16. Forest plot of MMSE for CMIs plus CT versus biomedicine plus CT in patients with CSVD-CI.

Figure S17. Egger’s regression plots for publication bias assessment in the comparison of CMIs plus CT versus CT alone. (A) MoCA at the end of treatment; (B) MMSE at the end of treatment; (C) homocysteine (HCY); and (D) adverse events (AEs).

## 3. Appendix C. Supplementary tables

**Table S1. Characteristics of the included studies**

| **Study ID** | **Country** | **Diagnostic criteria** | **Sample size** | | **Sex (Male/ Female)** | | **Mean age (years)** | | **Intervention** | | **Outcomes** | **Adverse events reported（T/C）** |
| --- | --- | --- | --- | --- | --- | --- | --- | --- | --- | --- | --- | --- |
|  |  |  | **T** | **C** | **T** | **C** | **T** | **C** | **T** | **C** |  |  |
| Chen 2014 (Chen N. et al., 2014) | China | Draft Diagnostic Criteria for Vascular Dementia (2002) | 34 | 34 | 15/19 | 16/18 | 61.5 ± 11.2 | 62.2 ± 12.7 | Tongxinluo Capsule + CT + Cattle Encephalon Glycoside + Ignotin + Edaravone Injection | CT + Cattle Encephalon Glycoside + Ignotin + Edaravone Injection | ②⑦⑧⑪ | Gastric pain, 3; Nausea, 2/ Nausea and gastric discomfort, 3 |
| Chen 2019 (Chen S.N. et al., 2019) | China | Spectrum of disease in vascular cognitive impairment (1999) | 30 | 30 | 16/14 | 14/16 | 67.53 ± 6.06 | 69.83 ± 4.80 | Huayu Tongluo Decoction + CT | CT | ① | NR |
| Chen 2020 (Chen Y.R. et al., 2020) | China | Chinese Consensus on Diagnosis and Treatment of Cerebral Small Vessel Disease (2015) | 46 | 46 | 26/20 | 25/21 | 57.09 ± 6.42 | 56.16 ± 6.82 | Naokang Granules + CT + Nimodipine Tablets | CT + Nimodipine Tablets | ②⑤⑥⑪ | Abnormal liver function, 3; Gastrointestinal reactions, 2; Renal impairment, 3; Dizziness, 4/ Abnormal liver function, 2; Gastrointestinal reactions, 2; Rash, 1; Renal impairment, 2; Dizziness, 3 |
| Chen 2023 (Chen K.L. et al., 2023) | China | Chinese Clinical Practice Guideline for Cognitive Impairment Related to Cerebral Small Vessel Disease (2019) | 30 | 30 | 15/15 | 14/16 | 63.18 ± 3.99 | 63.54 ± 4.21 | Buyang Huanwu Decoction + CT | CT | ①⑤⑥ | NR |
| Chen 2025 (Chen S.Y. et al., 2025) | China | Guidelines for Diagnosis and Treatment of Vascular Cognitive Impairment (2011) | 48 | 48 | 27/21 | 26/22 | 64. 11 ± 5. 98 | 63. 89 ± 6. 07 | Bushen Yizhi Formula Decoction + CT + Donepezil Tablets | CT + Donepezil Tablets | ①②④⑪ | Nausea, 1; Dizziness, 2/ Diarrhea, 1; Nausea, 1 |
| Chen B 2023 (Chen B. et al., 2023) | China | Expert Consensus on Diagnosis and Treatment of Cerebral Small Vessel Disease (2014) + Spectrum of diseasein vascular cognitive impairment (1999) | 83 | 82 | 48/35 | 49/33 | 61.2 ± 6.3 | 60.8 ± 6.5 | Huayu Tongluo Decoction + CT | CT | ①②⑪ | Nausea and vomiting, 1; Dry mouth, 2/ Nausea and vomiting, 2; Dry mouth, 2; Constipation, 1 |
| Ding 2017 (Ding, 2017) | China | Guidelines for Diagnosis and Treatment of Vascular Cognitive Impairment (2011) | 49 | 49 | 29/20 | 27/22 | 62.66 ± 8.31 | 62.85 ± 8.22 | Fufang Congrong Yizhi Capsule + CT + Oxiracetam Capsules | CT + Oxiracetam Capsules | ①④⑪ | Palpitations, 1; Nausea, 2; Abdominal pain and diarrhea, 3; Nervous excitation, 1/ Palpitations, 1; Nausea, 1; Abdominal pain and diarrhea, 1; Nervous excitation, 2 |
| Gao 2023 (Gao and Liu, 2023) | China | Chinese Clinical Practice Guideline for Cognitive Impairment Related to Cerebral Small Vessel Disease (2019) | 30 | 30 | 18/12 | 15/15 | 61.77 ± 10.25 | 66.47 ± 10.54 | Naoxinkang Decoction + CT | CT + Idebenone Tablets | ①②③ | NR |
| Gao 2025 (Gao et al., 2025) | China | Chinese Guidelines for Diagnosis and Treatment of Cognitive Dysfunction associated with Small cerebral Vascular Disease (2019) | 44 | 44 | 19/25 | 22/22 | 1.99 ± 1.54 | 2.13 ± 1.52 | Kongsheng Zhenzhong Dan Decoction + CT + Idebenone Tablets | CT + Idebenone Tablets | ①②④⑪ | No adverse events/ No adverse events |
| Guo 2019 (Guo and Bai, 2019) | China | Chinese Consensus on Diagnosis and Treatment of Cerebral Small Vessel Disease (2015) | 48 | 47 | 27/21 | 28/19 | 62.29 ± 9.30 | 63.73 ± 10.81 | Yangxue Qingnao Granules + CT + Donepezil Tablets | CT + Donepezil Tablets | ①⑤⑥ | NR |
| He 2018 (He, 2018) | China | Guidelines for the diagnosis and treatment of cerebral small vessel diseases (2013) | 48 | 48 | 27/21 | 29/19 | 65.79 ± 7.91 | 65.27 ± 7.67 | Ginkgo Biloba Leaf Capsule + CT + Nimodipine Tablets | CT + Nimodipine Tablets | ①②③⑤ | NR |
| Huang 2017 (Huang R.C. et al., 2017) | China | Expert Consensus on Diagnosis and Treatment of Cerebral Small Vessel Disease (2014) + Spectrum of diseasein vascular cognitive impairment (1999) | 29 | 29 | 12/17 | 15/14 | 70.48 ± 8.971 | 69.90 ± 9.155 | Huayu Tongluo Decoction + CT | CT | ①② | Nausea and vomiting, 1/ Dry mouth and constipation, 1 |
| Huang 2019 (Huang H.Y. et al., 2019) | China | NR | 45 | 45 | 30/15 | 29/16 | 62.15 ± 5.71 | 60.89 ± 7.34 | Yangxue Qingnao Granules + CT + biomedicine | CT + biomedicine | ① | NR |
| Huang PZ 2017 (Huang P.Z. et al., 2017) | China | Expert Recommendations on Diagnosis and Treatment Process of Cognitive Impairment in Chinese Elderly (2014) | 38 | 38 | 20/18 | 21/17 | 63.36 ± 3.52 | 64.15 ± 3.82 | Yishen Tongluo Huazhuo Decoction + CT | CT + Aniracetam Tablets | ①②③ | NR |
| Ji 2016 (Ji et al., 2016) | China | Mild cognitive impairment (MCI) in medical practice: a critical review of the concept and new diagnostic procedure. Report of the MCI Working Group of the European Consortium on Alzheimer's Disease(2006) + Mild cognitive impairment – beyond controversies, towards a consensus: report of the International Working Group on Mild Cognitive Impairment (2004) + Neuropsychological predictors of incident dementia in patients with vascular cognitive impairment, without dementia (2002) | 30 | 30 | - | - | - | - | Qingnao Yizhi Granules + CT | CT + Nimodipine Tablets | ②④⑩⑪ | No adverse events/ No adverse events |
| Jia 2019 (Jia, 2019) | China | Guidelines for Diagnosis and Treatment of Vascular Cognitive Impairment | 89 | 89 | 53/36 | 55/34 | 73.2 ± 12.4 | 72.7 ± 12.2 | Naokang Granules + CT + Nimodipine Tablets | CT + Nimodipine Tablets | ①② | NR |
| Jiang 2021 (Jiang et al., 2021) | China | Chinese Clinical Practice Guideline for Cognitive Impairment Related to Cerebral Small Vessel Disease (2019) | 40 | 40 | 22/18 | 27/13 | 68.18 ± 5.76 | 69.08 ± 4.92 | Wanshi Qingxin Huatan Granules + CT + Nimodipine Tablets | CT + Nimodipine Tablets | ①②③⑤ | NR |
| Kong 2022 (Kong et al., 2022) | China | Chinese Guidelines for Diagnosis and Treatment of Cognitive Dysfunction associated with Small cerebral Vascular Disease (2019) | 40 | 40 | 26/14 | 20/20 | 67.08 ± 10.24 | 65.68 ± 10.39 | Yangxue Qingnao Granules + CT + Donepezil Tablets | CT + Donepezil Tablets | ①③⑪ | Nausea, 1/ Nausea, 0 |
| Li 2018 (Li, 2018) | China | Expert Consensus on Diagnosis and Treatment of Cerebral Small Vessel Disease (2014) | 35 | 35 | 18/17 | 19/16 | 62.5 ± 3.2 | 62.2 ± 3.4 | Huayu Tongluo Decoction + CT | CT | ②③⑪ | Adverse events, 1/ Adverse events, 3 |
| Li 2025 (Li M.J. et al., 2025) | China | Chinese Clinical Practice Guideline for Cognitive Impairment Related to Cerebral Small Vessel Disease (2019) | 56 | 56 | 33/23 | 35/21 | 73. 27 ± 7. 80 | 73. 96 ± 7. 27 | Yangxue Qingnao Granules + CT + Citicoline Sodium Tablets | CT + Citicoline Sodium Tablets | ①②⑪ | Nausea, 1; Pruritus, 1; Gastric discomfort, 1; Diarrhea, 1; Dry mouth, 1/ Nausea, 1; Gastric discomfort, 1; Dry mouth, 1 |
| Li B 2022 (Li L. et al., 2022) | China | Chinese Expert Consensus on the Treatment of Cerebral Small Vessel Disease (2021) | 35 | 35 | Group 1: 17/18 Group 2: 18/17 | 17/18 | - | - | Group 1: Fufang Congrong Yizhi Capsule + CT；Group 2: Fufang Congrong Yizhi Capsule + CT + Nicergoline Tablets | CT + Nicergoline Tablets | ①②④⑤⑪ | No adverse events/ No adverse events |
| Liang 2020 (Liang et al., 2020) | China | Chinese Consensus on Diagnosis and Treatment of Cerebral Small Vessel Disease (2015) | 43 | 43 | 20/23 | 22/21 | 68.51 ± 6.01 | 69.05 ± 6.13 | Yangxue Qingnao Granules + CT + Donepezil Tablets | CT + Donepezil Tablets | ①⑥ |  |
| Liu 2022 (Liu H.S. et al., 2022) | China | Chinese Guidelines for Diagnosis and Treatment of Cognitive Dysfunction associated with Small cerebral Vascular Disease (2019) | 59 | 59 | 26/33 | 27/32 | 61. 4 ± 8. 3 | 61. 2 ± 8. 4 | Nao An Capsule + Nimodipine Tablets | Nimodipine Tablets | ①⑪ | No adverse events/ No adverse events |
| Liu 2025 (Liu X.F. et al., 2025) | China | Chinese Guidelines for Diagnosis and Treatment of Cognitive Dysfunction associated with Small cerebral Vascular Disease (2019) | 45 | 45 | 26/19 | 23/22 | 63.53 ± 8.50 | 62.38 ± 9.37 | Shenwu Yizhi Capsule + CT + Donepezil Tablets | CT + Donepezil Tablets | ①②④⑪ | No adverse events/ No adverse events |
| Lu 2017 (Lu, 2017) | China | Expert Consensus on Diagnosis and Treatment of Cerebral Small Vessel Disease (2014) | 45 | 45 | 25/15 | 26/14 | 64. 14 ± 5. 01 | 64. 41 ± 5. 17 | Chaiqin Wendan Decoction + CT | CT | ①② | NR |
| Lu 2024 (Lu C.G. et al., 2024) | China | Chinese Clinical Practice Guideline for Cognitive Impairment Related to Cerebral Small Vessel Disease (2019) | 40 | 40 | 23/17 | 20/20 | 54.98 ± 8.47 | 55.88 ± 8.51 | Decoction + Donepezil Tablets + HBOT | Donepezil Tablets + HBOT | ①②⑥ | NR |
| Ma 2020 (Ma et al., 2020) | China | Chinese Clinical Practice Guideline for Cognitive Impairment Related to Cerebral Small Vessel Disease (2019) | 40 | 40 | 25/15 | 21/19 | 47~78 | 45~80 | Yangxue Qingnao Granules + CT + Donepezil Tablets | CT + Donepezil Tablets | ① | NR |
| Meng 2020 (Meng et al., 2020) | China | Expert Consensus on Diagnosis and Treatment of Cerebral Small Vessel Disease (2014) | 39 | 39 | 22/17 | 21/18 | 64.35 ± 6.86 | 63.74 ± 6.57 | Wenxiao III Formula Granules + CT | placebo + CT | ①②⑪ | NR |
| Qu 2019 (Qu, 2019) | China | Guidelines for the diagnosis and treatment of cerebral small vessel diseases(2013) + Guidelines for diagnosis and treatment of vascular cognitive impairment (2011) | 50 | 50 | 27/23 | 28/22 | 66.13 ± 4.63 | 65.37 ± 4.49 | Ginkgo Biloba Leaf Capsule + CT | CT | ①②⑤⑥⑦⑧⑨⑪ | Headache, 1; Decreased blood pressure, 1/ Facial flushing, 1; Decreased blood pressure, 1 |
| Qu 2020 (Qu et al., 2020) | China | NR | 47 | 47 | 28/19 | 27/20 | 63.06 ± 5.28 | 62.95 ± 5.39 | Ginkgo Biloba Leaf Capsule + CT + Mecobalamin Tablets | CT + Mecobalamin Tablets | ①⑤ | NR |
| Ren 2022 (Ren et al., 2022) | China | Chinese Consensus on Diagnosis and Treatment of Cerebral Small Vessel Disease (2015) | 40 | 40 | 23/17 | 21/19 | 58.66 ± 5.68 | 57.18 ± 6.06 | Peiyuan Kaizhi Decoction + CT | CT | ②③④⑥ | No adverse events/ No adverse events |
| Ruan 2023 (Ruan et al., 2023) | China | Chinese Clinical Practice Guideline for Cognitive Impairment Related to Cerebral Small Vessel Disease (2019) | 63 | 68 | 36/32 | 33/30 | 58.42 ± 14.13 | 58.37 ± 14.21 | Yangxue Qingnao Granules + CT + Donepezil Tablets | CT + Donepezil Tablets | ①②⑤ | NR |
| Shi 2017 (Shi, 2017) | China | NR | 38 | 38 | 20/18 | 24/14 | 69.2 ± 6.4 | 71.1 ± 5.3 | Ginkgo Biloba Leaf Tablet + CT + Nimodipine Tablets | CT + Nimodipine Tablets | ①②③ | NR |
| Sun 2023 (Sun X.J. et al., 2023) | China | Chinese Guidelines for Diagnosis and Treatment of Cognitive Dysfunction associated with Small cerebral Vascular Disease (2019) | 48 | 48 | 25/23 | 26/22 | 63.45 ± 8.30 | 63.41 ± 8.27 | Fufang Congrong Yizhi Capsule + CT + Donepezil Tablets | CT + Donepezil Tablets | ①⑤ | NR |
| Wang 2023 (Wang J. et al., 2023) | China | Chinese Guidelines for Diagnosis and Treatment of Cognitive Dysfunction associated with Small cerebral Vascular Disease (2019) | 57 | 58 | 33/24 | 31/27 | 62.46 ± 4.20 | 63.66 ± 3.90 | Tongqiao Huoxue Decoction + CT | CT | ①⑦⑧⑨⑪ | Abdominal pain and diarrhea, 5; Nausea and vomiting, 2/ Abdominal pain and diarrhea, 4; Nausea and vomiting, 4 |
| Wang 2024 (Wang, 2024) | China | Chinese Consensus on Diagnosis and Treatment of Cerebral Small Vessel Disease (2015) | 37 | 37 | 22/15 | 20/17 | 62.27 ± 3.36 | 62.15 ± 3.42 | Ginkgo Biloba Leaf Capsule + CT + Mecobalamin Tablets | CT + Mecobalamin Tablets | ①②⑦⑧⑨⑪ | Hypotension, 1; Gastrointestinal discomfort, 2; Headache, 1/ Hypotension, 3; Gastrointestinal discomfort, 2; Skin tingling, 1; Headache, 1 |
| Wu 2024 (Wu et al., 2024) | China | Chinese Consensus on Diagnosis and Therapy of Cerebral Small Vessel Disease (2021) | 36 | 36 | 20/16 | 19/17 | 62.13 ± 6.28 | 61.95 ± 6.15 | Peiyuan Tongnao Capsule + CT | CT | ①②⑪ | Nausea and vomiting, 1; Dizziness, 1; Rash, 1/ Nausea and vomiting, 1; Rash, 1 |
| Xiao 2021 (Xiao and Huang, 2021) | China | Chinese Consensus on Diagnosis and Treatment of Cerebral Small Vessel Disease (2015) | 43 | 43 | 26/17 | 24/19 | 62.17 ± 4.65 | 62.14 ± 4.62 | Ginkgo Biloba Extract + CT + Donepezil | CT + Donepezil | ①②⑥⑪ | Headache, 1; Vomiting, 2; Diarrhea, 1/ Headache, 1; Vomiting, 1; Diarrhea, 1 |
| Xie 2020 (Xie et al., 2020) | China | NR | 32 | 32 | 19/13 | 17/15 | 68. 23 ± 2. 15 | 67. 82 ± 1.97 | Yangxue Qingnao Pills + CT + Donepezil Tablets | CT + Donepezil Tablets | ①⑩ | NR |
| Xu 2025 (Xu et al., 2025) | China | Chinese Consensus on Diagnosis and Therapy of Cerebral Small Vessel Disease (2021) | 58 | 58 | 30/28 | 32/36 | 65.56 ± 5.84 | 66.24 ± 6.13 | Zhishe Tongluo Capsule + CT | CT | ①②⑩ | Dizziness, 3; Nausea, 4/ Dizziness, 3; Nausea, 4; Vomiting, 1 |
| Yang P 2016 (Yang P. et al., 2016) | China | Mild cognitive impairment: clinical characterization and outcome (1999) | 40 | 40 | 15/25 | 17/23 | 61.5 ± 14.2 | 62.2 ± 12.7 | Tongxinluo Capsule + CT | CT | ①③⑪ | Gastric pain and nausea, 5/ Gastric discomfort and nausea, 3 |
| Yang 2016 (Yang and Ge, 2016) | China | Mild cognitive impairment: clinical characterization and outcome (1999) | 24 | 24 | 16/8 | 13/11 | 66 ± 4 | 64 ± 5 | Yangxue Qingnao Granules + CT | CT | ① | NR |
| Zhang SX 2019 (Zhang S.X. et al., 2019) | China | Chinese Consensus on Diagnosis and Treatment of Cerebral Small Vessel Disease (2015) | 30 | 30 | 16/14 | 20/10 | 62. 2 ± 8. 4 | 61. 7 ± 8. 1 | Jiawei Dihuang Yin Decoction + CT + Oxiracetam Capsules | CT + Oxiracetam Capsules | ①② | NR |
| Zhang 2021 (Zhang and Jia, 2021) | China | Chinese Guidelines for Diagnosis and Treatment of Cognitive Dysfunction associated with Small cerebral Vascular Disease (2019) | 63 | 63 | 35/28 | 33/30 | 77.3 ± 2.1 | 79.6 ± 2.5 | Tongqiao Huoxue Decoction + CT + Nimodipine Tablets + Donepezil Tablets | CT + Nimodipine Tablets + Donepezil Tablets | ①②③⑤ | NR |
| Zhang 2025 (Zhang F.P. et al., 2025) | China | Chinese guideline for diagnosis and treatment of cerebral small vessel disease (2020) | 93 | 93 | 53/40 | 49/44 | 73.71 ± 7.05 | 72.05 ± 7.19 | Fufang Huonaoshu Capsule + CT + Citicoline Sodium Tablets | CT + Citicoline Sodium Tablets | ①②⑪ | Anorexia, 2; Nausea, 2; Gastric discomfort, 2; Dizziness, 1/ Anorexia, 1; Nausea, 1; Dizziness, 1; Gastric discomfort, 2 |
| Zhang DC 2019 (Zhang D.C. et al., 2019) | China | Chinese Consensus on Diagnosis and Treatment of Cerebral Small Vessel Disease (2015) | 30 | 30 | 21/9 | 18/12 | 63.15 ± 8.32 | 61.34 ± 9.87 | Zhilong Huoxue Capsule + CT | CT + Nimodipine | ①⑤ | No adverse events/ No adverse events |
| Zhang XH 2021 (Zhang X.H. et al., 2021) | China | Chinese Guidelines for Diagnosis and Treatment of Cognitive Dysfunction associated with Small cerebral Vascular Disease (2019) | 45 | 43 | 32/14 | 25/18 | 55. 2 ± 4. 5 | 54. 6 ± 4. 3 | Kaiqiao Tongluo Decoction + CT + Citicoline Sodium + Edaravone Injection | CT + Citicoline Sodium + Edaravone Injection | ①⑪ | Nausea and vomiting, 1/ Dizziness and elevated blood pressure, 2 |
| Zhao 2024 (Zhao, 2024) | China | Chinese Consensus on Diagnosis and Therapy of Cerebral Small Vessel Disease (2021) + Chinese guideline for diagnosis and treatment of cerebral small vessel disease (2020) | 40 | 40 | 21/19 | 20/20 | 74 | 75 | Shenrong Tongmai Capsule + CT | CT | ①② | NR |
| Zhou 2019 (Zhou H. et al., 2019) | China | Vascular dementia: diagnostic criteria for research studies. Report of the NINDS-AIREN International Workshop | 42 | 46 | 24/18 | 26/20 | 68.7 ± 8.18 | 66.7 ± 10.8 | Tongxinluo Capsule + CT + NBP Tablets | CT + NBP Tablets | ① | NR |
| Zhou 2021 (Zhou, 2021) | China | Chinese Consensus on Diagnosis and Treatment of Cerebral Small Vessel Disease(2015) | 30 | 30 | 16/14 | 18/12 | 64.83 ± 7.40 | 65.25 ± 6.37 | Yangxue Qingnao Granules + CT + NBP Tablets | CT + NBP Tablets | ①⑪ | Dizziness, 1; Rash, 1; Gastrointestinal discomfort, 2 / Dizziness, 2; Gastrointestinal discomfort, 1; Insomnia, 1 |

Note: T, treatment group; C, control group; CT, conventional therapy; M, male; F, female; HBOT, hyperbaric oxygen therapy; NBP, dl-3-n-butylphthalide; NR, not reported. Treatment duration is reported in days, weeks, or months, as described in the original studies. Outcomes are coded as follows: ① Montreal Cognitive Assessment (MoCA); ② Mini-Mental State Examination (MMSE); ③ total effective rate; ④ Traditional Chinese Medicine (TCM) syndrome score; ⑤ homocysteine (HCY); ⑥ high-sensitivity C-reactive protein (hs-CRP); ⑦ whole blood viscosity at high shear rate (WBV-H); ⑧ whole blood viscosity at low shear rate (WBV-L); ⑨ plasma viscosity (PV); ⑩ malondialdehyde (MDA); ⑪ adverse events (AEs). For the study by Li B (2022) that included 2 intervention groups, Group 1 refers to the CMI plus CT group, and Group 2 refers to the CMI plus CT plus biomedicine group.

## Table S2. Treatment characteristics of intervention and control groups

| **Study ID** | **Intervention groups** | | | | **Control groups (name; frequency; dosage form; route of administration)** |
| --- | --- | --- | --- | --- | --- |
|  | **Chinese medicine intervention / formula name (volume per intake, frequency; dosage form; route of administration)** | **Species name (family, plant part), daily dosage** | **Indication; adverse reactions; contraindications; source; quality control reported (Y/NR).** | **Description of the extract and extraction process** |  |
| Chen 2014 | Tongxinluo Capsule (0.78g, tid; capsule, oral) | *Panax ginseng* C.A.Mey. [Araliaceae; Ginseng Radix et Rhizoma], *Whitmania pigra* Whitman [Hirudinidae; Hirudo], *Buthus martensii* Karsch [Buthidae; Scorpio], *Paeonia veitchii* Lynch. [Ranunculaceae; Paeoniae Radix Rubra], *Cryptotympana pustulata* Fabricius [Cicadidae; Cicadae Periostracum], *Eupolyphaga sinensis* Walker [Corydiidae; Eupolyphaga seu Steleophaga], *Scolopendra subspinipes* mutilans L. Koch [Scolopendridae; Scolopendra], *Santalum album* L. [Santalaceae; Santali Albi Lignum], *Dalbergia odorifera* T.Chen [Fabaceae; Dalbergiae Odoriferae Lignum], *Boswellia carterii* Birdw. [Burseraceae; Olibanum], *Ziziphus jujuba* Mill. var. spinosa (Bunge) Hu ex H.F.Chou [Rhamnaceae; Ziziphi Spinosae Semen], *Dryobalanops aromatica* C. F. Gaertn. [Dipterocarpaceae; Borneolum Syntheticum] | Indication: Invigorating qi and promoting blood circulation, dredging collaterals and relieving pain. Used for coronary heart disease and angina pectoris with heart qi deficiency and blood stasis obstructing collaterals, manifested as chest oppression, stabbing pain, colicky pain, fixed pain, palpitations, spontaneous sweating, shortness of breath and fatigue, dark purple tongue or ecchymosis, fine and rough or intermittent pulse. Also used for stroke of qi deficiency and blood stasis obstructing collaterals type, manifested as hemiplegia or unilateral numbness, deviated mouth and tongue, slurred speech, Adverse reactions: Occasional gastrointestinal discomfort such as nausea, vomiting, abdominal pain, abdominal distension, diarrhea, and stomach discomfort, as well as rash, itching, dizziness, etc., Contraindications: Hemorrhagic diseases, pregnant women, women during menstruation, and yin deficiency with excessive fire type stroke are contraindicated; Shijiazhuang Yiling Pharmaceutical Co., Ltd.; Z19980015 | Grind Scorpion, Leech, Centipede, Ground Beetle, and Cicada Slough into ultrafine powder below 10 μm. Distill Sandalwood and Dalbergia odorifera with water vapor to collect volatile oil; the residue is then boiled in water, filtered, and concentrated under vacuum. Soak Ginseng in 70% ethanol, reflux-extract, recover the ethanol, and concentrate under vacuum; the residue is then boiled in water, filtered, and concentrated under vacuum. Boil Sour Jujube Seed and Red Peony Root in water, filter, and concentrate under vacuum. Combine all concentrated extracts, mix thoroughly with the ultrafine powder, spray-dry, and grind. Add an appropriate amount of excipient to form granules. Incorporate the volatile oil from Sandalwood and Dalbergia odorifera, mix well with Borneol, and encapsulate to obtain the final product. | Control groups (Cattle Encephalon Glycoside + Ignotin + Edaravone; Injection; ivgtt) |
| Chen S.N 2019 | Huayu Tongluo Decoction (100ml, qd; granule; oral) | *Ilex* *pubescens* Hook. & Arn. [Aquifoliaceae; Ilicis Pubescentis Radix] 30g, *Angelica* *sinensis* (Oliv.) Diels [Apiaceae; Angelicae Sinensis Radix] 15g, *Ligusticum* *chuanxiong* Hort. [Apiaceae; Chuanxiong Rhizoma] 10g, *Paeonia veitchii Lynch*. [Ranunculaceae; Paeoniae Radix Rubra] 10g, *Pheretima* *aspergillum* (E. Perrier) [Megascolecidae; Pheretima] 10g, *Moschus* [Cervidae; Moschus Artifactus]0.1g | NR | NR | ct |
| Chen 2020 | Naokang Granule (NR, bid; granule; oral) | *Epimedium* *brevicornu* Maxim. [Berberidaceae; Epimedii Folium] 15g, *Cistanche* *deserticola* Y.C.Ma [Orobanchaceae; Cistanches Herba]15g, *Astragalus* *membranaceus* (Fisch.) Bunge [Fabaceae; Astragali Radix]20g, *Codonopsis* *pilosula* (Franch.) Nannf. [Campanulaceae; Codonopsis Radix] 15g, *Buthus* *martensii* Karsch [Buthidae; Scorpio], *Ligusticum* *chuanxiong* Hort. [Apiaceae; Chuanxiong Rhizoma] 10g, *Salvia* *miltiorrhiza* Bunge [Lamiaceae; Salviae Miltiorrhizae Radix et Rhizoma] 10g, *Acorus* *tatarinowii* Schott [Acoraceae; Acori Tatarinowii Rhizoma]10g, *Pheretima* *aspergillum* (E. Perrier) [Megascolecidae; Pheretima] 6g, *Bombyx* *mori* Linnaeus [Bombycidae; Bombyx Batryticatus] 6g | NR | NR | Control groups (Nimodipine; tid; tablet; oral) |
| Chen K.L 2023 | Buyang Huanwu Decoction (200ml, bid; decoction; oral) | *Astragalus* *membranaceus* (Fisch.) Bunge [Fabaceae; Astragali Radix] 30g, *Carthamus* *tinctorius* L. [Asteraceae; Carthami Flos] 3g, *Ligusticum* *chuanxiong* Hort. [Apiaceae; Chuanxiong Rhizoma] 9g, *Prunus* *persica* (L.) Batsch [Rosaceae; Persicae Semen] 10g, *Angelica* *sinensis* (Oliv.) Diels [Apiaceae; Angelicae Sinensis Radix] 9g, *Paeonia veitchii* Lynch. [Ranunculaceae; Paeoniae Radix Rubra] 10g, *Pheretima* *aspergillum* (E. Perrier) [Megascolecidae; Pheretima] 10g, *Whitmania pigra* Whitman [Hirudinidae; Hirudo] 10g | NR | NR | Control groups (Donepezil; qd; tablet; oral) |
| Chen S.Y 2025 | Bushen Yizhi Formula Decoction (NR, bid; granule; oral) | *Panax* *ginseng* C.A.Mey. [Araliaceae; Ginseng Radix et Rhizoma] 6g, *Codonopsis* *pilosula* (Franch.) Nannf. [Campanulaceae; Codonopsis Radix] 20g, *Astragalus* *membranaceus* (Fisch.) Bunge [Fabaceae; Astragali Radix] 30g, *Rehmannia* *glutinosa* (Gaertn.) Libosch. ex Fisch. et Mey. [Orobanchaceae; Rehmanniae Radix Praeparata] 20g, *Polygonatum* *sibiricum* Red. [Asparagaceae; Polygonati Rhizoma] 12g, *Cuscuta* *chinensis* Lam. [Convolvulaceae; Cuscutae Semen] 9g, *Cistanche* *deserticola* Y.C.Ma [Orobanchaceae; Cistanches Herba] 12g, *Angelica* *sinensis* (Oliv.) Diels [Apiaceae; Angelicae Sinensis Radix] 12g, *Ligusticum* *chuanxiong* Hort. [Apiaceae; Chuanxiong Rhizoma] 9g, *Carthamus* *tinctorius* L. [Asteraceae; Carthami Flos] 12g, *Prunus* *persica* (L.) Batsch [Rosaceae; Persicae Semen] 12g, *Polygala* *tenuifolia* Willd. [Polygalaceae; Polygalae Radix] 15g, *Acorus* *tatarinowii* Schott [Acoraceae; Acori Tatarinowii Rhizoma] 9g, *Dryobalanops* *aromatica* C. F. Gaertn. [Dipterocarpaceae; Borneolum Syntheticum] 3g | NR | NR | Control groups (Donepezil; qd; tablet; oral) |
| Chen B 2023 | Huayu Tongluo Mixture (100ml, tid; granule; oral) | *Ilex* *pubescens* Hook. & Arn. [Aquifoliaceae; Ilicis Pubescentis Radix] 30g, *Angelica* *sinensis* (Oliv.) Diels [Apiaceae; Angelicae Sinensis Radix] 15g, *Ligusticum* *chuanxiong* Hort. [Apiaceae; Chuanxiong Rhizoma] 10g, *Paeonia veitchii Lynch*. [Ranunculaceae; Paeoniae Radix Rubra] 10g, *Pheretima* *aspergillum* (E. Perrier) [Megascolecidae; Pheretima] 10g, *Artificial Musk* [-; Moschus Artifactus] 0.1g | NR | NR | ct |
| Ding 2017 | Fufang Congrong Yizhi Capsule (4caps, tid; capsule; oral) | *(Cistanche* *deserticola* Y.C.Ma [Orobanchaceae; Cistanches Herba], *Polygonum* *multiflorum* Thunb. [Polygonaceae; Polygoni Multiflori Radix Praeparata], *Pheretima* *aspergillum* (E. Perrier) [Megascolecidae; Pheretima], *Nelumbo* *nucifera* Gaertn. [Nelumbonaceae; Nelumbinis Folium], *Rhaponticum* *uniflorum* (L.) DC. [Asteraceae; Rhapontici Radix]) NA | NR | Grind Polygonum multiflorum into coarse powder. Reflux-extract twice with 75% ethanol, first with 8-fold volume for 1 hour, second with 6-fold volume for 1 hour. Combine the ethanol extracts and concentrate to a clear extract of relative density 1.28–1.30 at 60°C. Boil Nelumbo nucifera leaf, Cistanche deserticola, and Rhaponticum uniflorum in water twice, first with 12-fold volume for 2 hours, second with 10-fold volume for 1 hour. Combine the decoction, filter, and concentrate to relative density 1.04–1.05 at 60°C. Cool, add ethanol to 60% alcohol content, refrigerate for 12 hours, filter, and concentrate the filtrate to a clear extract of relative density 1.28–1.30 at 60°C. Combine with the above extract and dry under vacuum below 80°C. Grind Pheretima into coarse powder, homogenize with 4-fold volume of water 5 times for 15 minutes each. Combine the homogenate, add ethanol to 10% alcohol content, centrifuge, then add ethanol to the supernatant to 51% alcohol content, centrifuge again. Wash the precipitate with acetone twice, centrifuge, and dry the precipitate under vacuum. Combine with the above dry extract, add an appropriate amount of dextrin, grind into medium powder, and encapsulate to obtain the final product. | Control groups (Oxiracetam; bid; capsule; oral) |
| Gao 2023 | Naoxinkang Decoction (200ml, bid; granule; oral) | *Angelica* *sinensis* (Oliv.) Diels [Apiaceae; Angelicae Sinensis Radix] 15g, *Astragalus* *membranaceus* (Fisch.) Bunge [Fabaceae; Astragali Radix] 30g, *Panax* *notoginseng* (Burkill) F.H.Chen [Araliaceae; Notoginseng Radix et Rhizoma] 3g, *Eupolyphaga* *sinensis* Walker [Corydiidae; Eupolyphaga seu Steleophaga] 12g, *Prunus* *persica* (L.) Batsch [Rosaceae; Persicae Semen] 15g, *Carthamus* *tinctorius* L. [Asteraceae; Carthami Flos] 15g, *Ligusticum* *chuanxiong* Hort. [Apiaceae; Chuanxiong Rhizoma] 15g, *Paeonia veitchii Lynch*. [Ranunculaceae; Paeoniae Radix Rubra] 15g, *Pheretima* *aspergillum* (E. Perrier) [Megascolecidae; Pheretima] 30g, *Buthus* *martensii* Karsch [Buthidae; Scorpio] 9g, *Pueraria* *lobata* (Willd.) Ohwi [Fabaceae; Puerariae Lobatae Radix] 20g, *Spatholobus* *suberectus* Dunn [Fabaceae; Spatholobi Caulis] 30g, *Zaocys* *dhumnades* (Cantor) [Colubridae; Zaocys] 15g, *Aspongopus* *chinensis* Dallas [Pentatomidae; Aspongopus] 12g, *Bombyx* *mori* Linnaeus [Bombycidae; Bombyx Batryticatus] 15g, *Vaccaria* *segetalis* (Neck.) Garcke [Caryophyllaceae; Vaccariae Semen] 15g, *Achyranthes* *bidentata* Blume [Amaranthaceae; Achyranthis Bidentatae Radix] 12g, *Taxillus* *chinensis* (DC.) Danser [Loranthaceae; Taxilli Herba] 15g, *Drynaria* *fortunei* (Kunze) J.Sm. [Polypodiaceae; Drynariae Rhizoma] 15g, *Cyperus* *rotundus* L. [Cyperaceae; Cyperi Rhizoma] 15g, *Citrus* *aurantium* L. [Rutaceae; Aurantii Fructus] 15g, *Citrus* *reticulata* Blanco [Rutaceae; Citri Reticulatae Pericarpium Viride] 15g, *Citrus* *reticulata* Blanco [Rutaceae; Citri Reticulatae Pericarpium] 15g | NR | NR | Control groups (Idebenone; tid; tablet; oral) |
| Gao 2025 | Kongsheng Zhenzhong Dan Decoction (400ml, bid; granule; oral) | *Trionyx* *sinensis* Wiegmann [Trionychidae; Trionycis Carapax] 30g, *Os Draconis* [-; Os Draconis] 30g, *Acorus* *tatarinowii* Schott [Acoraceae; Acori Tatarinowii Rhizoma] 30g, *Polygala* *tenuifolia* Willd. [Polygalaceae; Polygalae Radix] 10g, *Arisaema* *erubescens* (Wall.) Schott [Araceae; Arisaematis Rhizoma] 10g, *Paeonia veitchii Lynch*. [Ranunculaceae; Paeoniae Radix Rubra] 15g, *Ligusticum* *chuanxiong* Hort. [Apiaceae; Chuanxiong Rhizoma] 15g, *Bombyx* *mori* Linnaeus [Bombycidae; Bombyx Batryticatus] 12g, *Prunus* *persica* (L.) Batsch [Rosaceae; Persicae Semen] 9g | NR | NR | Control groups (Idebenone; tid; tablet; oral) |
| Guo 2019 | Yangxue Qingnao Granule (4g, tid; granule; oral) | *(Panax ginseng* C.A.Mey. [Araliaceae; Ginseng Radix et Rhizoma], *Whitmania pigra* Whitman [Hirudinidae; Hirudo], *Buthus martensii* Karsch [Buthidae; Scorpio], *Paeonia veitchii* Lynch. [Ranunculaceae; Paeoniae Radix Rubra], *Cryptotympana pustulata* Fabricius [Cicadidae; Cicadae Periostracum], *Eupolyphaga sinensis* Walker [Corydiidae; Eupolyphaga seu Steleophaga], *Scolopendra subspinipes* mutilans L. Koch [Scolopendridae; Scolopendra], *Santalum album* L. [Santalaceae; Santali Albi Lignum], *Dalbergia odorifera* T.Chen [Fabaceae; Dalbergiae Odoriferae Lignum], *Boswellia carterii* Birdw. [Burseraceae; Olibanum], *Ziziphus jujuba* Mill. var. spinosa (Bunge) Hu ex H.F.Chou [Rhamnaceae; Ziziphi Spinosae Semen], *Dryobalanops aromatica* C. F. Gaertn. [Dipterocarpaceae; Borneolum Syntheticum]) NA | Indication: Nourishing blood and calming the liver, promoting blood circulation and dredging collaterals. Used for headache, dizziness and blurred vision, irritability and anger, insomnia and dreaminess caused by blood deficiency and liver hyperactivity; Adverse reactions: Post-marketing monitoring data show that this product may cause the following adverse reactions: rash, itching, nausea, vomiting, abdominal distension, diarrhea, abdominal pain, gastric burning sensation, dry mouth, dizziness, headache, head distension, tinnitus, palpitations, blood pressure reduction, abnormal liver biochemical indicators, etc.; Contraindications: Pregnant women are contraindicated; patients with decompensated liver function are contraindicated; those allergic to this product and its ingredients are contraindicated; Tasly Pharmaceutical Group Co., Ltd.; Z10960082 | Grind Angelica sinensis, Ligusticum chuanxiong, Paeonia lactiflora, Rehmannia glutinosa, Uncaria rhynchophylla, Spatholobus suberectus, Prunella vulgaris, Cassia obtusifolia, and Corydalis yanhusuo into coarse powder. Decoct Concha margaritifera and Asarum heterotropoides with water twice, first with 10-fold volume for 2 hours, second with 8-fold volume for 1.5 hours. Combine the decoction, filter, and concentrate under vacuum to relative density 1.25–1.30 at 60°C. Add the above coarse powder to the concentrated extract, mix thoroughly, and granulate with an appropriate amount of dextrin and stevioside. Dry at low temperature, sift, and package to obtain the final product. | Control groups (Donepezil; qd; tablet; oral) |
| He 2018 | Gingko Biloba Leaf capsules (2caps, bid; capsule; oral) | *Ginkgo* *biloba* L. [Ginkgoaceae; Ginkgo Folium] NA | Indication: Promoting blood circulation, resolving stasis and dredging collaterals. Used for chest bi and heart pain, stroke, hemiplegia, stiff tongue and slurred speech caused by blood stasis obstructing collaterals; coronary heart disease with stable angina pectoris and cerebral infarction with the above symptoms; Adverse reactions: Post-marketing adverse reaction monitoring data show that ginkgo leaf oral preparations may cause the following adverse reactions: gastrointestinal system (nausea, vomiting, dry mouth, abdominal distension, abdominal pain, abdominal discomfort, excessive gastric acid, etc., with case reports of gastrointestinal bleeding); nervous system (dizziness, headache, etc., with case reports of local numbness); skin and appendages (rash, itching, etc.); cardiovascular system (chest tightness, palpitations, etc., with case reports of blood pressure elevation or reduction); others (fatigue, allergic or allergic-like reactions, etc., with case reports of gum bleeding and nosebleed); Contraindications: Those allergic to this product and its ingredients are contraindicated; Hangzhou Conba Pharmaceutical Co., Ltd.; Z20063069 | Extract Ginkgo biloba leaves with 60–70% ethanol by percolation or reflux, concentrate the ethanol extract under vacuum, and suspend in water. Remove impurities by petroleum ether extraction, then extract with ethyl acetate. Concentrate the ethyl acetate extract and dissolve in acetone for crystallization. Dissolve the purified Ginkgo leaf extract in an appropriate amount of ethanol, mix uniformly with medicinal starch as filler, granulate with 95% ethanol as wetting agent, dry, and sift. Fill the granules into hard capsules, with each capsule containing 9.6 mg of total flavonol glycosides and 2.4 mg of terpene lactones, to obtain the final product. | Control groups (Nimodipine; tid; tablet; oral) |
| Huang 2017 | Huayu Tongluo Decoction (NR, NR; granule; oral) | *Ilex* *pubescens* Hook. & Arn. [Aquifoliaceae; Ilicis Pubescentis Radix] 15g, *Angelica* *sinensis* (Oliv.) Diels [Apiaceae; Angelicae Sinensis Radix] 12g, *Ligusticum* *chuanxiong* Hort. [Apiaceae; Chuanxiong Rhizoma] 12g, *Paeonia veitchii Lynch*. [Ranunculaceae; Paeoniae Radix Rubra] 12g, *Pheretima* *aspergillum* (E. Perrier) [Megascolecidae; Pheretima] 10g, *Bombyx* *mori* Linnaeus [Bombycidae; Bombyx Batryticatus] 10g, *Buthus* *martensii* Karsch [Buthidae; Scorpio] 5g | NR | NR | ct |
| Huang H.Y 2019 | Yangxue Qingnao Granule (4g, tid; granule; oral) | *(Panax ginseng* C.A.Mey. [Araliaceae; Ginseng Radix et Rhizoma], *Whitmania pigra* Whitman [Hirudinidae; Hirudo], *Buthus martensii* Karsch [Buthidae; Scorpio], *Paeonia veitchii* Lynch. [Ranunculaceae; Paeoniae Radix Rubra], *Cryptotympana pustulata* Fabricius [Cicadidae; Cicadae Periostracum], *Eupolyphaga sinensis* Walker [Corydiidae; Eupolyphaga seu Steleophaga], *Scolopendra subspinipes* mutilans L. Koch [Scolopendridae; Scolopendra], *Santalum album* L. [Santalaceae; Santali Albi Lignum], *Dalbergia odorifera* T.Chen [Fabaceae; Dalbergiae Odoriferae Lignum], *Boswellia carterii* Birdw. [Burseraceae; Olibanum], *Ziziphus jujuba* Mill. var. spinosa (Bunge) Hu ex H.F.Chou [Rhamnaceae; Ziziphi Spinosae Semen], *Dryobalanops aromatica* C. F. Gaertn. [Dipterocarpaceae; Borneolum Syntheticum]) NA | Indication: Nourishing blood and calming the liver, promoting blood circulation and dredging collaterals. Used for headache, dizziness and blurred vision, irritability and anger, insomnia and dreaminess caused by blood deficiency and liver hyperactivity; Adverse reactions: Post-marketing monitoring data show that this product may cause the following adverse reactions: rash, itching, nausea, vomiting, abdominal distension, diarrhea, abdominal pain, gastric burning sensation, dry mouth, dizziness, headache, head distension, tinnitus, palpitations, blood pressure reduction, abnormal liver biochemical indicators, etc.; Contraindications: Pregnant women are contraindicated; patients with decompensated liver function are contraindicated; those allergic to this product and its ingredients are contraindicated; Tasly Pharmaceutical Group Co., Ltd.; Z10960082 | Grind Angelica sinensis, Ligusticum chuanxiong, Paeonia lactiflora, Rehmannia glutinosa, Uncaria rhynchophylla, Spatholobus suberectus, Prunella vulgaris, Cassia obtusifolia, and Corydalis yanhusuo into coarse powder. Decoct Concha margaritifera and Asarum heterotropoides with water twice, first with 10-fold volume for 2 hours, second with 8-fold volume for 1.5 hours. Combine the decoction, filter, and concentrate under vacuum to relative density 1.25–1.30 at 60°C. Add the above coarse powder to the concentrated extract, mix thoroughly, and granulate with an appropriate amount of dextrin and stevioside. Dry at low temperature, sift, and package to obtain the final product. | ct |
| Huang PZ 2017 | Yishen Tongluo Huazhuo Decoction (200ml, bid; decoction; oral) | *(Rehmannia* *glutinosa* (Gaertn.) Libosch. ex Fisch. et Mey. [Orobanchaceae; Rehmanniae Radix Recens], *Dioscorea* *oppositifolia* L. [Dioscoreaceae; Dioscoreae Rhizoma], *Cornus* *officinalis* Siebold & Zucc. [Cornaceae; Corni Fructus], *Cistanche* *deserticola* Y.C.Ma [Orobanchaceae; Cistanches Herba], *Ligustrum* *lucidum* W.T.Aiton [Oleaceae; Ligustri Lucidi Fructus], *Ligusticum* *chuanxiong* Hort. [Apiaceae; Chuanxiong Rhizoma], *Paeonia veitchii Lynch*. [Ranunculaceae; Paeoniae Radix Rubra], *Pheretima* *aspergillum* (E. Perrier) [Megascolecidae; Pheretima], *Acorus* *tatarinowii* Schott [Acoraceae; Acori Tatarinowii Rhizoma], *Citrus* *reticulata* Blanco [Rutaceae; Citri Reticulatae Pericarpium], *Pinellia* *ternata* (Thunb.) Makino [Araceae; Pinelliae Rhizoma], *Curcuma* *wenyujin* Y.H. Chen & C. Ling [Zingiberaceae; Curcumae Radix]) NR | NR | NR | Control groups (Aniracetam; bid; tablet; oral) |
| Ji 2016 | Qingnao Yizhi Granule (10g, tid; granule; oral) | *(Acorus* *tatarinowii* Schott [Acoraceae; Acori Tatarinowii Rhizoma], *Curcuma* *wenyujin* Y.H. Chen & C. Ling [Zingiberaceae; Curcumae Radix], *Rhaponticum* *uniflorum* (L.) DC. [Asteraceae; Rhapontici Radix], *Sargentodoxa* *cuneata* (Oliv.) Rehder & E.H. Wilson [Lardizabalaceae; Sargentodoxae Caulis], *Poria* *cocos* (Schw.) Wolf [Polyporaceae; Poria], *Angelica* *sinensis* (Oliv.) Diels [Apiaceae; Angelicae Sinensis Radix], *Polygonatum* *sibiricum* Red. [Asparagaceae; Polygonati Rhizoma], *Pheretima* *aspergillum* (E. Perrier) [Megascolecidae; Pheretima]) NR | NR | NR | Control groups (Nimodipine; tid; tablet; oral) |
| Jia 2019 | Gingko Biloba Leaf tablet (2tables, tid; tablet; oral) | *(Ginkgo* *biloba* L. [Ginkgoaceae; Ginkgo Folium]) NA | NR | NR | Control groups (Nimodipine; tid; tablet; oral) |
| Jiang 2021 | Wanshi Qingxin Huatan Decoction (NR, qd; granele; oral) | *Coptis* *chinensis* Franch. [Ranunculaceae; Coptidis Rhizoma] 6g, *Pinellia* *ternata* (Thunb.) Makino [Araceae; Pinelliae Rhizoma Praeparata] 10g, *Bambusa* *tuldoides* Munro [Poaceae; Bambusae Caulis in Taeniam] 10g, *Citrus* *reticulata* Blanco [Rutaceae; Citri Exocarpium Rubrum] 10g, *Citrus* *aurantium* L. [Rutaceae; Aurantii Fructus Immaturus] 10g, *Arisaema* *erubescens* (Wall.) Schott [Araceae; Arisaema cum Bile] 10g, *Prunus* *persica* (L.) Batsch [Rosaceae; Persicae Semen] 10g, *Rheum* *palmatum* L. [Polygonaceae; Rhei Radix et Rhizoma] 6g, *Trichosanthes* *kirilowii* Maxim. [Cucurbitaceae; Trichosanthis Semen] 15g, *Curcuma* *wenyujin* Y.H. Chen & C. Ling [Zingiberaceae; Curcumae Radix] 10g, *Acorus* *tatarinowii* Schott [Acoraceae; Acori Tatarinowii Rhizoma] 10g, *Poria* *cocos* (Schw.) Wolf [Polyporaceae; Poria cum Pini] 10g, *Glycyrrhiza* *uralensis* Fisch. [Fabaceae; Glycyrrhizae Radix et Rhizoma] 6g | NR | NR | Control groups (Nimodipine; tid; tablet; oral) |
| Kong 2022 | Yangxue Qingnao Granule (4g, tid; granule; oral) | *(Panax ginseng* C.A.Mey. [Araliaceae; Ginseng Radix et Rhizoma], *Whitmania pigra* Whitman [Hirudinidae; Hirudo], *Buthus martensii* Karsch [Buthidae; Scorpio], *Paeonia veitchii* Lynch. [Ranunculaceae; Paeoniae Radix Rubra], *Cryptotympana pustulata* Fabricius [Cicadidae; Cicadae Periostracum], *Eupolyphaga sinensis* Walker [Corydiidae; Eupolyphaga seu Steleophaga], *Scolopendra subspinipes* mutilans L. Koch [Scolopendridae; Scolopendra], *Santalum album* L. [Santalaceae; Santali Albi Lignum], *Dalbergia odorifera* T.Chen [Fabaceae; Dalbergiae Odoriferae Lignum], *Boswellia carterii* Birdw. [Burseraceae; Olibanum], *Ziziphus jujuba* Mill. var. spinosa (Bunge) Hu ex H.F.Chou [Rhamnaceae; Ziziphi Spinosae Semen], *Dryobalanops aromatica* C. F. Gaertn. [Dipterocarpaceae; Borneolum Syntheticum]) NA | Indication: Nourishing blood and calming the liver, promoting blood circulation and dredging collaterals. Used for headache, dizziness and blurred vision, irritability and anger, insomnia and dreaminess caused by blood deficiency and liver hyperactivity; Adverse reactions: Post-marketing monitoring data show that this product may cause the following adverse reactions: rash, itching, nausea, vomiting, abdominal distension, diarrhea, abdominal pain, gastric burning sensation, dry mouth, dizziness, headache, head distension, tinnitus, palpitations, blood pressure reduction, abnormal liver biochemical indicators, etc.; Contraindications: Pregnant women are contraindicated; patients with decompensated liver function are contraindicated; those allergic to this product and its ingredients are contraindicated; Tasly Pharmaceutical Group Co., Ltd.; Z10960082 | Grind Angelica sinensis, Ligusticum chuanxiong, Paeonia lactiflora, Rehmannia glutinosa, Uncaria rhynchophylla, Spatholobus suberectus, Prunella vulgaris, Cassia obtusifolia, and Corydalis yanhusuo into coarse powder. Decoct Concha margaritifera and Asarum heterotropoides with water twice, first with 10-fold volume for 2 hours, second with 8-fold volume for 1.5 hours. Combine the decoction, filter, and concentrate under vacuum to relative density 1.25–1.30 at 60°C. Add the above coarse powder to the concentrated extract, mix thoroughly, and granulate with an appropriate amount of dextrin and stevioside. Dry at low temperature, sift, and package to obtain the final product. | Control groups (Donepezil; qd; tablet; oral) |
| Li 2018 | Huayu Tongluo Decoction (200ml, bid; granule; oral) | *Ilex* *pubescens* Hook. & Arn. [Aquifoliaceae; Ilicis Pubescentis Radix] 15g, *Angelica* *sinensis* (Oliv.) Diels [Apiaceae; Angelicae Sinensis Radix] 12g, *Ligusticum* *chuanxiong* Hort. [Apiaceae; Chuanxiong Rhizoma] 12g, *Paeonia veitchii Lynch*. [Ranunculaceae; Paeoniae Radix Rubra] 12g, *Pheretima* *aspergillum* (E. Perrier) [Megascolecidae; Pheretima] 10g, *Bombyx* *mori* Linnaeus [Bombycidae; Bombyx Batryticatus] 10g, *Buthus* *martensii* Karsch [Buthidae; Scorpio] 5g | NR | NR | ct |
| Li M.J 2025 | Yangxue Qingnao Granule (4g, tid; granule; oral) | *(Panax ginseng* C.A.Mey. [Araliaceae; Ginseng Radix et Rhizoma], *Whitmania pigra* Whitman [Hirudinidae; Hirudo], *Buthus martensii* Karsch [Buthidae; Scorpio], *Paeonia veitchii* Lynch. [Ranunculaceae; Paeoniae Radix Rubra], *Cryptotympana pustulata* Fabricius [Cicadidae; Cicadae Periostracum], *Eupolyphaga sinensis* Walker [Corydiidae; Eupolyphaga seu Steleophaga], *Scolopendra subspinipes* mutilans L. Koch [Scolopendridae; Scolopendra], *Santalum album* L. [Santalaceae; Santali Albi Lignum], *Dalbergia odorifera* T.Chen [Fabaceae; Dalbergiae Odoriferae Lignum], *Boswellia carterii* Birdw. [Burseraceae; Olibanum], *Ziziphus jujuba* Mill. var. spinosa (Bunge) Hu ex H.F.Chou [Rhamnaceae; Ziziphi Spinosae Semen], *Dryobalanops aromatica* C. F. Gaertn. [Dipterocarpaceae; Borneolum Syntheticum]) NA | Indication: Nourishing blood and calming the liver, promoting blood circulation and dredging collaterals. Used for headache, dizziness and blurred vision, irritability and anger, insomnia and dreaminess caused by blood deficiency and liver hyperactivity; Adverse reactions: Post-marketing monitoring data show that this product may cause the following adverse reactions: rash, itching, nausea, vomiting, abdominal distension, diarrhea, abdominal pain, gastric burning sensation, dry mouth, dizziness, headache, head distension, tinnitus, palpitations, blood pressure reduction, abnormal liver biochemical indicators, etc.; Contraindications: Pregnant women are contraindicated; patients with decompensated liver function are contraindicated; those allergic to this product and its ingredients are contraindicated; Tasly Pharmaceutical Group Co., Ltd.; Z10960082 | Grind Angelica sinensis, Ligusticum chuanxiong, Paeonia lactiflora, Rehmannia glutinosa, Uncaria rhynchophylla, Spatholobus suberectus, Prunella vulgaris, Cassia obtusifolia, and Corydalis yanhusuo into coarse powder. Decoct Concha margaritifera and Asarum heterotropoides with water twice, first with 10-fold volume for 2 hours, second with 8-fold volume for 1.5 hours. Combine the decoction, filter, and concentrate under vacuum to relative density 1.25–1.30 at 60°C. Add the above coarse powder to the concentrated extract, mix thoroughly, and granulate with an appropriate amount of dextrin and stevioside. Dry at low temperature, sift, and package to obtain the final product. | Control groups (citicoline sodium; tid; tablet; oral) |
| Li L 2022 | Fufang Congrong Yizhi Capsule (0.9g, tid; capsule; oral) | *(Cistanche* *deserticola* Y.C.Ma [Orobanchaceae; Cistanches Herba], *Polygonum* *multiflorum* Thunb. [Polygonaceae; Polygoni Multiflori Radix Praeparata], *Pheretima* *aspergillum* (E. Perrier) [Megascolecidae; Pheretima], *Nelumbo* *nucifera* Gaertn. [Nelumbonaceae; Nelumbinis Folium], *Rhaponticum* *uniflorum* (L.) DC. [Asteraceae; Rhapontici Radix]) NA | Indication: Nourishing the liver and improving intelligence, promoting blood circulation and resolving turbidity, strengthening the brain and enhancing intelligence. Used for mild to moderate vascular dementia with liver and kidney deficiency combined with phlegm and blood stasis obstructing collaterals syndrome, manifested as intellectual decline, slow thinking, dull expression, forgetfulness, or mood swings, soreness and weakness of waist and knees, dizziness and tinnitus, insomnia and dreaminess; Adverse reactions: Individual cases may experience palpitations, nausea, abdominal pain, loose stools, diarrhea, epigastric fullness and distension, decreased appetite, mild skin itching, etc.; Contraindications: Pregnant women are contraindicated; those allergic to this product should use with caution; Liaoyuan Yulong Yadong Pharmaceutical Co., Ltd.; Z20080047 | Grind Polygonum multiflorum into coarse powder. Reflux-extract twice with 75% ethanol, first with 8-fold volume for 1 hour, second with 6-fold volume for 1 hour. Combine the ethanol extracts and concentrate to a clear extract of relative density 1.28–1.30 at 60°C. Boil Nelumbo nucifera leaf, Cistanche deserticola, and Rhaponticum uniflorum in water twice, first with 12-fold volume for 2 hours, second with 10-fold volume for 1 hour. Combine the decoction, filter, and concentrate to relative density 1.04–1.05 at 60°C. Cool, add ethanol to 60% alcohol content, refrigerate for 12 hours, filter, and concentrate the filtrate to a clear extract of relative density 1.28–1.30 at 60°C. Combine with the above extract and dry under vacuum below 80°C. Grind Pheretima into coarse powder, homogenize with 4-fold volume of water 5 times for 15 minutes each. Combine the homogenate, add ethanol to 10% alcohol content, centrifuge, then add ethanol to the supernatant to 51% alcohol content, centrifuge again. Wash the precipitate with acetone twice, centrifuge, and dry the precipitate under vacuum. Combine with the above dry extract, add an appropriate amount of dextrin, grind into medium powder, and encapsulate to obtain the final product. | Control groups (Nicergoline; tid; tablet; oral) |
| Liang 2020 | Yangxue Qingnao Granule (4g, tid; granule; oral) | *(Panax ginseng* C.A.Mey. [Araliaceae; Ginseng Radix et Rhizoma], *Whitmania pigra* Whitman [Hirudinidae; Hirudo], *Buthus martensii* Karsch [Buthidae; Scorpio], *Paeonia veitchii* Lynch. [Ranunculaceae; Paeoniae Radix Rubra], *Cryptotympana pustulata* Fabricius [Cicadidae; Cicadae Periostracum], *Eupolyphaga sinensis* Walker [Corydiidae; Eupolyphaga seu Steleophaga], *Scolopendra subspinipes* mutilans L. Koch [Scolopendridae; Scolopendra], *Santalum album* L. [Santalaceae; Santali Albi Lignum], *Dalbergia odorifera* T.Chen [Fabaceae; Dalbergiae Odoriferae Lignum], *Boswellia carterii* Birdw. [Burseraceae; Olibanum], *Ziziphus jujuba* Mill. var. spinosa (Bunge) Hu ex H.F.Chou [Rhamnaceae; Ziziphi Spinosae Semen], *Dryobalanops aromatica* C. F. Gaertn. [Dipterocarpaceae; Borneolum Syntheticum]) NA | Indication: Nourishing blood and calming the liver, promoting blood circulation and dredging collaterals. Used for headache, dizziness and blurred vision, irritability and anger, insomnia and dreaminess caused by blood deficiency and liver hyperactivity; Adverse reactions: Post-marketing monitoring data show that this product may cause the following adverse reactions: rash, itching, nausea, vomiting, abdominal distension, diarrhea, abdominal pain, gastric burning sensation, dry mouth, dizziness, headache, head distension, tinnitus, palpitations, blood pressure reduction, abnormal liver biochemical indicators, etc.; Contraindications: Pregnant women are contraindicated; patients with decompensated liver function are contraindicated; those allergic to this product and its ingredients are contraindicated; Tasly Pharmaceutical Group Co., Ltd.; Z10960082 | Grind Angelica sinensis, Ligusticum chuanxiong, Paeonia lactiflora, Rehmannia glutinosa, Uncaria rhynchophylla, Spatholobus suberectus, Prunella vulgaris, Cassia obtusifolia, and Corydalis yanhusuo into coarse powder. Decoct Concha margaritifera and Asarum heterotropoides with water twice, first with 10-fold volume for 2 hours, second with 8-fold volume for 1.5 hours. Combine the decoction, filter, and concentrate under vacuum to relative density 1.25–1.30 at 60°C. Add the above coarse powder to the concentrated extract, mix thoroughly, and granulate with an appropriate amount of dextrin and stevioside. Dry at low temperature, sift, and package to obtain the final product. | Control groups (Donepezil; qd; tablet; oral) |
| Liu H.S 2022 | Naoan capsule (0.8g, bid; capsule; oral) | *(Ligusticum* *chuanxiong* Hort. [Apiaceae; Chuanxiong Rhizoma], *Angelica* *sinensis* (Oliv.) Diels [Apiaceae; Angelicae Sinensis Radix], *Carthamus* *tinctorius* L. [Asteraceae; Carthami Flos], *Panax* *ginseng* C.A.Mey. [Araliaceae; Ginseng Radix et Rhizoma], *Dryobalanops* *aromatica* C. F. Gaertn. [Dipterocarpaceae; Borneolum Syntheticum]) NA | Indication: Promoting blood circulation, resolving stasis, invigorating qi and dredging collaterals. Suitable for acute and recovery phases of cerebral thrombosis with qi deficiency and blood stasis syndrome, manifested as acute hemiplegia, facial deviation, stiff tongue and slurred speech, unilateral numbness, shortness of breath and fatigue, drooling, hand and foot swelling, dark tongue or ecchymosis, etc.; Adverse reactions: Individual patients may experience head distension and dizziness in the early stage of medication, which does not require special treatment; Contraindications: Those allergic to this product are contraindicated; Anhui Yulong Yadong Pharmaceutical Co., Ltd.; Z20194060 | Grind Panax ginseng into fine powder. Reflux-extract Ligusticum chuanxiong and Angelica sinensis with 90% ethanol twice, filter, combine the filtrates, recover the ethanol, add the above Panax ginseng fine powder, mix uniformly, and dry at 70°C, then grind into fine powder. Decoct the above drug residue with water twice, filter, and combine the filtrates. Boil Carthamus tinctorius in water, then hot-extract at 70–80°C twice, filter, and combine the filtrates. Merge with the above Ligusticum chuanxiong and Angelica sinensis decoction, concentrate to an appropriate volume, cool, add ethanol to 60% alcohol content, stand overnight, filter, recover the ethanol from the filtrate, concentrate into a thick extract, dry, and grind into fine powder. Grind Borneol into fine powder, mix uniformly with the above two kinds of dry extract powder, and fill into capsules to obtain the final product. | Control groups (Nimodipine; tid; tablet; oral) |
| Liu X.F 2025 | Shenwu Yizhi Capsule (4.4g, tid; capsule; oral) | *(Panax* *ginseng* C.A.Mey. [Araliaceae; Ginseng Radix et Rhizoma], *Polygonum* *multiflorum* Thunb. [Polygonaceae; Polygoni Multiflori Radix Praeparata], *Ligusticum* *chuanxiong* Hort. [Apiaceae; Chuanxiong Rhizoma], *Curcuma* *wenyujin* Y.H. Chen & C. Ling [Zingiberaceae; Curcumae Radix], *Prunus* *persica* (L.) Batsch [Rosaceae; Persicae Semen], *Ginkgo* *biloba* L. [Ginkgoaceae; Ginkgo Folium], *Arisaema* *erubescens* (Wall.) Schott [Araceae; Arisaema cum Bile], *Polygala* *tenuifolia* Willd. [Polygalaceae; Polygalae Radix], *Acorus* *tatarinowii* Schott [Acoraceae; Acori Tatarinowii Rhizoma]) NR | NR | NR | Control groups (Donepezil; qd; tablet; oral) |
| Lu 2017 | Chaiqin Wendan Granule (NR. bid; granule, oral) | *Bupleurum* *chinense* DC. [Apiaceae; Bupleuri Radix] 20g, *Poria* *cocos* (Schw.) Wolf [Polyporaceae; Poria] 20g, *Scutellaria* *baicalensis* Georgi [Lamiaceae; Scutellariae Radix] 10g, *Citrus* *reticulata* Blanco [Rutaceae; Citri Reticulatae Pericarpium] 10g, *Bambusa* *tuldoides* Munro [Poaceae; Bambusae Caulis in Taeniam] 10g, *Pinellia* *ternata* (Thunb.) Makino [Araceae; Pinelliae Rhizoma Praeparata] 10g, *Citrus* *aurantium* L. [Rutaceae; Aurantii Fructus Immaturus] 10g, *Glycyrrhiza* *uralensis* Fisch. [Fabaceae; Glycyrrhizae Radix et Rhizoma Praeparata cum Melle] 6g | NR | NR | ct |
| Lu 2024 | Wenyang Jiangzhuo Granule (200ml. bid; granule, oral) | *Aconitum* *carmichaelii* Debeaux [Ranunculaceae; Aconiti Lateralis Radix Praeparata] 15g, *Panax* *ginseng* C.A.Mey. [Araliaceae; Ginseng Radix et Rhizoma Rubra] 8g, *Zingiber* *officinale* Roscoe [Zingiberaceae; Zingiberis Rhizoma] 10g, *Acorus* *tatarinowii* Schott [Acoraceae; Acori Tatarinowii Rhizoma] 15g, *Epimedium* *brevicornu* Maxim. [Berberidaceae; Epimedii Herba] 15g, *Rheum* *palmatum* L. [Polygonaceae; Rhei Radix et Rhizoma Praeparata cum Vino] 6g, *Panax* *notoginseng* (Burkill) F.H.Chen [Araliaceae; Notoginseng Radix et Rhizoma] 10g, *Glycyrrhiza* *uralensis* Fisch. [Fabaceae; Glycyrrhizae Radix et Rhizoma Praeparata cum Melle] 6g | NR | NR | Control groups (Donepezil; qd;tablet; oral, Hyperbaric oxygen; 5times/w; oxygen; inhalation) |
| Ma 2020 | Yangxue Qingnao Granule (4g, tid; granule; oral) | *(Panax ginseng* C.A.Mey. [Araliaceae; Ginseng Radix et Rhizoma], *Whitmania pigra* Whitman [Hirudinidae; Hirudo], *Buthus martensii* Karsch [Buthidae; Scorpio], *Paeonia veitchii* Lynch. [Ranunculaceae; Paeoniae Radix Rubra], *Cryptotympana pustulata* Fabricius [Cicadidae; Cicadae Periostracum], *Eupolyphaga sinensis* Walker [Corydiidae; Eupolyphaga seu Steleophaga], *Scolopendra subspinipes* mutilans L. Koch [Scolopendridae; Scolopendra], *Santalum album* L. [Santalaceae; Santali Albi Lignum], *Dalbergia odorifera* T.Chen [Fabaceae; Dalbergiae Odoriferae Lignum], *Boswellia carterii* Birdw. [Burseraceae; Olibanum], *Ziziphus jujuba* Mill. var. spinosa (Bunge) Hu ex H.F.Chou [Rhamnaceae; Ziziphi Spinosae Semen], *Dryobalanops aromatica* C. F. Gaertn. [Dipterocarpaceae; Borneolum Syntheticum]) NA | Indication: Nourishing blood and calming the liver, promoting blood circulation and dredging collaterals. Used for headache, dizziness and blurred vision, irritability and anger, insomnia and dreaminess caused by blood deficiency and liver hyperactivity; Adverse reactions: Post-marketing monitoring data show that this product may cause the following adverse reactions: rash, itching, nausea, vomiting, abdominal distension, diarrhea, abdominal pain, gastric burning sensation, dry mouth, dizziness, headache, head distension, tinnitus, palpitations, blood pressure reduction, abnormal liver biochemical indicators, etc.; Contraindications: Pregnant women are contraindicated; patients with decompensated liver function are contraindicated; those allergic to this product and its ingredients are contraindicated; Tasly Pharmaceutical Group Co., Ltd.; Z10960082 | Grind Angelica sinensis, Ligusticum chuanxiong, Paeonia lactiflora, Rehmannia glutinosa, Uncaria rhynchophylla, Spatholobus suberectus, Prunella vulgaris, Cassia obtusifolia, and Corydalis yanhusuo into coarse powder. Decoct Concha margaritifera and Asarum heterotropoides with water twice, first with 10-fold volume for 2 hours, second with 8-fold volume for 1.5 hours. Combine the decoction, filter, and concentrate under vacuum to relative density 1.25–1.30 at 60°C. Add the above coarse powder to the concentrated extract, mix thoroughly, and granulate with an appropriate amount of dextrin and stevioside. Dry at low temperature, sift, and package to obtain the final product. | Control groups (Donepezil; qd; tablet; oral) |
| Meng 2020 | Wenxiao Ⅲ Decoction (NR, qd; granule; oral) | *Cistanche* *deserticola* Y.C.Ma [Orobanchaceae; Cistanches Herba] 10g, *Pheretima* *aspergillum* (E. Perrier) [Megascolecidae; Pheretima] 5g, *Salvia* *miltiorrhiza* Bunge [Lamiaceae; Salviae Miltiorrhizae Radix et Rhizoma] 10g, *Curcuma* *wenyujin* Y.H. Chen & C. Ling [Zingiberaceae; Curcumae Radix] 10g, *Alpinia* *oxyphylla* Miq. [Zingiberaceae; Alpiniae Oxyphyllae Fructus] 10g | NR | NR | Control groups (Wenxiao Ⅲ placebo decoction; qd; granules; oral) |
| Qu 2019 | Gingko Biloba Leaf capsule (2caps, tid; capsule; oral) | *(Ginkgo* *biloba* L. [Ginkgoaceae; Ginkgo Folium]) NA | NR, NR, NR; Wuhu Luye Pharmaceutical Co., Ltd.; 170618 | Take standardized Ginkgo biloba extract containing 24% total flavonol glycosides and 6% terpene lactones, add an appropriate amount of excipients, mix uniformly, and fill into hard capsules, each containing 0.12g of the mixture equivalent to 80mg of Ginkgo biloba extract, to obtain the final product. | Control groups (Mecobalamin; tid; tablet; oral) |
| Qu 2020 | Gingko Biloba Leaf capsule (1caps, tid; capsule; oral) | *(Ginkgo* *biloba* L. [Ginkgoaceae; Ginkgo Folium]) NA | Indication: Promoting blood circulation, resolving stasis and dredging collaterals. Used for chest bi and heart pain, stroke, hemiplegia, stiff tongue and slurred speech caused by blood stasis obstructing collaterals; coronary heart disease with stable angina pectoris and cerebral infarction with the above symptoms; Adverse reactions: Post-marketing adverse reaction monitoring data show that ginkgo leaf oral preparations may cause the following adverse reactions: gastrointestinal system (nausea, vomiting, dry mouth, abdominal distension, abdominal pain, abdominal discomfort, excessive gastric acid, etc., with case reports of gastrointestinal bleeding); nervous system (dizziness, headache, etc., with case reports of local numbness); skin and appendages (rash, itching, etc.); cardiovascular system (chest tightness, palpitations, etc., with case reports of blood pressure elevation or reduction); others (fatigue, allergic or allergic-like reactions, etc., with case reports of gum bleeding and nosebleed); Contraindications: Those allergic to this product and its ingredients are contraindicated; Shanghai Sine Bilot Pharmaceutical Co., Ltd.; Z20010169 | Take standardized Ginkgo biloba extract, add an appropriate amount of excipients, mix uniformly, and fill into hard capsules to obtain the final product. | Control groups (Mecobalamin; tid; tablet; oral) |
| Ren 2022 | Peiyuan Kaizhi Decoction (300ml, bid; granule; oral) | *Whitmania pigra* Whitman [Hirudinidae; Hirudo] 4g, *Glycyrrhiza* *uralensis* Fisch. [Fabaceae; Glycyrrhizae Radix et Rhizoma] 6g, *Carthamus* *tinctorius* L. [Asteraceae; Carthami Flos] 8g, *Cyperus* *rotundus* L. [Cyperaceae; Cyperi Rhizoma] 10g, *Codonopsis* *pilosula* (Franch.) Nannf. [Campanulaceae; Codonopsis Radix] 10g, *Pheretima* *aspergillum* (E. Perrier) [Megascolecidae; Pheretima] 12g, *Alpinia* *oxyphylla* Miq. [Zingiberaceae; Alpiniae Oxyphyllae Fructus] 12g, *Rehmannia* *glutinosa* (Gaertn.) Libosch. exFisch. et Mey. [Orobanchaceae; Rehmanniae Radix Praeparata] 12g, *Atractylodes* *macrocephala* Koidz. [Asteraceae; Atractylodis Macrocephalae Rhizoma] 12g, *Gallus* *gallus domesticus* Brisson [Phasianidae; Endothelium Corneum Gigeriae Galli] 15g, *Gastrodia* *elata* Bl. [Orchidaceae; Gastrodiae Rhizoma] 15g, *Acorus* *tatarinowii* Schott [Acoraceae; Acori Tatarinowii Rhizoma] 15g, *Poria* *cocos* (Schw.) Wolf [Polyporaceae; Poria] 15g, *Cistanche* *deserticola* Y.C.Ma [Orobanchaceae; Cistanches Herba] 15g, *Cervus* *elaphus / Cervus nippon* Linnaeus / Temminck [Cervidae; Colla Corni Cervi] 15g, *Chinemys* *reevesii* (Gray) [Geoemydidae; Testudinis Carapacis et Plastri Colla] 15g, *Polygala* *tenuifolia* Willd. [Polygalaceae; Polygalae Radix] 20g, *Salvia* *miltiorrhiza* Bunge [Lamiaceae; Salviae Miltiorrhizae Radix et Rhizoma] 30g | NR | NR | ct |
| Ruan 2023 | Yangxue Qingnao Granule (4g, tid; granule; oral) | *(Panax ginseng* C.A.Mey. [Araliaceae; Ginseng Radix et Rhizoma], *Whitmania pigra* Whitman [Hirudinidae; Hirudo], *Buthus martensii* Karsch [Buthidae; Scorpio], *Paeonia veitchii* Lynch. [Ranunculaceae; Paeoniae Radix Rubra], *Cryptotympana pustulata* Fabricius [Cicadidae; Cicadae Periostracum], *Eupolyphaga sinensis* Walker [Corydiidae; Eupolyphaga seu Steleophaga], *Scolopendra subspinipes* mutilans L. Koch [Scolopendridae; Scolopendra], *Santalum album* L. [Santalaceae; Santali Albi Lignum], *Dalbergia odorifera* T.Chen [Fabaceae; Dalbergiae Odoriferae Lignum], *Boswellia carterii* Birdw. [Burseraceae; Olibanum], *Ziziphus jujuba* Mill. var. spinosa (Bunge) Hu ex H.F.Chou [Rhamnaceae; Ziziphi Spinosae Semen], *Dryobalanops aromatica* C. F. Gaertn. [Dipterocarpaceae; Borneolum Syntheticum]) NA | Indication: Nourishing blood and calming the liver, promoting blood circulation and dredging collaterals. Used for headache, dizziness and blurred vision, irritability and anger, insomnia and dreaminess caused by blood deficiency and liver hyperactivity; Adverse reactions: Post-marketing monitoring data show that this product may cause the following adverse reactions: rash, itching, nausea, vomiting, abdominal distension, diarrhea, abdominal pain, gastric burning sensation, dry mouth, dizziness, headache, head distension, tinnitus, palpitations, blood pressure reduction, abnormal liver biochemical indicators, etc.; Contraindications: Pregnant women are contraindicated; patients with decompensated liver function are contraindicated; those allergic to this product and its ingredients are contraindicated; Tasly Pharmaceutical Group Co., Ltd.; Z10960082 | Grind Angelica sinensis, Ligusticum chuanxiong, Paeonia lactiflora, Rehmannia glutinosa, Uncaria rhynchophylla, Spatholobus suberectus, Prunella vulgaris, Cassia obtusifolia, and Corydalis yanhusuo into coarse powder. Decoct Concha margaritifera and Asarum heterotropoides with water twice, first with 10-fold volume for 2 hours, second with 8-fold volume for 1.5 hours. Combine the decoction, filter, and concentrate under vacuum to relative density 1.25–1.30 at 60°C. Add the above coarse powder to the concentrated extract, mix thoroughly, and granulate with an appropriate amount of dextrin and stevioside. Dry at low temperature, sift, and package to obtain the final product. | Control groups (Donepezil; qd; tablet; oral) |
| Shi 2017 | Gingko Biloba Leaf tablet (19.2g, tid; tablet; oral) | *(Ginkgo* *biloba* L. [Ginkgoaceae; Ginkgo Folium]) NA | Indication: Promoting blood circulation, resolving stasis and dredging collaterals. Used for chest bi and heart pain, stroke, hemiplegia, stiff tongue and slurred speech caused by blood stasis obstructing collaterals; coronary heart disease with stable angina pectoris and cerebral infarction with the above symptoms; Adverse reactions: Post-marketing adverse reaction monitoring data show that ginkgo leaf oral preparations may cause the following adverse reactions: gastrointestinal system (nausea, vomiting, dry mouth, abdominal distension, abdominal pain, abdominal discomfort, excessive gastric acid, etc., with case reports of gastrointestinal bleeding); nervous system (dizziness, headache, etc., with case reports of local numbness); skin and appendages (rash, itching, etc.); cardiovascular system (chest tightness, palpitations, etc., with case reports of blood pressure elevation or reduction); others (fatigue, allergic or allergic-like reactions, etc., with case reports of gum bleeding and nosebleed); Contraindications: Those allergic to this product and its ingredients are contraindicated; Hangzhou Conba Pharmaceutical Co., Ltd.; Z20063069 | Extract Ginkgo biloba leaves with 60–70% ethanol by percolation or reflux, concentrate the ethanol extract under vacuum, and suspend in water. Remove impurities by petroleum ether extraction, then extract with ethyl acetate. Concentrate the ethyl acetate extract and dissolve in acetone for crystallization. Dissolve the purified Ginkgo leaf extract in an appropriate amount of ethanol, mix uniformly with medicinal starch as filler, granulate with 95% ethanol as wetting agent, dry, and sift. Fill the granules into hard capsules, with each capsule containing 9.6 mg of total flavonol glycosides and 2.4 mg of terpene lactones, to obtain the final product. | Control groups (Nimodipine; tid; tablet; oral) |
| Sun X.J 2023 | Fufang Congrong Yizhi Capsule (0.9g, tid; capsule; oral) | *(Cistanche* *deserticola* Y.C.Ma [Orobanchaceae; Cistanches Herba], *Polygonum* *multiflorum* Thunb. [Polygonaceae; Polygoni Multiflori Radix Praeparata], *Pheretima* *aspergillum* (E. Perrier) [Megascolecidae; Pheretima], *Nelumbo* *nucifera* Gaertn. [Nelumbonaceae; Nelumbinis Folium], *Rhaponticum* *uniflorum* (L.) DC. [Asteraceae; Rhapontici Radix]) NA | Indication: Nourishing the liver and improving intelligence, promoting blood circulation and resolving turbidity, strengthening the brain and enhancing intelligence. Used for mild to moderate vascular dementia with liver and kidney deficiency combined with phlegm and blood stasis obstructing collaterals syndrome, manifested as intellectual decline, slow thinking, dull expression, forgetfulness, or mood swings, soreness and weakness of waist and knees, dizziness and tinnitus, insomnia and dreaminess; Adverse reactions: Individual cases may experience palpitations, nausea, abdominal pain, loose stools, diarrhea, epigastric fullness and distension, decreased appetite, mild skin itching, etc.; Contraindications: Pregnant women are contraindicated; those allergic to this product should use with caution; Liaoyuan Yulong Yadong Pharmaceutical Co., Ltd.; Z20080047 | Grind Polygonum multiflorum into coarse powder. Reflux-extract twice with 75% ethanol, first with 8-fold volume for 1 hour, second with 6-fold volume for 1 hour. Combine the ethanol extracts and concentrate to a clear extract of relative density 1.28–1.30 at 60°C. Boil Nelumbo nucifera leaf, Cistanche deserticola, and Rhaponticum uniflorum in water twice, first with 12-fold volume for 2 hours, second with 10-fold volume for 1 hour. Combine the decoction, filter, and concentrate to relative density 1.04–1.05 at 60°C. Cool, add ethanol to 60% alcohol content, refrigerate for 12 hours, filter, and concentrate the filtrate to a clear extract of relative density 1.28–1.30 at 60°C. Combine with the above extract and dry under vacuum below 80°C. Grind Pheretima into coarse powder, homogenize with 4-fold volume of water 5 times for 15 minutes each. Combine the homogenate, add ethanol to 10% alcohol content, centrifuge, then add ethanol to the supernatant to 51% alcohol content, centrifuge again. Wash the precipitate with acetone twice, centrifuge, and dry the precipitate under vacuum. Combine with the above dry extract, add an appropriate amount of dextrin, grind into medium powder, and encapsulate to obtain the final product. | Control groups (Donepezil; qd; tablet; oral) |
| Wang J 2023 | Tongqiao Huoxue Decoction (50ml, bid; granule; oral) | *Prunus* *persica* (L.) Batsch [Rosaceae; Persicae Semen] 15g, *Paeonia veitchii Lynch*. [Ranunculaceae; Paeoniae Radix Rubra] 15g, *Ligusticum* *chuanxiong* Hort. [Apiaceae; Chuanxiong Rhizoma] 15g, *Carthamus* *tinctorius* L. [Asteraceae; Carthami Flos] 10g, *Zingiber* *officinale* Roscoe [Zingiberaceae; Zingiberis Rhizoma Recens] 10g, *Allium* *fistulosum* L. [Amaryllidaceae; Allii Fistulosi Caulis] 8g, *Artificial Musk* [—; Moschus Artifactus] 0.15g, *Yellow Rice Wine* [—; Vinum Aureum] 250ml | NR | NR | ct |
| Wang 2024 | Gingko Biloba Leaf capsule (1caps, tid; capsule; oral) | *(Ginkgo* *biloba* L. [Ginkgoaceae; Ginkgo Folium]) NA | Indication: Promoting blood circulation, resolving stasis and dredging collaterals. Used for chest bi and heart pain, stroke, hemiplegia, stiff tongue and slurred speech caused by blood stasis obstructing collaterals; coronary heart disease with stable angina pectoris and cerebral infarction with the above symptoms; Adverse reactions: Some patients may experience stomach discomfort after taking the medicine; Contraindications: Those allergic to this product are contraindicated; Guangxi Shengte Pharmaceutical Co., Ltd.; Z20040102 | Dissolve β-cyclodextrin in 25% ethanol solution at a ratio of 1–3:10–20 to prepare the spraying liquid. Place Ginkgo biloba extract and talc powder into a fluid-bed granulator, spray the β-cyclodextrin solution onto the fluidized powder surface to accomplish wetting, drying, particle coating, granulation, and separation in one step. Add an appropriate amount of magnesium stearate, mix uniformly, and fill the resulting granules into hard capsules. The product contains 30–50 parts of Ginkgo leaf extract, 120–180 parts of β-cyclodextrin, 7–12 parts of talc, and 0.5–1.5 parts of magnesium stearate, with improved bioavailability, solubility, and stability. | Control groups (Mecobalamin; tid; tablet; oral) |
| Wu 2024 (Wu et al., 2024) | Peiyuan Tongnao Capsule (1.8g, tid; capsules; oral) | *(Polygonum* *multiflorum* Thunb. [Polygonaceae; Polygoni Multiflori Radix], *Rehmannia* *glutinosa* (Gaertn.) Libosch. exFisch. et Mey. [Orobanchaceae; Rehmanniae Radix Praeparata], *Asparagus* *cochinchinensis* (Lour.) Merr. [Asparagaceae; Asparagi Radix], *Chinemys* *reevesii* (Gray) [Geoemydidae; Testudinis Plastrum], *Cervus* *elaphus / Cervus nippon* Linnaeus / Temminck [Cervidae; Cervi Cornu], *Cistanche* *deserticola* Y.C.Ma [Orobanchaceae; Cistanches Herba], *Cinnamomum* *cassia* Presl [Lauraceae; Cinnamomi Cortex], *Paeonia veitchii Lynch*. [Ranunculaceae; Paeoniae Radix Rubra], *Buthus* *martensii* Karsch [Buthidae; Scorpio], *Whitmania pigra* Whitman [Hirudinidae; Hirudo], *Pheretima* *aspergillum* (E. Perrier) [Megascolecidae; Pheretima], *Crataegus* *pinnatifida* Bunge [Rosaceae; Crataegi Fructus], *Poria* *cocos* (Schw.) Wolf [Polyporaceae; Poria], *Glycyrrhiza* *uralensis* Fisch. [Fabaceae; Glycyrrhizae Radix et Rhizoma Praeparata cum Melle]) NA | Indication: Tonifying the kidney and filling essence, extinguishing wind and dredging collaterals. Used for ischemic stroke in the meridian recovery period with kidney essence deficiency and blood stasis obstructing collaterals syndrome, manifested as hemiplegia, facial deviation, unclear speech, unilateral numbness, dizziness and tinnitus, soreness and weakness of waist and knees, deep and fine pulse; Adverse reactions: Individual patients may experience nausea after taking the medicine, generally does not affect continued medication; one patient experienced somnolence and fatigue during clinical trials, symptoms resolved spontaneously without discontinuing medication, but causality could not be determined; Contraindications: Pregnant women are contraindicated; postpartum women should use with caution; Henan Lingrui Pharmaceutical Co., Ltd.; Z20000022 | Grind Cervus nippon, Scorpion, Hirudo, Pheretima, and Cinnamomum cassia into fine powder separately. Decoct Chinemys reevesii with water three times, 6 hours each, filter, and combine the decoction. Soak Polygonum multiflorum, Rehmannia glutinosa, Asparagus cochinchinensis, Cistanche deserticola, Paeonia lactiflora, Crataegus pinnatifida, Poria cocos, and Glycyrrhiza uralensis in water for 1 hour, then decoct three times, 1.5 hours each, filter, and combine the filtrate. Add the above Chinemys reevesii decoction, concentrate under vacuum to relative density 1.28–1.33 at 60–70°C to obtain a thick paste. Add Scorpion and Pheretima powder, mix uniformly, dry, and grind into fine powder. Add Cervus nippon, Hirudo, and Cinnamomum cassia fine powder, mix thoroughly, add an appropriate amount of excipient, and fill into capsules to obtain the final product. | Control groups (Atorvastatin Calcium; qd; tablet; oral) |
| Xiao 2021 | Ginkgo Biloba Extract (40mg; tid; capsule; oral) | *(Ginkgo* *biloba* L. [Ginkgoaceae; Ginkgo Folium]) NA | Indication: Mainly used for cerebral and peripheral blood circulation disorders. 1. Acute and chronic cerebral insufficiency and its sequelae: stroke, lack of concentration, memory decline, dementia. 2. Ear blood flow and nerve disorders: tinnitus, vertigo, hearing loss, labyrinthine syndrome. 3. Eye blood flow and nerve disorders: diabetic retinopathy and nerve disorders, age-related macular degeneration, blurred vision, chronic glaucoma. 4. Peripheral circulation disorders: various arterial occlusive diseases, intermittent claudication, numbness and coldness of hands and feet, limb pain; Adverse reactions: Post-marketing monitoring data suggest that adverse reactions of this product include: gastrointestinal system (mild gastrointestinal discomfort); skin and subcutaneous tissue/immune system (hypersensitivity reactions); nervous system (headache); vascular system (case reports of single organ bleeding); Contraindications: Those allergic to any ingredient in this product are contraindicated; Dr. Willmar Schwabe GmbH & Co. KG, Germany; H20140768 | Extract dried and chopped Ginkgo biloba leaves with aqueous acetone solution (approximately 60% by weight acetone) at 40 to 60°C. Remove the acetone until a maximum content of 5% by weight is reached. Dilute the remaining concentrated aqueous solution with water to a solid content of at most 10% by weight, cool this solution to 12°C, and remove the resulting precipitate. Add ammonium sulfate (approximately 30% by weight) to the remaining aqueous solution, and extract the resulting solution with methyl ethyl ketone or a mixture of methyl ethyl ketone and acetone (6/4). Concentrate the resulting organic phase, and dilute the resulting concentrate with water and ethanol to obtain a solution containing approximately 50% by weight water, approximately 50% by weight ethanol, and approximately 10% by weight solids. Add aqueous lead glycolate solution to the solution thus obtained, and remove the resulting precipitate. Extract the remaining aqueous ethanolic solution with heptane to further remove alkylphenol metabolites. Concentrate the remaining aqueous ethanolic solution under reduced pressure, and add approximately 20% by weight ammonium sulfate. Extract the resulting solution with a mixture of methyl ethyl ketone and ethanol (6/4). Dry the resulting organic phase with at most 20% by weight ammonium sulfate and concentrate, and add ethanol to achieve an ethanol content of at least 80% by weight. Maintain the solution temperature below 12°C for 2 to 12 hours and filter. Concentrate the resulting filtrate under reduced pressure and dry to obtain a dry extract with a water content of less than 5%. | Control groups (Donepezil; qd; tablet; oral) |
| Xie 2020 | Yangxue QingnaoTablet (2.5g, tid; tablet; oral) | *(Panax ginseng* C.A.Mey. [Araliaceae; Ginseng Radix et Rhizoma], *Whitmania pigra* Whitman [Hirudinidae; Hirudo], *Buthus martensii* Karsch [Buthidae; Scorpio], *Paeonia veitchii* Lynch. [Ranunculaceae; Paeoniae Radix Rubra], *Cryptotympana pustulata* Fabricius [Cicadidae; Cicadae Periostracum], *Eupolyphaga sinensis* Walker [Corydiidae; Eupolyphaga seu Steleophaga], *Scolopendra subspinipes* mutilans L. Koch [Scolopendridae; Scolopendra], *Santalum album* L. [Santalaceae; Santali Albi Lignum], *Dalbergia odorifera* T.Chen [Fabaceae; Dalbergiae Odoriferae Lignum], *Boswellia carterii* Birdw. [Burseraceae; Olibanum], *Ziziphus jujuba* Mill. var. spinosa (Bunge) Hu ex H.F.Chou [Rhamnaceae; Ziziphi Spinosae Semen], *Dryobalanops aromatica* C. F. Gaertn. [Dipterocarpaceae; Borneolum Syntheticum]) NA | Indication: Nourishing blood and calming the liver, promoting blood circulation and dredging collaterals. Used for headache, dizziness and blurred vision, irritability and anger, insomnia and dreaminess caused by blood deficiency and liver hyperactivity; Adverse reactions: Post-marketing monitoring data show that this product may cause the following adverse reactions: rash, itching, nausea, vomiting, abdominal distension, diarrhea, abdominal pain, gastric burning sensation, dry mouth, dizziness, headache, head distension, tinnitus, palpitations, blood pressure reduction, abnormal liver biochemical indicators, etc.; Contraindications: Pregnant women are contraindicated; patients with decompensated liver function are contraindicated; those allergic to this product and its ingredients are contraindicated; Tasly Pharmaceutical Group Co., Ltd.; Z10960082 | Grind Angelica sinensis, Ligusticum chuanxiong, Paeonia lactiflora, Rehmannia glutinosa, Uncaria rhynchophylla, Spatholobus suberectus, Prunella vulgaris, Cassia obtusifolia, and Corydalis yanhusuo into coarse powder. Decoct Concha margaritifera and Asarum heterotropoides with water twice, first with 10-fold volume for 2 hours, second with 8-fold volume for 1.5 hours. Combine the decoction, filter, and concentrate under vacuum to relative density 1.25–1.30 at 60°C. Add the above coarse powder to the concentrated extract, mix thoroughly, and granulate with an appropriate amount of dextrin and stevioside. Dry at low temperature, sift, and package to obtain the final product. | Control groups (Donepezil; qd; tablet; oral) |
| Xu 2025 | Zhishe Tongluo capsule (4caps, tid; capsule; oral) | *(Astragalus* *membranaceus* (Fisch.) Bunge [Fabaceae; Astragali Radix], *Panax* *ginseng* C.A.Mey. [Araliaceae; Ginseng Radix et Rhizoma], *Gastrodia* *elata* Bl. [Orchidaceae; Gastrodiae Rhizoma], *Salvia* *miltiorrhiza* Bunge [Lamiaceae; Salviae Miltiorrhizae Radix et Rhizoma], *Carthamus* *tinctorius* L. [Asteraceae; Carthami Flos], *Pueraria* *lobata* (Willd.) Ohwi [Fabaceae; Puerariae Lobatae Radix], *Ligusticum* *chuanxiong* Hort. [Apiaceae; Chuanxiong Rhizoma], *Acorus* *tatarinowii* Schott [Acoraceae; Acori Tatarinowii Rhizoma], *Curcuma* *wenyujin* Y.H. Chen & C. Ling [Zingiberaceae; Curcumae Radix], *Whitmania pigra* Whitman [Hirudinidae; Hirudo], *Dryobalanops* *aromatica* C. F. Gaertn. [Dipterocarpaceae; Borneolum Syntheticum], *Zaocys* *dhumnades* (Cantor) [Colubridae; Zaocys]) NA | Indication: Invigorating qi and promoting blood circulation, extinguishing wind and dredging collaterals. Used for stroke in the meridian (mild to moderate cerebral infarction) recovery period with qi deficiency and blood stasis syndrome, manifested as hemiplegia, unilateral numbness, deviated mouth and tongue, stiff tongue and slurred speech, spontaneous sweating, shortness of breath and fatigue, deep and fine or stringy pulse; Adverse reactions: NR; Contraindications: Pregnant women are contraindicated; Shaanxi Jianmin Pharmaceutical Co., Ltd.; Z20090658 | Take Astragalus membranaceus, Panax ginseng, Gastrodia elata, Salvia miltiorrhiza, Carthamus tinctorius, Pueraria lobata, Ligusticum chuanxiong, Acorus tatarinowii, Curcuma aromatica, Hirudo, Borneolum syntheticum, and Zaocys dhumnades, process into fine powder, add an appropriate amount of excipients, mix uniformly, and fill into hard capsules, each containing 0.5g, to obtain the final product. | Control groups (Hyperbaric oxygen; qd; oxygen; inhalation) |
| Yang P 2016 | Tongxinluo Capsule (0.78g, tid; capsule; oral) | *(Panax ginseng* C.A.Mey. [Araliaceae; Ginseng Radix et Rhizoma], *Whitmania pigra* Whitman [Hirudinidae; Hirudo], *Buthus martensii* Karsch [Buthidae; Scorpio], *Paeonia veitchii Lynch*. [Ranunculaceae; Paeoniae Radix Rubra], *Cryptotympana pustulata* Fabricius [Cicadidae; Cicadae Periostracum], *Eupolyphaga sinensis* Walker [Corydiidae; Eupolyphaga seu Steleophaga], *Scolopendra subspinipes* mutilans L. Koch [Scolopendridae; Scolopendra], *Santalum album* L. [Santalaceae; Santali Albi Lignum], *Dalbergia odorifera* T.Chen [Fabaceae; Dalbergiae Odoriferae Lignum], *Boswellia carterii* Birdw. [Burseraceae; Olibanum], *Ziziphus jujuba* Mill. var. spinosa (Bunge) Hu ex H.F.Chou [Rhamnaceae; Ziziphi Spinosae Semen], *Dryobalanops aromatica* C. F. Gaertn. [Dipterocarpaceae; Borneolum Syntheticum]) NA | Indication: Invigorating qi and promoting blood circulation, dredging collaterals and relieving pain. Used for coronary heart disease and angina pectoris with heart qi deficiency and blood stasis obstructing collaterals, manifested as chest oppression, stabbing pain, colicky pain, fixed pain, palpitations, spontaneous sweating, shortness of breath and fatigue, dark purple tongue or ecchymosis, fine and rough or intermittent pulse. Also used for stroke of qi deficiency and blood stasis obstructing collaterals type, manifested as hemiplegia or unilateral numbness, deviated mouth and tongue, slurred speech; Adverse reactions: Occasional gastrointestinal discomfort such as nausea, vomiting, abdominal pain, abdominal distension, diarrhea, and stomach discomfort, as well as rash, itching, dizziness, etc.; Contraindications: Hemorrhagic diseases, pregnant women, women during menstruation, and yin deficiency with excessive fire type stroke are contraindicated; Shijiazhuang Yiling Pharmaceutical Co., Ltd.; Z19980015 | Grind Scorpion, Leech, Centipede, Ground Beetle, and Cicada Slough into ultrafine powder below 10 μm. Distill Sandalwood and Dalbergia odorifera with water vapor to collect volatile oil; the residue is then boiled in water, filtered, and concentrated under vacuum. Soak Ginseng in 70% ethanol, reflux-extract, recover the ethanol, and concentrate under vacuum; the residue is then boiled in water, filtered, and concentrated under vacuum. Boil Sour Jujube Seed and Red Peony Root in water, filter, and concentrate under vacuum. Combine all concentrated extracts, mix thoroughly with the ultrafine powder, spray-dry, and grind. Add an appropriate amount of excipient to form granules. Incorporate the volatile oil from Sandalwood and Dalbergia odorifera, mix well with Borneol, and encapsulate to obtain the final product. | Control groups (Aspirin enteric-coated tablets; tid; tablet; oral + Atorvastatin; qd; tablet; oral + Nimodipine; tid; tablet; oral) |
| Yang 2016 | Yangxue Qingnao Granule (4g, tid; granule; oral) | *(Panax ginseng* C.A.Mey. [Araliaceae; Ginseng Radix et Rhizoma], *Whitmania pigra* Whitman [Hirudinidae; Hirudo], *Buthus martensii* Karsch [Buthidae; Scorpio], *Paeonia veitchii* Lynch. [Ranunculaceae; Paeoniae Radix Rubra], *Cryptotympana pustulata* Fabricius [Cicadidae; Cicadae Periostracum], *Eupolyphaga sinensis* Walker [Corydiidae; Eupolyphaga seu Steleophaga], *Scolopendra subspinipes* mutilans L. Koch [Scolopendridae; Scolopendra], *Santalum album* L. [Santalaceae; Santali Albi Lignum], *Dalbergia odorifera* T.Chen [Fabaceae; Dalbergiae Odoriferae Lignum], *Boswellia carterii* Birdw. [Burseraceae; Olibanum], *Ziziphus jujuba* Mill. var. spinosa (Bunge) Hu ex H.F.Chou [Rhamnaceae; Ziziphi Spinosae Semen], *Dryobalanops aromatica* C. F. Gaertn. [Dipterocarpaceae; Borneolum Syntheticum]) NA | Indication: Nourishing blood and calming the liver, promoting blood circulation and dredging collaterals. Used for headache, dizziness and blurred vision, irritability and anger, insomnia and dreaminess caused by blood deficiency and liver hyperactivity; Adverse reactions: Post-marketing monitoring data show that this product may cause the following adverse reactions: rash, itching, nausea, vomiting, abdominal distension, diarrhea, abdominal pain, gastric burning sensation, dry mouth, dizziness, headache, head distension, tinnitus, palpitations, blood pressure reduction, abnormal liver biochemical indicators, etc.; Contraindications: Pregnant women are contraindicated; patients with decompensated liver function are contraindicated; those allergic to this product and its ingredients are contraindicated; Tasly Pharmaceutical Group Co., Ltd.; Z10960082 | Grind Angelica sinensis, Ligusticum chuanxiong, Paeonia lactiflora, Rehmannia glutinosa, Uncaria rhynchophylla, Spatholobus suberectus, Prunella vulgaris, Cassia obtusifolia, and Corydalis yanhusuo into coarse powder. Decoct Concha margaritifera and Asarum heterotropoides with water twice, first with 10-fold volume for 2 hours, second with 8-fold volume for 1.5 hours. Combine the decoction, filter, and concentrate under vacuum to relative density 1.25–1.30 at 60°C. Add the above coarse powder to the concentrated extract, mix thoroughly, and granulate with an appropriate amount of dextrin and stevioside. Dry at low temperature, sift, and package to obtain the final product. | ct |
| Zhang SX 2019 | Jiawei Dihuang Yin Decoction (NR, bid; granule; oral) | *Rehmannia* *glutinosa* (Gaertn.) Libosch. ex Fisch. et Mey. [Orobanchaceae; Rehmanniae Radix Praeparata] 30g, *Cornus* *officinalis* Siebold & Zucc. [Cornaceae; Corni Fructus] 15g, *Ophiopogon* *japonicus* (L. f.) Ker-Gawl. [Asparagaceae; Ophiopogonis Radix] 15g, *Dendrobium* *nobile* Lindl. [Orchidaceae; Dendrobii Caulis] 15g, *Cistanche* *deserticola* Y.C.Ma [Orobanchaceae; Cistanches Herba] 15g, *Morinda* *officinalis* How [Rubiaceae; Morindae Officinalis Radix] 15g, *Angelica* *sinensis* (Oliv.) Diels [Apiaceae; Angelicae Sinensis Radix] 10g | NR | NR | Control groups (Oxiracetam; bid; capsule; oral) |
| Zhang 2021 | Tongqiao Huoxue Decoction (100ml, bid; decoction; oral) | *(Polygonum* *multiflorum* Thunb. [Polygonaceae; Polygoni Multiflori Radix], *Rehmannia* *glutinosa* (Gaertn.) Libosch. ex Fisch. et Mey. [Orobanchaceae; Rehmanniae Radix Praeparata], *Asparagus* *cochinchinensis* (Lour.) Merr. [Asparagaceae; Asparagi Radix], *Chinemys* *reevesii* (Gray) [Geoemydidae; Testudinis Plastrum], *Cervus* *elaphus / Cervus nippon* Linnaeus / Temminck [Cervidae; Cervi Cornu], *Cistanche* *deserticola* Y.C.Ma [Orobanchaceae; Cistanches Herba], *Cinnamomum* *cassia* Presl [Lauraceae; Cinnamomi Cortex], *Paeonia veitchii Lynch*. [Ranunculaceae; Paeoniae Radix Rubra], *Buthus* *martensii* Karsch [Buthidae; Scorpio], *Whitmania pigra* Whitman [Hirudinidae; Hirudo], *Pheretima* *aspergillum* (E. Perrier) [Megascolecidae; Pheretima], *Crataegus* *pinnatifida* Bunge [Rosaceae; Crataegi Fructus], *Poria* *cocos* (Schw.) Wolf [Polyporaceae; Poria], *Glycyrrhiza* *uralensis* Fisch. [Fabaceae; Glycyrrhizae Radix et Rhizoma Praeparata cum Melle]) NR | NR | NR | Control groups (Donepezil; qd; tablet; oral + Nimodipine; tid; tablet; oral) |
| Zhang F.P 2025 | Fufang Huonaoshu Capsule (3caps, bid; capsules; oral) | *(Sus* *scrofa domesticus* Brisson [Suidae; Porcus Cerebrum], *Schisandra* *chinensis* (Turcz.) Baill. [Schisandraceae; Schisandrae Chinensis Fructus], *Ophiopogon* *japonicus* (L. f.) Ker-Gawl. [Asparagaceae; Ophiopogonis Radix], *Panax* *ginseng* C.A.Mey. [Araliaceae; Ginseng Radix et Rhizoma], *Lycium* *barbarum* L. [Solanaceae; Lycii Fructus], *Rehmannia* *glutinosa* (Gaertn.) Libosch. exFisch. et Mey. [Orobanchaceae; Rehmanniae Radix], *Salvia* *miltiorrhiza* Bunge [Lamiaceae; Salviae Miltiorrhizae Radix et Rhizoma]) NA | Indication: Invigorating qi and nourishing blood, strengthening the brain and enhancing intelligence. Used for forgetfulness with qi and blood deficiency syndrome, memory decline, fatigue and weakness, dizziness and palpitations, and improvement of the above symptoms in senile dementia; Adverse reactions: NR; Contraindications: NR; Jilin Huinan Changlong Biochemical Pharmaceutical Co., Ltd.; Z20026288 | Take Sus scrofa brain, Schisandra chinensis, Ophiopogon japonicus, Panax ginseng, Lycium barbarum, Rehmannia glutinosa, and Salvia miltiorrhiza, process into fine powder, add an appropriate amount of excipients, mix uniformly, and fill into hard capsules, each containing 0.25g, to obtain the final product. | Control groups (Citicoline Sodium; tid; tablet; oral) |
| Zhang DC 2019 | Zhilong Huoxue Tongyu capsule (0.4g, tid; capsule; oral) | *(Astragalus* *membranaceus* (Fisch.) Bunge [Fabaceae; Astragali Radix], *Whitmania pigra* Whitman [Hirudinidae; Hirudo], *Pheretima* *aspergillum* (E. Perrier) [Megascolecidae; Pheretima], *Sargentodoxa* *cuneata* (Oliv.) Rehder & E.H. Wilson [Lardizabalaceae; Sargentodoxae Caulis], *Cinnamomum* *cassia* Presl [Lauraceae; Cinnamomi Ramulus]) NA | Indication: Invigorating qi and promoting blood circulation, resolving stasis and dredging collaterals. Used for ischemic cerebrovascular diseases, coronary heart disease, venous thrombotic diseases, etc.; Adverse reactions: NR; Contraindications: NR; Southwest Medical University Affiliated Hospital of Traditional Chinese Medicine; Chuan Pharmaceutical Preparation Standard: Z20070528 | Soak dried Hirudo nipponica Whitman (0.32g) and Pheretima aspergillum (1.7g) in 2.85 mL of 60% ethanol for 7 days, followed by filtration and collection of filtrate A. Decoct Astragalus membranaceus (2.3g), Cinnamomum cassia twigs (0.86g), and Sargentodoxa cuneata (1.7g) twice with 13.51 mL of water for 1 hour each time, and collect the volatile fraction. Combine the two decoctions, filter, and concentrate at 80°C to relative density 1.05–1.10. Add ethanol to the concentrate to achieve 50% alcohol content, stand for 12 hours, and filter. Combine the resulting filtrate B with ethanolic infusion A. Recover the alcohol, boil and concentrate the mixture at 80°C to relative density 1.20. Add 0.2g of dextrin as excipient, boil, granulate, and crush into fine powder. Spray and mix with the volatile fraction collected previously. Finally, fill 0.4g of the powdered mixture into a capsule shell to make one capsule dosage form. The whole drug extract ratio (DER) is 12.9%. | ct |
| Zhang XH 2021 | Kaiqiao Tongluo Decoction (200ml, bid; decoction; oral) | *Moschus* [Cervidae; Moschus] 0.15g, *Pinellia* *ternata* (Thunb.) Makino [Araceae; Pinelliae Rhizoma Praeparata] 15g, *Arisaema* *erubescens* (Wall.) Schott [Araceae; Arisaema cum Bile] 15g, *Curcuma* *wenyujin* Y.H. Chen & C. Ling [Zingiberaceae; Curcumae Radix] 10g, *Acorus* *tatarinowii* Schott [Acoraceae; Acori Tatarinowii Rhizoma] 10g, *Polygala* *tenuifolia* Willd. [Polygalaceae; Polygalae Radix] 10g, *Alpinia* *oxyphylla* Miq. [Zingiberaceae; Alpiniae Oxyphyllae Fructus] 10g, *Angelica* *sinensis* (Oliv.) Diels [Apiaceae; Angelicae Sinensis Radix]10g, *Salvia* *miltiorrhiza* Bunge [Lamiaceae; Salviae Miltiorrhizae Radix et Rhizoma] 15g, *Spatholobus* *suberectus* Dunn [Fabaceae; Spatholobi Caulis] 15g, *Pheretima* *aspergillum* (E. Perrier) [Megascolecidae; Pheretima] 6g, *Siegesbeckia* *orientalis* L. [Asteraceae; Siegesbeckiae Herba] 10g, *Allium* *fistulosum* L. [Amaryllidaceae; Allii Fistulosi Caulis] 4 specifies | NR | NR | Control groups (Edaravone; tid; Injection; ivgtt + Citicoline Sodium; qd; Injection; ivgtt) |
| Zhao 2024 | Shenrong Tongmai Capsule (4caps, tid; capsule; oral) | *(Panax* *ginseng* C.A.Mey. [Araliaceae; Ginseng Radix et Rhizoma], *Styrax* *tonkinensis* (Pierre) Craib ex Hartw. [Styracaceae; Benzoinum], *Daemonorops* *draco* Bl. [Arecaceae; Sanguis Draconis], *Cervus* *elaphus / Cervus nippon* Linnaeus / Temminck [Cervidae; Cervi Cornu], *Whitmania pigra* Whitman [Hirudinidae; Hirudo], *Ligusticum* *chuanxiong* Hort. [Apiaceae; Chuanxiong Rhizoma], *Crataegus* *pinnatifida* Bunge [Rosaceae; Crataegi Fructus], *Rheum* *palmatum* L. [Polygonaceae; Rhei Radix et Rhizoma Praeparata]) NR | NR | NR | ct |
| Zhou H 2019 | Tongxinluo capsule (0.78g, tid; capsule; oral) | *(Panax ginseng* C.A.Mey. [Araliaceae; Ginseng Radix et Rhizoma], *Whitmania pigra* Whitman [Hirudinidae; Hirudo], *Buthus martensii* Karsch [Buthidae; Scorpio], *Paeonia veitchii* Lynch. [Ranunculaceae; Paeoniae Radix Rubra], *Cryptotympana pustulata* Fabricius [Cicadidae; Cicadae Periostracum], *Eupolyphaga sinensis* Walker [Corydiidae; Eupolyphaga seu Steleophaga], *Scolopendra subspinipes* mutilans L. Koch [Scolopendridae; Scolopendra], *Santalum album* L. [Santalaceae; Santali Albi Lignum], *Dalbergia odorifera* T.Chen [Fabaceae; Dalbergiae Odoriferae Lignum], *Boswellia carterii* Birdw. [Burseraceae; Olibanum], *Ziziphus jujuba* Mill. var. spinosa (Bunge) Hu ex H.F.Chow [Rhamnaceae; Ziziphi Spinosae Semen], *Dryobalanops aromatica* C. F. Gaertn. [Dipterocarpaceae; Borneolum Syntheticum]) NA | Indication: Invigorating qi and promoting blood circulation, dredging collaterals and relieving pain. Used for coronary heart disease and angina pectoris with heart qi deficiency and blood stasis obstructing collaterals, manifested as chest oppression, stabbing pain, colicky pain, fixed pain, palpitations, spontaneous sweating, shortness of breath and fatigue, dark purple tongue or ecchymosis, fine and rough or intermittent pulse. Also used for stroke of qi deficiency and blood stasis obstructing collaterals type, manifested as hemiplegia or unilateral numbness, deviated mouth and tongue, slurred speech; Adverse reactions: Occasional gastrointestinal discomfort such as nausea, vomiting, abdominal pain, abdominal distension, diarrhea, and stomach discomfort, as well as rash, itching, dizziness, etc.; Contraindications: Hemorrhagic diseases, pregnant women, women during menstruation, and yin deficiency with excessive fire type stroke are contraindicated; Shijiazhuang Yiling Pharmaceutical Co., Ltd.; Z19980015 | Grind Scorpion, Leech, Centipede, Ground Beetle, and Cicada Slough into ultrafine powder below 10 μm. Distill Sandalwood and Dalbergia odorifera with water vapor to collect volatile oil; the residue is then boiled in water, filtered, and concentrated under vacuum. Soak Ginseng in 70% ethanol, reflux-extract, recover the ethanol, and concentrate under vacuum; the residue is then boiled in water, filtered, and concentrated under vacuum. Boil Sour Jujube Seed and Red Peony Root in water, filter, and concentrate under vacuum. Combine all concentrated extracts, mix thoroughly with the ultrafine powder, spray-dry, and grind. Add an appropriate amount of excipient to form granules. Incorporate the volatile oil from Sandalwood and Dalbergia odorifera, mix well with Borneol, and encapsulate to obtain the final product. | Control groups (Butylphthalide; tid; capsule; oral) |
| Zhou Y 2021 | Yangxue Qingnao Granule (4g, tid; granule; oral) | *(Panax ginseng* C.A.Mey. [Araliaceae; Ginseng Radix et Rhizoma], *Whitmania pigra* Whitman [Hirudinidae; Hirudo], *Buthus martensii* Karsch [Buthidae; Scorpio], *Paeonia veitchii* Lynch. [Ranunculaceae; Paeoniae Radix Rubra], *Cryptotympana pustulata* Fabricius [Cicadidae; Cicadae Periostracum], *Eupolyphaga sinensis* Walker [Corydiidae; Eupolyphaga seu Steleophaga], *Scolopendra subspinipes* mutilans L. Koch [Scolopendridae; Scolopendra], *Santalum album* L. [Santalaceae; Santali Albi Lignum], *Dalbergia odorifera* T.Chen [Fabaceae; Dalbergiae Odoriferae Lignum], *Boswellia carterii* Birdw. [Burseraceae; Olibanum], *Ziziphus jujuba* Mill. var. spinosa (Bunge) Hu ex H.F.Chou [Rhamnaceae; Ziziphi Spinosae Semen], *Dryobalanops aromatica* C. F. Gaertn. [Dipterocarpaceae; Borneolum Syntheticum]) NA | Indication: Nourishing blood and calming the liver, promoting blood circulation and dredging collaterals. Used for headache, dizziness and blurred vision, irritability and anger, insomnia and dreaminess caused by blood deficiency and liver hyperactivity; Adverse reactions: Post-marketing monitoring data show that this product may cause the following adverse reactions: rash, itching, nausea, vomiting, abdominal distension, diarrhea, abdominal pain, gastric burning sensation, dry mouth, dizziness, headache, head distension, tinnitus, palpitations, blood pressure reduction, abnormal liver biochemical indicators, etc.; Contraindications: Pregnant women are contraindicated; patients with decompensated liver function are contraindicated; those allergic to this product and its ingredients are contraindicated; Tasly Pharmaceutical Group Co., Ltd.; Z10960082 | Grind Angelica sinensis, Ligusticum chuanxiong, Paeonia lactiflora, Rehmannia glutinosa, Uncaria rhynchophylla, Spatholobus suberectus, Prunella vulgaris, Cassia obtusifolia, and Corydalis yanhusuo into coarse powder. Decoct Concha margaritifera and Asarum heterotropoides with water twice, first with 10-fold volume for 2 hours, second with 8-fold volume for 1.5 hours. Combine the decoction, filter, and concentrate under vacuum to relative density 1.25–1.30 at 60°C. Add the above coarse powder to the concentrated extract, mix thoroughly, and granulate with an appropriate amount of dextrin and stevioside. Dry at low temperature, sift, and package to obtain the final product. | Control groups (NBP; bid; tablet; oral) |

**Note:** For each study, the information sources for indications, adverse reactions, and contraindications were reported separately as reported in primary RCT, external product source, or NR. Reported in primary RCT denotes information explicitly reported in the original randomized controlled trial; external product source denotes information obtained outside the primary RCT, including product labels/package inserts, manufacturer information, or regulatory registration records; and NR denotes not reported. Across the 50 included studies, most source entries were either external product sources or not reported. For commercial Chinese polyherbal preparations (CCPPs), dose was coded as NA because the dose of the proprietary formulation could not be directly converted to the doses of its individual medicinal materials. For non-commercial preparations, dose was coded as NR when it was not reported in the original article. Abbreviations: RCT, randomized controlled trial; CCPP, commercial Chinese polyherbal preparation; NA, not applicable; NR, not reported.

**Table S3. ConPhyMP-based characterization of Chinese medicine interventions**

| **Study ID** | **Chinese medicine intervention** | | **Type of extract** | **Preferred/main methods and chemical characterization results** | **Alternative methods and chemical characterization results** | **Reference** |
| --- | --- | --- | --- | --- | --- | --- |
| Chen 2014 | Tongxinluo Capsule | A | | Chinese Pharmacopoeia HPLC assay (preferred/main): use a C18 column with acetonitrile–water (15:85), detect at 230 nm, and require at least 3,000 theoretical plates for paeoniflorin. Extract about 0.25 g of mixed capsule contents with 25 mL of 70% methanol by ultrasonication (250 W, 40 kHz) for 50 min, make up the lost mass, filter, dilute 10 mL to 25 mL, and inject 10 µL. Paeoniflorin is identified by reference-standard retention time and quantified by external standard; the pharmacopoeial result is not less than 0.30 mg per capsule. | Chinese Pharmacopoeia TLC identification (alternative): (i) a water extract on polyamide film identifies Hirudo by a matching fluorescent principal zone at UV 254 nm; (ii) a D101-resin-cleaned extract on silica gel G identifies ginsenosides Rg1, Re and Rb1 by matching coloured zones after vanillin–sulfuric acid derivatisation; (iii) separate TLC tests identify borneol and Olibanum. In each test, the sample shows a zone at the same position and with the same colour/fluorescence as the corresponding reference. | Invention Patent: CN1198332A |
| Chen S.N 2019 | Huayu Tongluo Decoction | NR | | NR | NR | NR |
| Chen 2020 | Naokang Granule | NR | | NR | NR | NR |
| Chen K.L 2023 | Buyang Huanwu Decoction | NR | | NR | NR | NR |
| Chen S.Y 2025 | Bushen Yizhi Formula Decoction | NR | | NR | NR | NR |
| Chen B 2023 | Huayu Tongluo Decoction | NR | | NR | NR | NR |
| Ding 2017 | Fufang Congrong Yizhi Capsule | A | | NR | NR | NR |
| Gao 2023 | Naoxinkang Decoction | NR | | NR | NR | NR |
| Gao 2025 | Kongsheng Zhenzhong Dan Decoction | NR | | NR | NR | NR |
| Guo 2019 | Yangxue Qingnao Granules | A | | Chinese Pharmacopoeia HPLC assay (preferred/main): use a C18 column with isopropanol–methanol–5 mmol/L citric-acid solution (2:18:80), detect at 240 nm, and require at least 2,000 theoretical plates for paeoniflorin. Ultrasonicate about 0.08 g of granules with 5 mL of 0.2% sodium bicarbonate for 5 min, pass through a D101 macroporous-resin column, wash with water, elute with methanol, make to 10 mL, centrifuge and filter. Paeoniflorin is identified by reference-standard retention time and quantified; the result is not less than 9.0 mg per sachet. | Chinese Pharmacopoeia TLC identification (alternative): silica-gel TLC identifies (i) Prunellae Spica and rosmarinic acid using cyclohexane–ethyl acetate–formic acid (2:4:0.2) with UV 365 nm observation, (ii) Corydalis Rhizoma and tetrahydropalmatine, (iii) Cassiae Semen and chrysophanol, and (iv) Angelicae Sinensis Radix plus Chuanxiong Rhizoma using cyclohexane–ethyl acetate (4:1). The sample shows the same-position, same-colour or same-fluorescence zones as the corresponding references. | Invention Patent: ZL93100050.5 |
| He 2018 | Ginkgo Biloba Leaf Capsule | A | | Chinese Pharmacopoeia dual HPLC assays (preferred/main): (1) after 30 min acid hydrolysis in methanol–25% hydrochloric acid (4:1), separate on C18 with methanol–0.4% phosphoric acid (50:50), detect at 360 nm, determine quercetin, kaempferol and isorhamnetin using the prescribed relative-retention/correction factors, and calculate total flavonol glycosides as their sum × 2.51; (2) determine bilobalide and ginkgolides A, B and C by C18 HPLC-ELSD with n-propanol–tetrahydrofuran–water (1:15:84) and an external two-point logarithmic equation. Apply the dosage-form/strength-specific limits: commonly 9.6 mg flavonol glycosides plus 2.4 mg terpene lactones, or 19.2 mg plus 4.8 mg, per tablet/capsule. | Chinese Pharmacopoeia identification (alternative): silica-gel G TLC of the n-butanol extract against Ginkgo biloba reference extract, developed with ethyl acetate–butanone–formic acid–water (5:3:1:1) and derivatised with 3% aluminium chloride, gives corresponding visible and UV 365 nm fluorescent zones. In addition, the terpene-lactone chromatogram must show peaks at the retention times of the Ginkgo total-lactone reference extract, confirming bilobalide and ginkgolides A, B and C. | Invention Patent: CN201410389198 |
| Huang 2017 | Huayu Tongluo Decoction | NR | | NR | NR | NR |
| Huang H.Y 2019 | Yangxue Qingnao Granules | A | | Chinese Pharmacopoeia HPLC assay (preferred/main): use a C18 column with isopropanol–methanol–5 mmol/L citric-acid solution (2:18:80), detect at 240 nm, and require at least 2,000 theoretical plates for paeoniflorin. Ultrasonicate about 0.08 g of granules with 5 mL of 0.2% sodium bicarbonate for 5 min, pass through a D101 macroporous-resin column, wash with water, elute with methanol, make to 10 mL, centrifuge and filter. Paeoniflorin is identified by reference-standard retention time and quantified; the result is not less than 9.0 mg per sachet. | Chinese Pharmacopoeia TLC identification (alternative): silica-gel TLC identifies (i) Prunellae Spica and rosmarinic acid using cyclohexane–ethyl acetate–formic acid (2:4:0.2) with UV 365 nm observation, (ii) Corydalis Rhizoma and tetrahydropalmatine, (iii) Cassiae Semen and chrysophanol, and (iv) Angelicae Sinensis Radix plus Chuanxiong Rhizoma using cyclohexane–ethyl acetate (4:1). The sample shows the same-position, same-colour or same-fluorescence zones as the corresponding references. | Invention Patent: ZL93100050.5 |
| Huang PZ 2017 | Yishen Tongluo Huazhuo Decoction | NR | | NR | NR | NR |
| Ji 2016 | Qingnao Yizhi Granules | NR | | NR | NR | NR |
| Jia 2019 | Naokang Granules | NR | | NR | NR | NR |
| Jiang 2021 | Wanshi Qingxin Huatan Granules | NR | | NR | NR | NR |
| Kong 2022 | Yangxue Qingnao Granules | A | | Chinese Pharmacopoeia HPLC assay (preferred/main): use a C18 column with isopropanol–methanol–5 mmol/L citric-acid solution (2:18:80), detect at 240 nm, and require at least 2,000 theoretical plates for paeoniflorin. Ultrasonicate about 0.08 g of granules with 5 mL of 0.2% sodium bicarbonate for 5 min, pass through a D101 macroporous-resin column, wash with water, elute with methanol, make to 10 mL, centrifuge and filter. Paeoniflorin is identified by reference-standard retention time and quantified; the result is not less than 9.0 mg per sachet. | Chinese Pharmacopoeia TLC identification (alternative): silica-gel TLC identifies (i) Prunellae Spica and rosmarinic acid using cyclohexane–ethyl acetate–formic acid (2:4:0.2) with UV 365 nm observation, (ii) Corydalis Rhizoma and tetrahydropalmatine, (iii) Cassiae Semen and chrysophanol, and (iv) Angelicae Sinensis Radix plus Chuanxiong Rhizoma using cyclohexane–ethyl acetate (4:1). The sample shows the same-position, same-colour or same-fluorescence zones as the corresponding references. | Invention Patent: ZL93100050.5 |
| Li 2018 | Huayu Tongluo Decoction | NR | | NR | NR |  |
| Li M.J 2025 | Yangxue Qingnao Granules | A | | Chinese Pharmacopoeia HPLC assay (preferred/main): use a C18 column with isopropanol–methanol–5 mmol/L citric-acid solution (2:18:80), detect at 240 nm, and require at least 2,000 theoretical plates for paeoniflorin. Ultrasonicate about 0.08 g of granules with 5 mL of 0.2% sodium bicarbonate for 5 min, pass through a D101 macroporous-resin column, wash with water, elute with methanol, make to 10 mL, centrifuge and filter. Paeoniflorin is identified by reference-standard retention time and quantified; the result is not less than 9.0 mg per sachet. | Chinese Pharmacopoeia TLC identification (alternative): silica-gel TLC identifies (i) Prunellae Spica and rosmarinic acid using cyclohexane–ethyl acetate–formic acid (2:4:0.2) with UV 365 nm observation, (ii) Corydalis Rhizoma and tetrahydropalmatine, (iii) Cassiae Semen and chrysophanol, and (iv) Angelicae Sinensis Radix plus Chuanxiong Rhizoma using cyclohexane–ethyl acetate (4:1). The sample shows the same-position, same-colour or same-fluorescence zones as the corresponding references. | Invention Patent: ZL93100050.5 |
| Li L 2022 | Fufang Congrong Yizhi Capsule | A | | National Drug Registration Standard YBZ00852008 HPLC assay (preferred/main): use a C18 column with methanol–acetonitrile–water–phosphoric acid (10:20:110:0.01), detect at 320 nm, and require at least 2,500 theoretical plates for 2,3,5,4′-tetrahydroxystilbene-2-O-β-D-glucoside. Ultrasonicate about 0.15 g of capsule powder with 20 mL methanol for 20 min, make up the lost mass, filter through a 0.45 µm membrane, and inject 10 µL. Identify the marker by comparison with the reference standard and quantify it by HPLC; the specified limit is not less than 0.70 mg per capsule. | National Drug Registration Standard YBZ00852008 TLC identification (alternative): prepare the prescribed methanolic or solvent-partitioned extracts and compare them on silica-gel plates with Polygoni Multiflori Radix, Nelumbinis Folium, Cistanches Herba, Pheretima and Rhapontici Radix references. The preparation is identified when the sample chromatograms show characteristic zones at the same positions and with the same colours or fluorescence as the corresponding references for the five medicinal materials. | Invention Patent: CN102914615A |
| Liang 2020 | Yangxue Qingnao Granule | A | | Chinese Pharmacopoeia HPLC assay (preferred/main): use a C18 column with isopropanol–methanol–5 mmol/L citric-acid solution (2:18:80), detect at 240 nm, and require at least 2,000 theoretical plates for paeoniflorin. Ultrasonicate about 0.08 g of granules with 5 mL of 0.2% sodium bicarbonate for 5 min, pass through a D101 macroporous-resin column, wash with water, elute with methanol, make to 10 mL, centrifuge and filter. Paeoniflorin is identified by reference-standard retention time and quantified; the result is not less than 9.0 mg per sachet. | Chinese Pharmacopoeia TLC identification (alternative): silica-gel TLC identifies (i) Prunellae Spica and rosmarinic acid using cyclohexane–ethyl acetate–formic acid (2:4:0.2) with UV 365 nm observation, (ii) Corydalis Rhizoma and tetrahydropalmatine, (iii) Cassiae Semen and chrysophanol, and (iv) Angelicae Sinensis Radix plus Chuanxiong Rhizoma using cyclohexane–ethyl acetate (4:1). The sample shows the same-position, same-colour or same-fluorescence zones as the corresponding references. | Invention Patent: ZL93100050.5 |
| Liu H.S 2022 | Nao An Capsule | A | | Chinese Pharmacopoeia HPLC assays (preferred/main): (1) for ferulic acid from Angelica/Chuanxiong, use C18 with methanol–0.6 mol/L acetic acid (30:70), detect at 320 nm, extract about 4 g by Soxhlet with methanol, acidify the aqueous residue to pH 1–2 and partition into ether; the result is not less than 60 µg ferulic acid per capsule. (2) for ginseng, use C18 with acetonitrile–water (20:80), detect at 203 nm and require at least 10,000 plates for ginsenoside Re; after chloroform defatting, extract with water-saturated n-butanol, wash, evaporate and reconstitute in methanol. Ginsenoside Re is not less than 0.20 mg per capsule. | Chinese Pharmacopoeia TLC identification (alternative): silica-gel TLC of the alkalised chloroform extract shows UV 365 nm fluorescent zones corresponding to both Chuanxiong Rhizoma and Angelicae Sinensis Radix references. A second TLC test of the n-butanol fraction, developed at low temperature and visualised with sulfuric-acid ethanol, shows corresponding purple-red zones for ginsenoside Re and Rg1 reference substances. | Invention Patent: ZL200310103217.7 |
| Liu X.F 2025 | Shenwu Yizhi Capsule | NR | | NR | NR | NR |
| Lu 2017 | Chaiqin Wendan Decoction | NR | | NR | NR | NR |
| Lu 2024 | Wenyang Jiangzhuo Granule | NR | | NR | NR | NR |
| Ma 2020 | Yangxue Qingnao Granule | A | | Chinese Pharmacopoeia HPLC assay (preferred/main): use a C18 column with isopropanol–methanol–5 mmol/L citric-acid solution (2:18:80), detect at 240 nm, and require at least 2,000 theoretical plates for paeoniflorin. Ultrasonicate about 0.08 g of granules with 5 mL of 0.2% sodium bicarbonate for 5 min, pass through a D101 macroporous-resin column, wash with water, elute with methanol, make to 10 mL, centrifuge and filter. Paeoniflorin is identified by reference-standard retention time and quantified; the result is not less than 9.0 mg per sachet. | Chinese Pharmacopoeia TLC identification (alternative): silica-gel TLC identifies (i) Prunellae Spica and rosmarinic acid using cyclohexane–ethyl acetate–formic acid (2:4:0.2) with UV 365 nm observation, (ii) Corydalis Rhizoma and tetrahydropalmatine, (iii) Cassiae Semen and chrysophanol, and (iv) Angelicae Sinensis Radix plus Chuanxiong Rhizoma using cyclohexane–ethyl acetate (4:1). The sample shows the same-position, same-colour or same-fluorescence zones as the corresponding references. | Invention Patent: ZL93100050.5 |
| Meng 2020 | Wenxiao Ⅲ Decoction | NR | | NR | NR | NR |
| Qu 2019 | Gingko Biloba Leaf capsule | A | | Chinese Pharmacopoeia dual HPLC assays (preferred/main): (1) after 30 min acid hydrolysis in methanol–25% hydrochloric acid (4:1), separate on C18 with methanol–0.4% phosphoric acid (50:50), detect at 360 nm, determine quercetin, kaempferol and isorhamnetin using the prescribed relative-retention/correction factors, and calculate total flavonol glycosides as their sum × 2.51; (2) determine bilobalide and ginkgolides A, B and C by C18 HPLC-ELSD with n-propanol–tetrahydrofuran–water (1:15:84) and an external two-point logarithmic equation. Apply the dosage-form/strength-specific limits: commonly 9.6 mg flavonol glycosides plus 2.4 mg terpene lactones, or 19.2 mg plus 4.8 mg, per tablet/capsule. | Chinese Pharmacopoeia identification (alternative): silica-gel G TLC of the n-butanol extract against Ginkgo biloba reference extract, developed with ethyl acetate–butanone–formic acid–water (5:3:1:1) and derivatised with 3% aluminium chloride, gives corresponding visible and UV 365 nm fluorescent zones. In addition, the terpene-lactone chromatogram must show peaks at the retention times of the Ginkgo total-lactone reference extract, confirming bilobalide and ginkgolides A, B and C. | Pharmaceutical Standard: Z20040097 |
| Qu 2020 | Gingko Biloba Leaf capsule | A | | Chinese Pharmacopoeia dual HPLC assays (preferred/main): (1) after 30 min acid hydrolysis in methanol–25% hydrochloric acid (4:1), separate on C18 with methanol–0.4% phosphoric acid (50:50), detect at 360 nm, determine quercetin, kaempferol and isorhamnetin using the prescribed relative-retention/correction factors, and calculate total flavonol glycosides as their sum × 2.51; (2) determine bilobalide and ginkgolides A, B and C by C18 HPLC-ELSD with n-propanol–tetrahydrofuran–water (1:15:84) and an external two-point logarithmic equation. Apply the dosage-form/strength-specific limits: commonly 9.6 mg flavonol glycosides plus 2.4 mg terpene lactones, or 19.2 mg plus 4.8 mg, per tablet/capsule. | Chinese Pharmacopoeia identification (alternative): silica-gel G TLC of the n-butanol extract against Ginkgo biloba reference extract, developed with ethyl acetate–butanone–formic acid–water (5:3:1:1) and derivatised with 3% aluminium chloride, gives corresponding visible and UV 365 nm fluorescent zones. In addition, the terpene-lactone chromatogram must show peaks at the retention times of the Ginkgo total-lactone reference extract, confirming bilobalide and ginkgolides A, B and C. | Pharmaceutical Standard: 20010169 |
| Ren 2022 | Peiyuan Kaizhi Decoction | NR | | NR | NR | NR |
| Ruan 2023 | Yangxue Qingnao Granule | A | | Chinese Pharmacopoeia HPLC assay (preferred/main): use a C18 column with isopropanol–methanol–5 mmol/L citric-acid solution (2:18:80), detect at 240 nm, and require at least 2,000 theoretical plates for paeoniflorin. Ultrasonicate about 0.08 g of granules with 5 mL of 0.2% sodium bicarbonate for 5 min, pass through a D101 macroporous-resin column, wash with water, elute with methanol, make to 10 mL, centrifuge and filter. Paeoniflorin is identified by reference-standard retention time and quantified; the result is not less than 9.0 mg per sachet. | Chinese Pharmacopoeia TLC identification (alternative): silica-gel TLC identifies (i) Prunellae Spica and rosmarinic acid using cyclohexane–ethyl acetate–formic acid (2:4:0.2) with UV 365 nm observation, (ii) Corydalis Rhizoma and tetrahydropalmatine, (iii) Cassiae Semen and chrysophanol, and (iv) Angelicae Sinensis Radix plus Chuanxiong Rhizoma using cyclohexane–ethyl acetate (4:1). The sample shows the same-position, same-colour or same-fluorescence zones as the corresponding references. | Invention Patent: ZL93100050.5 |
| Shi 2017 | Gingko Biloba Leaf tablet | A | | Chinese Pharmacopoeia dual HPLC assays (preferred/main): (1) after 30 min acid hydrolysis in methanol–25% hydrochloric acid (4:1), separate on C18 with methanol–0.4% phosphoric acid (50:50), detect at 360 nm, determine quercetin, kaempferol and isorhamnetin using the prescribed relative-retention/correction factors, and calculate total flavonol glycosides as their sum × 2.51; (2) determine bilobalide and ginkgolides A, B and C by C18 HPLC-ELSD with n-propanol–tetrahydrofuran–water (1:15:84) and an external two-point logarithmic equation. Apply the dosage-form/strength-specific limits: commonly 9.6 mg flavonol glycosides plus 2.4 mg terpene lactones, or 19.2 mg plus 4.8 mg, per tablet/capsule. | Chinese Pharmacopoeia identification (alternative): silica-gel G TLC of the n-butanol extract against Ginkgo biloba reference extract, developed with ethyl acetate–butanone–formic acid–water (5:3:1:1) and derivatised with 3% aluminium chloride, gives corresponding visible and UV 365 nm fluorescent zones. In addition, the terpene-lactone chromatogram must show peaks at the retention times of the Ginkgo total-lactone reference extract, confirming bilobalide and ginkgolides A, B and C. | Invention Patent: CN201410389198 |
| Sun X.J 2023 | Fufang Congrong Yizhi Capsule | A | | National Drug Registration Standard YBZ00852008 HPLC assay (preferred/main): use a C18 column with methanol–acetonitrile–water–phosphoric acid (10:20:110:0.01), detect at 320 nm, and require at least 2,500 theoretical plates for 2,3,5,4′-tetrahydroxystilbene-2-O-β-D-glucoside. Ultrasonicate about 0.15 g of capsule powder with 20 mL methanol for 20 min, make up the lost mass, filter through a 0.45 µm membrane, and inject 10 µL. Identify the marker by comparison with the reference standard and quantify it by HPLC; the specified limit is not less than 0.70 mg per capsule. | National Drug Registration Standard YBZ00852008 TLC identification (alternative): prepare the prescribed methanolic or solvent-partitioned extracts and compare them on silica-gel plates with Polygoni Multiflori Radix, Nelumbinis Folium, Cistanches Herba, Pheretima and Rhapontici Radix references. The preparation is identified when the sample chromatograms show characteristic zones at the same positions and with the same colours or fluorescence as the corresponding references for the five medicinal materials. | Invention Patent: CN102914615A |
| Wang J 2023 | Tongqiao Huoxue Decoction | NR | | NR | NR | NR |
| Wang 2024 | Gingko Biloba Leaf capsule | A | | Chinese Pharmacopoeia dual HPLC assays (preferred/main): (1) after 30 min acid hydrolysis in methanol–25% hydrochloric acid (4:1), separate on C18 with methanol–0.4% phosphoric acid (50:50), detect at 360 nm, determine quercetin, kaempferol and isorhamnetin using the prescribed relative-retention/correction factors, and calculate total flavonol glycosides as their sum × 2.51; (2) determine bilobalide and ginkgolides A, B and C by C18 HPLC-ELSD with n-propanol–tetrahydrofuran–water (1:15:84) and an external two-point logarithmic equation. Apply the dosage-form/strength-specific limits: commonly 9.6 mg flavonol glycosides plus 2.4 mg terpene lactones, or 19.2 mg plus 4.8 mg, per tablet/capsule. | Chinese Pharmacopoeia identification (alternative): silica-gel G TLC of the n-butanol extract against Ginkgo biloba reference extract, developed with ethyl acetate–butanone–formic acid–water (5:3:1:1) and derivatised with 3% aluminium chloride, gives corresponding visible and UV 365 nm fluorescent zones. In addition, the terpene-lactone chromatogram must show peaks at the retention times of the Ginkgo total-lactone reference extract, confirming bilobalide and ginkgolides A, B and C. | Invention Patent: CN103520225B |
| Wu 2024 (Wu et al., 2024) | Peiyuan Tongnao Capsule | A | | Chinese Pharmacopoeia HPLC assay (preferred/main; protect from light): use a C18 column with acetonitrile–water (15:85), detect at 320 nm, and require at least 6,000 theoretical plates for 2,3,5,4′-tetrahydroxystilbene-2-O-β-D-glucoside. Reflux about 0.6 g of capsule powder with 25 mL dilute ethanol for 30 min, make up the lost mass, filter and inject 10 µL against a 25 µg/mL reference solution. The identified marker is not less than 0.46 mg per capsule. | Chinese Pharmacopoeia identity tests (alternative): microscopy confirms characteristic particles from Cinnamomi Cortex, Scorpio, Rehmanniae Radix Praeparata and Cervi Cornu Pantotrichum. TLC confirms Polygoni Multiflori Radix, cinnamaldehyde, paeoniflorin and echinacoside: the sample shows same-position, same-colour zones (or, for echinacoside, the same UV 365 nm fluorescent zone) as the corresponding reference material. | Pharmaceutical Standard: Z20000022 |
| Xiao 2021 | Ginkgo Biloba Extract | A | | Chinese Pharmacopoeia chemical characterisation (preferred/main): use C18 HPLC at 360 nm to determine quercetin, kaempferol and isorhamnetin and calculate total flavonol glycosides as their sum × 2.51; use C18 HPLC-ELSD with n-propanol–tetrahydrofuran–water (1:15:84) to determine bilobalide and ginkgolides A, B and C by the external two-point logarithmic equation. A compliant dry extract contains not less than 24.0% total flavonol glycosides and not less than 6.0% total terpene lactones; total ginkgolic acids are controlled at not more than 5 mg/kg. | Chinese Pharmacopoeia fingerprint/TLC (alternative): the C18 HPLC fingerprint uses acetonitrile and 0.4% phosphoric acid gradient elution with rutin and Ginkgo reference extract, and the sample profile is assessed against the prescribed characteristic peaks/relative retention times. Silica-gel G TLC with ethyl acetate–butanone–formic acid–water (5:3:1:1), followed by aluminium-chloride derivatisation, shows corresponding fluorescent zones at UV 365 nm, supporting extract identity. | Invention Patent: EP431535B1 |
| Xie 2020 | Yangxue QingnaoTablet | A | | No Chinese Pharmacopoeia monograph for a dosage form named “Yangxue Qingnao Tablet” was identified. The closest authoritative formulation-level evidence is the peer-reviewed UPLC-Q-TOF/MSE study of Yangxue Qingnao Granules: extract 1.5 g with 20 mL of 50% methanol by ultrasonication for 30 min and filter at 0.22 µm; separate on an ACQUITY BEH C18 column (100 × 2.1 mm, 1.7 µm) with a 0.1% formic-acid water/acetonitrile gradient at 0.25 mL/min and 35 °C; acquire positive/negative high-resolution MSE spectra over m/z 50–1,200. Thirty-nine metabolites were confirmed with reference standards and 103 were tentatively assigned, giving 142 identified/tentatively identified metabolites from the 11 botanical drugs. | Peer-reviewed SPE-UPLC assay for the granule—not an exact tablet monograph (alternative): purify the sample on a C18 solid-phase extraction cartridge, separate on a C18 UPLC column (50 × 2.1 mm, 1.7 µm) with acetonitrile–water (25:75) at 1.0 mL/min and 40 °C, and detect paeoniflorin at 230 nm. The method was linear over 5.0–80.0 µg/mL (r = 0.9999); mean recovery was 99.4% (RSD 2.83%). Applicability to a tablet requires confirmation. | Invention Patent: ZL93100050.5 |
| Xu 2025 | Zhishe Tongluo capsule | A | | Peer-reviewed UHPLC-Q Exactive Focus MS/MS profiling (preferred/main): ultrasonicate 100 mg of capsule powder with 10 mL methanol for 10 min (600 W, 40 kHz), filter at 0.22 µm, and separate on an Accucore aQ C18 column (150 × 2.1 mm, 2.6 µm) with methanol/0.1% formic acid gradient elution at 0.3 mL/min and 30 °C. Acquire positive/negative Full MS/dd-MS2 data over m/z 100–1,500 at 70,000/35,000 resolution and identify by accurate mass, fragments and an in-house database. Seventy metabolites were identified in the capsule extract. | Peer-reviewed in vivo metabolite tracking (alternative): after oral dosing in normal and cerebral-ischaemia/reperfusion rats, precipitate plasma/brain homogenate with acetonitrile and analyse by the same UHPLC-HRMS method. Twenty metabolites were detected in plasma and seven crossed into brain tissue; representative discriminating metabolites included calycosin-7-glucoside, curcumenol, senkyunolide B/I, dihydrotanshinone I, cryptotanshinone and salvianolic acid A. | Pharmaceutical Standard: Z20090658 |
| Yang P 2016 | Tongxinluo Capsule | A | | Chinese Pharmacopoeia HPLC assay (preferred/main): use a C18 column with acetonitrile–water (15:85), detect at 230 nm, and require at least 3,000 theoretical plates for paeoniflorin. Extract about 0.25 g of mixed capsule contents with 25 mL of 70% methanol by ultrasonication (250 W, 40 kHz) for 50 min, make up the lost mass, filter, dilute 10 mL to 25 mL, and inject 10 µL. Paeoniflorin is identified by reference-standard retention time and quantified by external standard; the pharmacopoeial result is not less than 0.30 mg per capsule. | Chinese Pharmacopoeia TLC identification (alternative): (i) a water extract on polyamide film identifies Hirudo by a matching fluorescent principal zone at UV 254 nm; (ii) a D101-resin-cleaned extract on silica gel G identifies ginsenosides Rg1, Re and Rb1 by matching coloured zones after vanillin–sulfuric acid derivatisation; (iii) separate TLC tests identify borneol and Olibanum. In each test, the sample shows a zone at the same position and with the same colour/fluorescence as the corresponding reference. | Invention Patent: CN1198332A |
| Yang 2016 | Yangxue Qingnao Granule | A | | Chinese Pharmacopoeia HPLC assay (preferred/main): use a C18 column with isopropanol–methanol–5 mmol/L citric-acid solution (2:18:80), detect at 240 nm, and require at least 2,000 theoretical plates for paeoniflorin. Ultrasonicate about 0.08 g of granules with 5 mL of 0.2% sodium bicarbonate for 5 min, pass through a D101 macroporous-resin column, wash with water, elute with methanol, make to 10 mL, centrifuge and filter. Paeoniflorin is identified by reference-standard retention time and quantified; the result is not less than 9.0 mg per sachet. | Chinese Pharmacopoeia TLC identification (alternative): silica-gel TLC identifies (i) Prunellae Spica and rosmarinic acid using cyclohexane–ethyl acetate–formic acid (2:4:0.2) with UV 365 nm observation, (ii) Corydalis Rhizoma and tetrahydropalmatine, (iii) Cassiae Semen and chrysophanol, and (iv) Angelicae Sinensis Radix plus Chuanxiong Rhizoma using cyclohexane–ethyl acetate (4:1). The sample shows the same-position, same-colour or same-fluorescence zones as the corresponding references. | Invention Patent: ZL93100050.5 |
| Zhang SX 2019 | Jiawei Dihuang Yin Decoction | NR | | NR | NR | NR |
| Zhang 2021 | Tongqiao Huoxue Decoction | NR | | NR | NR | NR |
| Zhang F.P 2025 | Fufang Huonaoshu Capsule | A | | National Drug Registration Standard YBZ00852008 HPLC assay (preferred/main): use a C18 column with methanol–acetonitrile–water–phosphoric acid (10:20:110:0.01), detect at 320 nm, and require at least 2,500 theoretical plates for 2,3,5,4′-tetrahydroxystilbene-2-O-β-D-glucoside. Ultrasonicate about 0.15 g of capsule powder with 20 mL methanol for 20 min, make up the lost mass, filter through a 0.45 µm membrane, and inject 10 µL. Identify the marker by comparison with the reference standard and quantify it by HPLC; the specified limit is not less than 0.70 mg per capsule. | National Drug Registration Standard YBZ00852008 TLC identification (alternative): prepare the prescribed methanolic or solvent-partitioned extracts and compare them on silica-gel plates with Polygoni Multiflori Radix, Nelumbinis Folium, Cistanches Herba, Pheretima and Rhapontici Radix references. The preparation is identified when the sample chromatograms show characteristic zones at the same positions and with the same colours or fluorescence as the corresponding references for the five medicinal materials. | Invention Patent: CN102914615A |
| Zhang DC 2019 | Zhilong Huoxue Tongyu capsule | A | | Peer-reviewed UPLC-HRMS characterisation (preferred/main): sonicate the capsule powder sequentially with 10-fold and 8-fold volumes of 70% ethanol for 0.5 h each, combine, concentrate and freeze-dry. Separate on a BEH C18 column (2.1 × 100 mm, 1.7 µm; 35 °C) with 0.1% formic-acid water/acetonitrile gradient elution at 0.2 mL/min, and acquire negative full-scan Q-Exactive HRMS data over m/z 70–1,050 at 70,000 resolution. Twelve metabolites were confirmed with authentic standards: salidroside, chlorogenic acid, L-epicatechin, calycosin-7-glucoside, ononin, calycosin, formononetin, astragalosides A/I/II/III and wogonin. | Independent peer-reviewed UPLC-Q-Orbitrap HRMS (alternative): prepare aqueous and ethanol extracts, separate on BEH C18 with 0.1% formic-acid water/acetonitrile at 0.2 mL/min, and acquire positive-ion spectra over m/z 150–1,500 at 70,000 resolution. Comparison with authentic standards confirmed eight representative metabolites: L-epicatechin, calycosin-7-O-β-glucoside, coumarin, ononin, calycosin, cinnamaldehyde, formononetin and wogonin. | Invention Patent: ZL200810147774.1 |
| Zhang XH 2021 | Kaiqiao Tongluo Decoction | NR | | NR |  | NR |
| Zhao 2024 | Shenrong Tongmai Capsule | NR | | NR |  | NR |
| Zhou H 2019 | Tongxinluo capsule | A | | Grind Scorpion, Leech, Centipede, Ground Beetle, and Cicada Slough into ultrafine powder below 10 μm. Distill Sandalwood and Dalbergia odorifera with water vapor to collect volatile oil; the residue is then boiled in water, filtered, and concentrated under vacuum. Soak Ginseng in 70% ethanol, reflux-extract, recover the ethanol, and concentrate under vacuum; the residue is then boiled in water, filtered, and concentrated under vacuum. Boil Sour Jujube Seed and Red Peony Root in water, filter, and concentrate under vacuum. Combine all concentrated extracts, mix thoroughly with the ultrafine powder, spray-dry, and grind. Add an appropriate amount of excipient to form granules. Incorporate the volatile oil from Sandalwood and Dalbergia odorifera, mix well with Borneol, and encapsulate to obtain the final product. |  | Invention Patent: CN1198332A |
| Zhou Y 2021 | Yangxue Qingnao Granule | A | | Grind Angelica sinensis, Ligusticum chuanxiong, Paeonia lactiflora, Rehmannia glutinosa, Uncaria rhynchophylla, Spatholobus suberectus, Prunella vulgaris, Cassia obtusifolia, and Corydalis yanhusuo into coarse powder. Decoct Concha margaritifera and Asarum heterotropoides with water twice, first with 10-fold volume for 2 hours, second with 8-fold volume for 1.5 hours. Combine the decoction, filter, and concentrate under vacuum to relative density 1.25–1.30 at 60°C. Add the above coarse powder to the concentrated extract, mix thoroughly, and granulate with an appropriate amount of dextrin and stevioside. Dry at low temperature, sift, and package to obtain the final product. |  | Invention Patent: ZL93100050.5 |
